# Pregnancy outcomes in women taking probiotics or prebiotics: a systematic review and meta-analysis

**DOI:** 10.1186/s12884-017-1629-5

**Published:** 2018-01-08

**Authors:** Alexander Jarde, Anne-Mary Lewis-Mikhael, Paul Moayyedi, Jennifer C. Stearns, Stephen M. Collins, Joseph Beyene, Sarah D. McDonald

**Affiliations:** 1 Department of Obstetrics and Gynecology, McMaster University, 1280 Main Street West, Hamilton, ON L8S 4K1 Canada; 2 Department of Medicine, Gastroenterology Division, McMaster University, Hamilton, ON Canada; 3 Farncombe Family Digestive Health Research Institute, McMaster University, Hamilton, ON Canada; 4 Farncombe Family Digestive Health Research Institute, Department of Medicine, McMaster University, Hamilton, ON Canada; 5 Department of Clinical Epidemiology and Biostatistics, McMaster University, Hamilton, ON Canada

**Keywords:** Prebiotics, Pregnancy, Preterm birth, Probiotics, Synbiotics

## Abstract

**Background:**

Probiotics are living microorganisms that, when administered in adequate amounts, confer a health benefit. It has been speculated that probiotics might help prevent preterm birth, but in two previous systematic reviews possible major increases in this risk have been suggested. Our objective was to perform a systematic review and meta-analysis of the risk of preterm birth and other adverse pregnancy outcomes in pregnant women taking probiotics, prebiotics or synbiotics.

**Methods:**

We searched six electronic databases (MEDLINE, EMBASE, CINAHL, Cochrane Central Register of Controlled Trials, Web of Science’s Core collection and BIOSIS Preview) up to September 2016 and contacted authors for additional data. We included randomized controlled trials in which women with a singleton pregnancy received a probiotic, prebiotic or synbiotic intervention. Two independent reviewers extracted data using a piloted form and assessed the risk of bias using the Cochrane risk of bias tool. We used random-effects meta-analyses to pool the results.

**Results:**

We identified 2574 publications, screened 1449 non-duplicate titles and abstracts and read 160 full text articles. The 49 publications that met our inclusion criteria represented 27 studies. No study used synbiotics, one used prebiotics and the rest used probiotics. Being randomized to take probiotics during pregnancy neither increased nor decreased the risk of preterm birth < 34 weeks (RR 1.03, 95% CI 0.29–3.64, I^2^ 0%, 1017 women in 5 studies), preterm birth < 37 weeks (RR 1.08, 95% CI 0.71–1.63, I^2^ 0%, 2484 women in 11 studies), or most of our secondary outcomes, including gestational diabetes mellitus.

**Conclusions:**

We found no evidence that taking probiotics or prebiotics during pregnancy either increases or decreases the risk of preterm birth or other infant and maternal adverse pregnancy outcomes.

**Trial registration:**

We prospectively published the protocol for this study in the PROSPERO database (CRD42016048129).

**Electronic supplementary material:**

The online version of this article (10.1186/s12884-017-1629-5) contains supplementary material, which is available to authorized users.

## Background

The probiotics industry exceeded $35 Billion in 2015 and it is expected to continue to grow rapidly in coming years [1]. Probiotics are living microorganisms that, when administered in adequate amounts, confer a health benefit by re-inoculating or balancing the host’s microflora, [2] prebiotics are non-digestible carbohydrates that nourish probiotics and healthy bacteria and synbiotics are combinations of probiotics and prebiotics. They can be given as biological supplements or in food [3] such as yogurt, [4] making them readily available for consumption. Probiotics have proven benefit for gastrointestinal disorders such as irritable bowel syndrome [5]. That said, there is uncertainty regarding what is the proper way of grouping (or not) different probiotic types and species [6].

Women of childbearing age are one of the commonest groups to take probiotics for gastrointestinal symptoms [7]. Among pregnant women, 1.3 to 3.6% use probiotics in the United States and Canada, and up to 13.7% do so in the Netherlands [8, 9]. It has been speculated that probiotics might help prevent preterm birth [10]. Intrauterine infection is a frequent and important factor in preterm birth and there is evidence supporting its role as an etiologic agent [11–18]. Probiotics can interfere with the processes that can lead to preterm labour by displacing and killing pathogens, through enhancement of anti-inflammatory cytokines and by reducing the pH to make the vaginal environment friendlier to beneficial bacteria [10]. Prebiotics would contribute to the beneficial effect of probiotics by stimulating their growth, activity, or both, [19, 20] and synbiotics combine probiotics and prebiotics. However, this is still mostly hypothetical and it is also possible that probiotics could be harmful to the infant as well as beneficial, requiring further studies.

One study analyzing data from a Norwegian cohort found a statistically significant protective effect of spontaneous preterm delivery in women with high intake of probiotic milk products (OR: 0.820; 95% CI: 0.681, 0.986) [21]. In contrast, two Cochrane reviews of randomized controlled trials, one on gestational diabetes and another focused on the association of probiotics with preterm labour, obtained relative risks between 3 and 4 (albeit not statistically significant) [19, 22]. In both reviews the results come from only one (the same) trial, [23] and although it is difficult to extrapolate data from high risk groups such as gestational diabetes, these data emphasize that probiotics could be associated with adverse outcomes and these also need to be addressed in an updated systematic review. One of the reviews [19] also identified a study comparing prebiotics with placebo, in which no significant differences were found in gestational age at birth [24].

Given the growing expansion of the probiotic industry and the ease with which probiotic products are available to the general public, there is a pressing need for an up to date assessment of the risk of preterm birth, including the large number of studies that have been published since the most recent previous review executed their search strategy in 2013. In addition, the risks of preterm birth in women taking prebiotics or synbiotics have yet to be systematically reviewed.

Our objective was to perform an up to date systematic review and meta-analysis of the risk of preterm birth and other adverse pregnancy outcomes in pregnant women randomized to probiotics, prebiotics or synbiotics.

## Methods

We prospectively published the protocol for this study in the PROSPERO database (CRD42016048129).

### Information sources and search strategy

We searched six electronic databases (MEDLINE, EMBASE, CINAHL, Cochrane Central Register of Controlled Trials, Web of Science’s Core collection and BIOSIS Preview) from their inception up to September 22, 2016, with no language restrictions (Please see Additional file 1: Appendix A for complete search strategies). Clinicaltrials.gov was also searched for ongoing trials.

### Selection criteria

We included randomized controlled trials in which pregnant women were allocated to an intervention group receiving any combinations of probiotics, prebiotics or synbiotics; or to a control group receiving no treatment, treatment as usual, placebo or any combination of probiotics, prebiotics or synbiotics. We excluded other study designs, as well as conference abstracts and studies including twins or higher order pregnancies (which are known to have a substantially higher risk of preterm birth and other adverse maternal and infant outcomes and are therefore not generalizable to average risk pregnancies). We contacted authors to clarify inclusion criteria, such as confirming the absence of twins if not specifically stated, and to ask for stratified data by singletons and other additional data as necessary.

Our primary outcomes were preterm birth < 34 weeks and preterm birth < 37 weeks. We defined a subset of key secondary outcomes that would be included in our subgroup analyses: gestational age at birth (continuous data), birth weight (continuous data), small and large for gestational age (< 10^th^ and > 90^th^ percentile for age and sex, respectively), gestational diabetes (GDM) and premature preterm rupture of membranes (PPROM). Other infant secondary outcomes included infant anthropometric measures (including birth weight, length, head circumference, etc.), other definitions (cut-off points) of preterm birth, neonatal death, different measures of adverse status at birth such as Neonatal Intensive Care Unit (NICU) admission, low Apgar score at 5 min and low umbilical cord pH. Other maternal secondary outcomes were maternal anthropometric measures (including gestational weight gain and changes in body mass index), infections (bacterial vaginosis, urinary tract infections), hypertension (including preeclampsia), gestational diabetes and other glucose metabolism related outcomes and caesarean section.

### Data extraction and assessment of risk of bias

Two reviewers (AJ and AMLM) independently screened all titles and abstracts and the full text of potentially eligible papers. Disagreements were resolved by discussion and a third person (SDM) was available if consensus could not be reached.

The same two reviewers independently extracted data on general study characteristics, intervention and control characteristics, potential effect modifiers, outcomes and risk of bias using a piloted data collection form. For binary data we extracted 2 by 2 tables or effect sizes (e.g. RR) with their confidence intervals. For continuous outcomes, we extracted the mean, standard deviation and size of each group or the mean difference and confidence interval. For measures of change we extracted means and standard deviations of the differences between the start and end points. In the cases where only before and after intervention data was provided, we imputed the standard deviation of the difference using the correlation of the largest study providing such information [25]. Studies in which probiotics were provided by the producing company without compensation were considered to have potential conflicts of interest even when the authors declared no such conflicts.

We assessed risk of bias using the Cochrane Risk of Bias tool, in which seven domains are considered for their risk of bias (high, low, or unclear): random sequence generation, allocation concealment, blinding of participants and personnel, blinding of outcome assessment, incomplete outcome data, selective reporting, and other biases [25]. Although the Cochrane risk of bias tool does not provide an overall risk of bias assessment, we applied the following algorithm: In order to consider a study as overall having low risk of bias we defined that it had to have none of the domains considered as high risk of bias and at least four (not counting ‘Other biases’) considered as low risk of bias, with at least one of them being ‘random sequence generation’ or ‘allocation concealment’.

### Data synthesis and statistical analyses

We performed pairwise inverse variance random effects meta-analyses (DerSimonian and Laird [26]) using Review Manager (version 5.3). Given the uncertainty in the field regarding the proper way of grouping (or not) the different interventions, [6] we decided to analyse the data at three different levels: 1) Pooling probiotics, prebiotics and synbiotics separately, 2) separating interventions by genus (i.e. studies using any species of *Lactobacillus*, *Bifidobacterium*, or *Streptococcus*, on their own or in combination with other types of probiotics) and 3) looking at each combination of species separately. Although we had planned further analyses in the protocol, they were considered to be uninformative given the characteristics of the identified studies.

We calculated pooled relative risks (RR) and mean differences (MD) with their correspondent 95% confidence intervals (CI) and quantified heterogeneity using the I-squared statistic (I^2^).

### Management of multiple comparisons

Whenever we encountered multiple, correlated comparisons (e.g. studies with two probiotics groups and one control group) we combined the intervention groups into a single group. In the subgroup analyses, if the different comparisons were in separate subgroups, the shared control group was split into two groups with half the sample size and events each, as per the Cochrane handbook [25].

Whenever we encountered multiple, independent comparisons (e.g. studies with results from an intervention and control group stratified by previous preterm birth or not) we included both of them in the meta-analysis as if they were from different studies. In addition, in our sensitivity analyses we compared our results with the results of an alternative strategy in which the comparisons of the same study are first combined using a fixed-effects model and then the result is pooled with the rest of the studies in a random-effects meta-analysis [25].

### Subgroup and sensitivity analyses

We did subgroup analyses by potential conflicts of interest (including the provision of the probiotic product by the producing company) and length of exposure (lasting up to the end of pregnancy or not). In addition to the planned sensitivity analysis including only studies with low risk of bias we also did further sensitivity analyses excluding studies where the absence of twins was not confirmed, where the comparison group received conventional yogurt, using the alternative method to deal with multiple independent comparisons (involving fixed-effect meta-analysis), and using the correlation of other studies in the meta-analysis to impute the standard deviations of the measures of change. Publication bias was assessed using Duval and Tweedie’s trim-and-fill method when there were at least ten studies in a meta-analysis [27, 28].

## Results

**Fig. 1:**
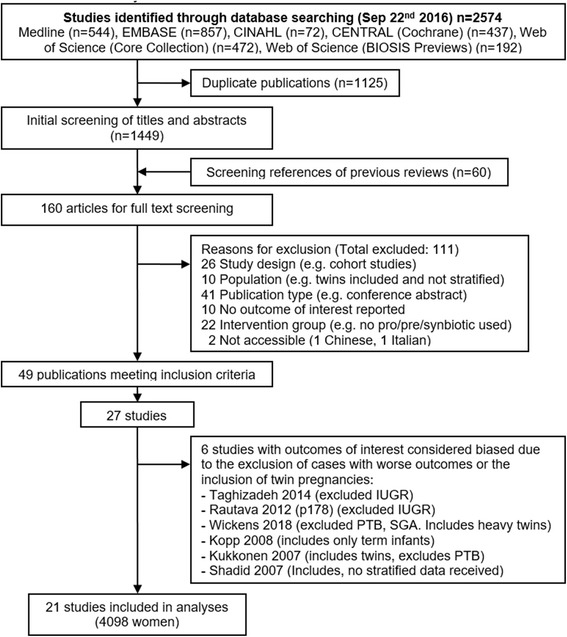
Flow diagram. Flow diagram of study identification and selection in systematic review of the use of probiotics and/or prebiotics during pregnancy

We identified 2574 publications in our search strategy, removed 1125 duplicates and screened 1449 titles and abstracts, as well as reference lists from previous reviews on this and closely related topics (which provided 60 additional references), resulting in 160 articles that we read in full text and assessed for inclusion and exclusion criteria. Of these, 49 publications met our inclusion criteria, but represented only 27 independent studies, as some studies were reported in more than one publication. The most complete publication was generally used, but we also extracted information on outcomes that were reported in other publications if they were not available in the most complete one (Additional file 2: Table S1). In addition, one author referred us to a publication, not indexed in any of the databases used, with further details of their study [29]. Of the 27 identified studies, six excluded cases of preterm birth and intrauterine growth restriction and we considered their infant data not appropriate for our review, as excluding these cases could lead to biased results (e.g. mean birth weight would be higher if preterm cases were excluded) [24, 30–34]. The study comparing a prebiotic with placebo identified in a previous review was excluded for this reason [24]. Studies that excluded preterm infants but reported the number of preterm cases in each group were not excluded, although only the unbiased information (number of preterm births) was used. Similarly, studies that had preterm birth as an exclusion criterion but ended up not excluding any cases for this reason were included. In the end, we included 21 studies in our analyses, comprising of 4098 women (Fig. 1) [23, 35–54].

### Study characteristics

Nineteen studies randomly allocated women to one or more probiotic species or either placebo (18) or treatment as usual (1) and one study compared a prebiotic with placebo. In addition, one study compared a probiotic yogurt with a conventional yogurt [47]. All interventions were administered orally (stated explicitly or assumed by context). None of the included studies assessed a synbiotic. The most frequent target population were women whose infants would be at risk of atopy and/or allergies (9 studies), healthy pregnant women (5 studies) and women with gestational diabetes (4 studies). All studies were from high or upper-middle-income countries. [55] Sample sizes ranged from 60 to 644 pregnant women and the intervention period ranged from 1 to 25 weeks (Table 1, Additional file 3: Figure S1). Compliance with the intervention was not consistently reported throughout the studies, but when it was reported, it was approximately 80%, with no significant differences between groups.

**Table 1:** Characteristics of included studies in systematic review of the use of probiotics and/or prebiotics during pregnancy

Study (Country)	Study population (sample size)	Intervention	Control group	Intervention period	Potential conflicts of interest	Risk of bias
*Prebiotics* vs *placebo*						
Bergmann, 2008 (Germany) [44]	Healthy pregnant women (144)	Group 1: Flavored and acidified milk-based supplement, vitamins, minerals and *Fructo-oligosaccharides* Group 2: Flavored and acidified milk-based supplement, vitamins, minerals and *Fructo-oligosaccharides* and *Docosahexaenoic acid*	Placebo	From 21 to 37 weeks of pregnancy.	Yes	Low
*Probiotics* vs *placebo or treatment as usual*						
Kalliomaki 2001 (Finland) [52]	Women carrying a fetus at risk of atopic disease (159)	*L. rhamnosus* GG	Placebo	2–4 weeks before expected delivery	NR	Low
Abrahamsson 2007 (Sweden) [45]	Women carrying a fetus at risk of allergies (232)	*L. reuteri*	Placebo	From 4 weeks before term until delivery	Yes	Low
Niers, 2009, (Netherlands) [43]	Women carrying a fetus at risk of allergies (156)	*B. animalis* subsp. *lactis* W52 *B. bifidum* W23 *Lc. lactis* W58	Placebo	Last 6 weeks of pregnancy	Yes	Unclear
Laitinen 2009 (Finland) [23]	Healthy pregnant women (256)	Dietary counselling *L. rhamnosus* GG *B. animalis* subsp. *lactis* BB12	Group 1: dietary counselling with placebo. Group 2: placebo only	From mean gestational week of 13.9 ± 1.6 until delivery	Yes^b^	Low
Kim, 2010 (Korea) [42]	Women carrying a fetus at risk of allergies (112)	*L. acidophilus* AD031 *B. bifidum* BGN4 *B. animalis* subsp. *lactis* AD011	Placebo	From 8 weeks before the expected delivery to delivery	NR	Unclear
Allen, 2010 (United Kingdom) [50]	Mostly (91%) women carrying a fetus at risk of atopy (454)	*L. salivarius* CUL61 *L. paracasei* CUL08 *B. animalis* subsp. L*actis* CUL34 *B. bifidum* CUL20	Placebo	Last month of pregnancy	Yes	Low
Dotterund 2010 (Norway)	Healthy pregnant women (415)	(Probiotic milk) *L. rhamnosus* GG *L. acidophilus* LA5 *B. animalis* subsp*. Lactis* Bb-12	Placebo	From 36 weeks of gestation to delivery	Yes	Low
Asemi 2011 (Iran) [47]	Healthy pregnant women (82)	(Probiotic yogurt) *L. delbrueckii* subsp*. bulgaricus* *L. acidophilus* LA5 *B. animalis* subsp. *lactis* BB12 *S. thermophiles*	Conventional yogurt (containing starter cultures of *S. thermophilus* and *L. bulgaricus*)	From 28 to 37 weeks of gestation	Yes^c^	Unclear
Boyle 2011 (Australia) [48]	Women carrying a fetus at risk of allergies (250)	*L. rhamnosus* GG	Placebo	36 weeks gestation until delivery	Yes^a^	Low
Krauss-Silva 2011 (Brazil) [41]	Healthy pregnant women with Nugent scores ≥4 (644)	*L. rhamnosus* GR1 *L. reuteri* RC14	Placebo	Six to twelve weeks (depending on the participant’ s gestational age at the time of enrolment) up until the 24th -26th week of gestation.	Unclear^d^	Low
Rautava, 2012, (Finland) [31]	Women carrying a fetus at risk of allergies (241)	Group 1: Dietary food supplement *L. rhamnosus* LPR *B. longum* BL999 Group 2: Dietary food supplement, *L. paracasei* ST11 *B. longum* BL999	Placebo	Last two months of pregnancy	Yes	Low
Ou, 2012, (Taiwan) [53]	Pregnant women with allergies (191)	*L. rhamnosus* GG	Placebo	From 24 weeks of gestation to delivery	NR	Low
Hantoushzadeh, 2012, (Iran) [40]	Women with symptomatic bacterial vaginosis (310)	(Probiotic yogurt) *L. delbrueckii* subsp*. bulgaricus* *L. acidophilus* *B. animalis* subsp. *lactis* *S. thermophiles*	Orally-administered clindamycin (300 mg twice a day for 1 week)	One week starting at mean week of gestation 36.43 ± 1.32	Unclear^e^	Low
Lindsay 2014 (Ireland) [37]	Obese women (165)	*L. salivarius* UCC118	Placebo	Four weeks starting at 24th week of gestation	Unclear^f^	Low
Dolatkhah, 2015, (Iran) [36]	Women with gestational diabetes mellitus (64)	*L. acidophilus* LA5 *L. delbrueckii* subsp. *bulgaricus* LBY-27 *B. animalis* subsp. *lactis* BB12 *S. thermophiles* STY31	Placebo	Eight weeks, starting between 24 and 28 + 6 weeks of gestation	Yes^g^	Low
Lindsay 2015 (Ireland) [37]	Women with impaired glucose tolerance or gestational diabetes mellitus (149)	*L. salivarius* UCC118	Placebo	From mean week of gestation 31.5 ± 2.2 until delivery	Yes	Low
Mastromarino 2015 (Italy) [54]	Healthy pregnant women (67)	*L. acidophilus* *L. plantarum* *L. paracasei* *L. delbrueckii* subsp. *bulgaricus* *B. longum* *B. breve* *B. infantis* *S. thermophiles*	Placebo	From the 36th week of pregnancy until delivery	No	Low
Karamali, 2016 (Iran) [46]	Women with gestational diabetes mellitus (60)	*L. acidophilus* *L. casei* *B. bifidum*	Placebo	Six weeks starting at 24–28 weeks of gestation	No	Low
Jafarnejad 2016 (Iran) [49]	Women with gestational diabetes mellitus (82)	*L. acidophilus* *L. plantarum* *L. paracasei* *L. delbrueckii* subsp. *bulgaricus* *B. breve* *B. longum* *B. infantis* *S. thermophilus*	Placebo	Eight weeks starting at a mean gestational week 26.4 (SD not reported)	No	Unclear
Ho 2016 (Taiwan) [35]	Group B *Streptococcus*- positive pregnant women (110)	*L. rhamnosus* GR1 *L. reuteri* RC14	Placebo	Intervention lasted a mean of 21.1 ± 5.5 days until delivery.	No	Low

^a^Probiotic and placebo capsules were manufactured and supplied by Dicofarm ltd (Roma, Italy)

^b^Provision of food products was by Raisio plc (Raisio, Finland), B. lactis Bb12 by Chr. Hansen (Hoersholm, Denmark) and L. rhamnosus GG by Valio Ltd. (Helsinki)

^c^The dairy products for the study were provided by the Research and Deevelopmenent Division of Iran Dairy Industry Corporation in Tehran

^d^The authors thank Dr. Gregor Reid for providing the necessary batches of probiotics and placebo capsules used in the trial

^e^Unclear if probiotic yogurt was provided by the producing company (Pegah Company) without cost

^f^Probiotic and placebo capsules were produced and supplied by Alimentary Health Ltd. Unclear if without cost

^g^Tehran Darou Pharmaceuticals, Tehran, Iran, provided the probiotic supplement for the study

### Results by intervention type

**Fig. 2:**
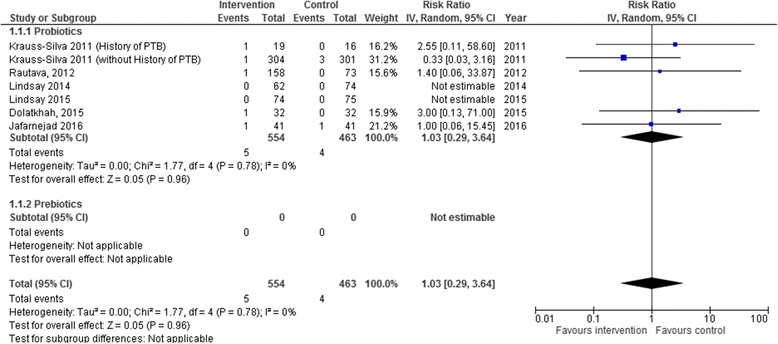
Preterm birth < 34 weeks. Forest plot of the association of probiotics and prebiotics on preterm birth <34 weeks in systematic review of the use of probiotics and/or prebiotics during pregnancy

**Fig. 3:**
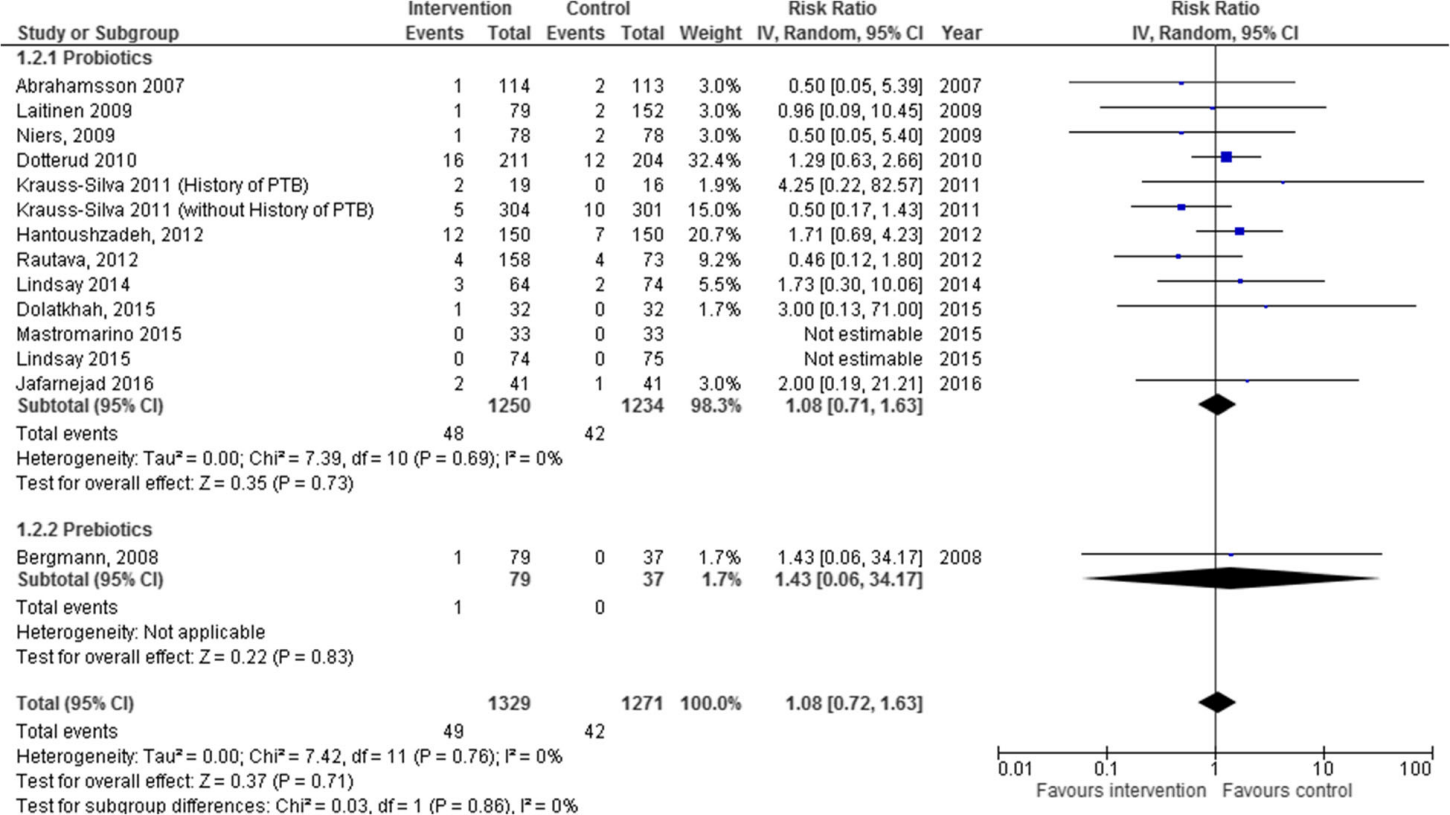
Preterm birth < 37 weeks. Forest plot of the association of probiotics and prebiotics on preterm birth < 37 weeks in systematic review of the use of probiotics and/or prebiotics during pregnancy

Taking probiotics during pregnancy neither increased nor decreased the risk of preterm birth < 34 weeks (RR 1.03, 95% CI 0.29–3.64, I^2^ 0%, 1017 women in 5 studies) or preterm birth < 37 weeks (RR 1.08, 95% CI 0.71–1.63, I^2^ 0%, 2484 women in 11 studies). The one study assessing prebiotics found an increased risk for preterm birth < 37 weeks, although with very wide confidence intervals due to its low power (RR 1.43, 95% CI 0.06–34.17, 116 women in 1 study) (Table 2, Figs. 2 and 3).

**Table 2:** Results by intervention type for primary and key secondary outcomes in systematic review of the use of probiotics and/or prebiotics during pregnancy

Outcome	Intervention	Studies	N	I^2^	RR/MD (95% CI)
PTB < 34	Probiotics	5	1017	0%	RR 1.03 (0.29–3.64)
	Prebiotics	0	–	–	–
PTB < 37	Probiotics	11	2484	0%	RR 1.08 (0.71–1.63)
	Prebiotics	1	116	–	RR 1.43 (0.06–34.17)
Gestational age *(weeks)*	Probiotics	8	1133	0%	MD 0.07 weeks (-0.09–0.23)
	Prebiotics	1	115	–	MD -0.37 weeks (-1.14–0.40)
Birth weight *(grams)*	Probiotics	10	1608	0%	MD 10.66 g (-35.85–57.18)
	Prebiotics	1	116	–	MD -63.95 g (-262.02–134.12)
SGA	Probiotics	3	318	50%	RR 1.03 (0.35–3.06)
	Prebiotics	0	–	–	–
LGA	Probiotics	3	316	0%	RR 0.96 (0.47–1.94)
	Prebiotics	0	–	–	–
GDM	Probiotics	2	355	0%	RR 1.25 (0.61–2.56)
	Prebiotics	0	–	–	–
PPROM	Probiotics	2	366	0%	RR 1.37 (0.63–2.99)
	Prebiotics	0	–	–	–

Abbreviations: *GDM* gestational diabetes mellitus, *LGA* large for gestational age, *N* Number of women in the meta-analysis, *PPROM* Preterm premature rupture of the membranes, *PTB* preterm birth; *SGA* Small for gestational age

Although several individual effect sizes from the studies showed either important benefit or important harm, ranging from relative risks of 0.5 to 4.25, this is likely attributed to the low prevalence of preterm births and the generally small sample sizes, resulting in important deviations from the null effect (relative risk of 1) when there are only small differences in the number of events. However, for the same reason, these results are also accompanied by wide, overlapping confidence intervals, which likely explains the absence of heterogeneity in the meta-analyses, as indicated by I^2^ = 0%.

There was no significant increase or decrease in any of our key secondary outcomes in women receiving either probiotics or prebiotics (Table 2), including small for gestational age infants (RR 1.03, 95% CI 0.35–3.06, I^2^ 50%, 318 women in 3 studies assessing probiotics), large for gestational age infants (RR 0.96, 95% CI 0.47–1.94, I^2^ 0%, 316 women in 3 studies assessing probiotics), gestational diabetes (RR 1.25, 95% CI 0.61–2.56, I^2^ 0%, 355 women in two studies assessing progesterone), and PPROM (RR 1.37, 95% CI 0.63–2.99, I^2^ 0%, 366 women in two studies).

Among the rest of secondary outcomes (Additional file 2: Table S2), the only statistically significant outcomes were continuous outcomes related to glucose metabolism in studies assessing probiotics, with statistically significant differences in HOMA-IR (MD -0.49, 95% CI -0.91– -0.07, I^2^ 79%, 545 women in 6 studies), HOMA-BC (MD -16.90, 95% CI -32.51– -1.29, 60 women in 1 study) and Insulin (MD −2.22 *μ*IU/mL, 95% CI -4.26– -0.18, I^2^ 83%, 496 women in 6 studies).

We reported individual study data for each outcome in Additional file 4: Appendix B.

### Results by genus

Eight studies used one or more species of only *Lactobacillus*, six studies used a combination of *Lactobacillus* and *Bifidobacterium* species, five studies used a combination of *Lactobacillus, Bifidobacterium* and *Streptococcus* species; and one study combined two *Bifidobacterium* species with *Lactococcus lactis*.

When pooling studies in which the probiotics intervention included at least one species of *Lactobacillus* we found similar results as the overall results, with no significant increased or decreased risk of the primary or key secondary outcomes. Interestingly, the pooled estimates indicated increased risks of preterm birth < 34 and < 37 weeks when pooling studies using at least one species of *Bifidobacterium* (RR 1.54 and RR 1.21, respectively) or at least one species of *Streptococcus* (RR 1.60 and RR 1.81, respectively), although with wide, not statistically significant confidence intervals (Table 3).

**Table 3:** Results by genus (study is included if it includes at least one strain of that genus) for primary and key secondary outcomes in systematic review of the use of probiotics and/or prebiotics during pregnancy

Outcome	Intervention	Studies	N	I^2^	RR/MD (95% CI)
PTB < 34	*Lactobacillus* (& other)	5	1017	0%	RR 1.03 (0.29–3.64)
	*Bifidobacterium* (& other)	3	377	0%	RR 1.54 (0.27–8.73)
	*Streptococcus* (& other)	2	146	0%	RR 1.60 (0.20–12.69)
PTB < 37	*Lactobacillus* (& other)	10	2328	0%	RR 1.10 (0.72–1.68)
	*Bifidobacterium* (& other)	7	1479	0%	RR 1.21 (0.75–1.96)
	*Streptococcus* (& other)	3	512	0%	RR 1.81 (0.80–4.10)
Gestational age *(weeks)*	*Lactobacillus* (& other)	8	1133	0%	MD 0.07 weeks (-0.09–0.23)
	*Bifidobacterium* (& other)	3	365	0%	MD 0.30 weeks (0.03–0.57)
	*Streptococcus* (& other)	1	66	–	MD 0.40 weeks (-0.10–0.90)
Birth weight *(grams)*	*Lactobacillus* (& other)	10	1608	0%	MD 10.66 g (-35.85–57.18)
	*Bifidobacterium* (& other)	5	840	0%	MD 22.44 g (-40.58–85.46)
	*Streptococcus* (& other)	1	66	–	MD -65.00 g (-301.73–171.73)
SGA	*Lactobacillus* (& other)	3	318	50%	RR 1.03 (0.35–3.06)
	*Bifidobacterium* (& other)	1	66	–	RR 11.00 (0.63–191.27)
	*Streptococcus* (& other)	1	66	–	RR 11.00 (0.63–191.27)
LGA	*Lactobacillus* (& other)	3	316	0%	RR 0.96 (0.47–1.94)
	*Bifidobacterium* (& other)	1	66	–	RR 0.67 (0.12–3.73)
	*Streptococcus* (& other)	1	66	–	RR 0.67 (0.12–3.73)
GDM	*Lactobacillus* (& other)	2	355	0%	RR 1.25 (0.61–2.56)
	*Bifidobacterium* (& other)	1	219	–	RR 1.26 (0.56–2.83)
	*Streptococcus* (& other)	0	–	–	*Not estimable*
PPROM	*Lactobacillus* (& other)	2	366	0%	RR 1.37 (0.63–2.99)
	*Bifidobacterium* (& other)	2	366	0%	RR 1.37 (0.63–2.99)
	*Streptococcus* (& other)	2	366	0%	RR 1.37 (0.63–2.99)

*Abbreviations:* *GDM* gestational diabetes mellitus, *LGA* large for gestational age, *N*, Number of women in the meta-analysis, *PPROM* Preterm premature rupture of the membranes, *PTB* preterm birth, *SGA* Small for gestational age

### Results by species

Pooling separately different species of probiotics and their combinations resulted in subgroups with only one study in most of the subgroups, as almost each study used a different species or combination of probiotics. None of these subgroups reached statistical significance. We reported the results for primary outcomes pooling separately different species of probiotics in Additional file 3: Figures S2 and S3.

### Subgroup and sensitivity analyses

We considered 11 studies to potentially have a conflict of interest (either explicitly declared or by receiving the intervention products from the manufacturer without any cost), 4 studies to have no conflict of interest, and 6 studies in which it was unclear if there was a conflict of interest (either because it was not reported or because the relationship with the product providers was unclear). In eight studies the intervention was given during pregnancy but not until delivery (Table 1).

We found no statistically significant differences between studies reporting no conflicts of interest and studies with potential conflicts of interest (Additional file 2: Table S3). Similarly, we found no significant differences between studies in which the exposure to probiotics lasted up to the end of the pregnancy or not (Additional file 2: Table S4).

We considered 17 studies to have an overall low risk of bias. All our sensitivity analyses yielded very similar results, if not identical, as the original analyses (Additional file 2: Tables S5 to S8). We did not detect publication bias in any of the meta-analyses.

## Discussion

### Main findings

Overall, we found no evidence of either harm or benefit of probiotics or prebiotics on preterm birth or other adverse infant and maternal clinical outcomes. Furthermore, although most of the outcomes were underpowered and hence the confidence intervals were wide and not statistically significant, the point estimates were generally around the null, suggesting no difference between intervention and control.

### Interpretation in the context of the literature

To our knowledge, the most recent systematic review reporting the risks of probiotics for preterm birth was the Cochrane review on gestational diabetes, published in 2014 (literature search in 2013), while the Cochrane review that focused on the association of probiotics and preterm birth was updated in 2010 (literature search in 2010) [19, 22]. Both meta-analyses included only one (the same) study, obtaining relative risks between 3 and 4 (albeit not statistically significant), in contrast to our results. This difference can be explained not only by our inclusion of 11 additional studies, but also by our exclusion of twin data, which is expected to be biased due to their worse outcomes. In fact, for the study included in the previous reviews, after obtaining the data for singletons alone from the study authors we found that the relative risk was actually 0.96 (95% CI 0.09–10.45). This emphasizes the importance of taking into account potential confounding factors in the design of studies of pregnant women to assess outcomes on them and their foetuses, as including twin pregnancies and/or excluding the less healthy infants can introduce considerable biases.

Similarly, in a previous Cochrane review, gestational diabetes was found to be significantly reduced in one study (RR 0.38, 95% CI 0.20–0.70), but it is unclear in how far this result was influenced by twin pregnancies. We could not obtain the data for singletons only for this outcome for this study, but the pooled estimate on gestational diabetes of three other studies does not show any benefit from probiotics on this outcome (RR 1.25, 95% CI 0.61–2.56).

Previous literature has also shown that, in many cases, there is a lack of correlation between the label and the actual content of probiotic products [56] and that the original properties of specific probiotic strains can be affected by the industrial production processes, which could lead to commercial probiotic products not preserving the intended original properties [57]. Therefore, it is not possible to extrapolate our results, which are based on highly controlled trials, to the potential effect of commercial probiotic products in the general population.

There are currently several trials registered in clinicaltrials.gov that explicitly assess the potential effect of probiotics on preterm birth. Unless their results are markedly different, however, we do not expect that our conclusions would have to be changed.

### Strengths and limitations

The main strength of this systematic review was the relatively high number of studies identified. While previous systematic reviews had found only one study, we managed to pool data from 12 studies on preterm birth, including four studies whose authors provided us with stratified and/or unreported information. Furthermore, another important strength of our review was the careful consideration of inclusion and exclusion criteria to avoid biases introduced by the presence of twin pregnancies or the exclusion of infants with worse outcomes (i.e. preterm birth and intrauterine growth restriction). Such caution was justified by the fact that many studies limited their results to term pregnancies only, in which cases we contacted the authors to try to obtain data from the whole sample of pregnant women, regardless of the pregnancy outcome. Another strength was our analysis at different levels, pooling probiotics and prebiotics separately first, then grouping studies by probiotic type (genus), and finally reporting the results by separate species. Given the lack of consensus in the field regarding the most appropriate way of grouping (or not) the different interventions, we hope that by being transparent and reporting the results in detail each point of view might benefit from our review.

The main limitation of our review was the low number of primary randomized controlled studies assessing prebiotics and synbiotics, which limits the extent of our results to probiotic interventions only. Another limitation of our review was that only one study focused on the prevention of preterm birth, while the rest of the studies focused on other outcomes, such as maternal glucose metabolism and allergies in the infants, with preterm birth reported only as a secondary outcome or a baseline characteristic. This also explains the considerable variation in the timing and duration of the probiotic administration. Furthermore, the fact that an important number of studies were aimed at reducing allergic disease in offspring could be a reason for the scarce amount of data on maternal and delivery related outcomes. However, one potential benefit of this is that it could have minimised the risk of publication bias or the potential effect of conflicts of interest. Another limitation is the primary studies’ generally small size, which meant that even when we pooled data, many of our outcomes lacked sufficient statistical power, as evidenced by the wide confidence intervals, which limits somewhat the robustness of our conclusions. However, the absence of heterogeneity, as well as the fact that the effect estimates are close to the null effect in most of our analyses, show a relatively consistent picture.

Another limitation of our study is the timing and duration of the probiotic administration, which ranged from one to 26 weeks. Although in most of the studies the intervention took place during the third trimester, some of the studies included the probiotics quite early in the pregnancy and some included them only in the last weeks of the pregnancy. It is therefore possible that the length of exposure could affect our findings. However, given the small amount of heterogeneity detected in our meta-analyses we did not consider appropriate to extend the subgroup analyses beyond what was planned.

Finally, random-effects meta-analyses with a small number of studies remains a challenging scenario and there is no clear guidance on how to best proceed [58]. However, this would only apply to the few analyses in which there was heterogeneity between studies (I^2^ > 0), as doing a random-effects meta-analysis in which there is no heterogeneity is equivalent to a fixed-effects model.

## Conclusions

More randomized studies are required that assess the safety or efficacy of taking prebiotics during pregnancy. Pooling the existing studies, we found no evidence that taking probiotics during pregnancy either increases or decreases the risk of preterm birth or other infant and maternal adverse pregnancy outcomes. However, more homogeneous studies in terms of type of probiotics used, length of exposure and women’s characteristics are needed. It is important to note that these results might not apply outside the context of randomized research.

## Additional files

Additional file 1: Appendix A. Complete search strategy. Complete search strategy; Search terms used in each of the databases used. (DOC 308 kb)

Additional file 2: Tables S1–S8. Tables with additional information and details. (DOCX 43 kb)

Additional file 3: Figure S1–S3. Additional figures of interest. (DOCX 109 kb)

Additional file 4: Appendix B. Individual study data.docx; Individual study data; Individual study data for all outcomes, comparisons and analyses. (DOCX 1588 kb)

## Abbreviations

95% CI: 95% Confidence Interval
GDM: Gestational diabetes
HOMA-BC: Homeostatic Model Assessment of Beta-cell function
HOMA-IR: Homeostatic Model Assessment of Insulin Resistance
MD: Mean Difference
NICU: Neonatal Intensive Care Unit
PPROM: Premature Preterm Rupture of Membranes
RR: Risk Ratio
