# Supplementary material for: Pregnancy outcomes in women taking probiotics or prebiotics: a systematic review and meta-analysis
Source: BMC Pregnancy Childbirth. 2018 Jan 8;18:14. doi: 10.1186/s12884-017-1629-5 (PMC5759212; doi:10.1186/s12884-017-1629-5)
Supplement: Supplementary file 1 — Complete search strategy. Complete search strategy; Search terms used in each of the databases used. (DOC 308 kb) [file 12884_2017_1629_MOESM1_ESM.doc]

**Additional file 1**

**Appendix A: Complete search strategy**

Executed on September 22, 2016 with no language restrictions

# MEDLINE

| 1 | pregnancy/ | 789675 |
| --- | --- | --- |
| 2 | pregnan:.mp. | 885345 |
| 3 | pregnancy complications/ | 81683 |
| 4 | 1 or 2 or 3 | 885345 |
| 5 | probiotics/ | 11495 |
| 6 | probio:.mp. | 17004 |
| 7 | lactobacillus/ | 14676 |
| 8 | lactobacill:.mp. | 32222 |
| 9 | bifidobacterium/ | 4636 |
| 10 | bifidobacteri:.mp. | 7553 |
| 11 | clausii:.mp. | 99 |
| 12 | streptococcus thermophilus/ | 598 |
| 13 | (streptococc: adj4 thermophil:).mp. | 1586 |
| 14 | boulardii:.mp. | 553 |
| 15 | freudenreichii:.mp. | 332 |
| 16 | salivarius:.mp. | 1920 |
| 17 | escherichia coli/ | 242397 |
| 18 | (escherichia: adj4 coli:).mp. | 344187 |
| 19 | enterococcus faecium/ | 3001 |
| 20 | (enterococc: adj4 faecium:).mp. | 5172 |
| 21 | 5 or 6 or 7 or 8 or 9 or 10 or 11 or 12 or 13 or 14 or 15 or 16 or 17 or 18 or 19 or 20 | 390095 |
| 22 | prebiotics/ | 1349 |
| 23 | prebio:.mp. | 5768 |
| 24 | inulin/ | 6418 |
| 25 | inulin:.mp. | 10580 |
| 26 | fructans/ | 860 |
| 27 | fructan:.mp. | 1450 |
| 28 | oligofructose:.mp. | 438 |
| 29 | fructooligosaccharide:.mp. | 841 |
| 30 | fructo-oligosaccharide:.mp. | 484 |
| 31 | galactooligosaccharide:.mp. | 332 |
| 32 | galacto-oligosaccharide:.mp. | 398 |
| 33 | transgalactooligosaccharide:.mp. | 12 |
| 34 | trans-galactooligosaccharide:.mp. | 9 |
| 35 | lactulose/ | 1906 |
| 36 | lactulos:.mp. | 3369 |
| 37 | 22 or 23 or 24 or 25 or 26 or 27 or 28 or 29 or 30 or 31 or 32 or 33 or 34 or 35 or 36 | 20817 |
| 38 | synbiotics/ | 237 |
| 39 | synbio:.mp. | 781 |
| 40 | 38 or 39 | 781 |
| 41 | 21 or 37 or 40 | 407491 |
| 42 | randomized controlled trial/ or (trial* or random* or RCT*).mp. | 1797496 |
| 43 | (double: adj4 blind:).mp. | 173831 |
| 44 | (triple: adj4 blind:).mp. | 594 |
| 45 | 42 or 43 or 44 | 1805630 |
| **46** | **4 and 41 and 45** | **544** |

# EMBASE

| 1 | pregnancy/ | 668059 |
| --- | --- | --- |
| 2 | pregnan:.mp. | 869109 |
| 3 | pregnancy complication/ | 71778 |
| 4 | 1 or 2 or 3 | 869109 |
| 5 | probiotic agent/ | 25084 |
| 6 | probio:.mp. | 27197 |
| 7 | Lactobacillus/ | 19726 |
| 8 | lactobacill:.mp. | 39074 |
| 9 | Bifidobacterium/ | 5970 |
| 10 | bifidobacteri:.mp. | 11864 |
| 11 | clausii:.mp. | 153 |
| 12 | Streptococcus thermophilus/ | 2052 |
| 13 | (streptococc: adj4 thermophil:).mp. | 2492 |
| 14 | boulardii:.mp. | 1443 |
| 15 | freudenreichii:.mp. | 719 |
| 16 | salivarius:.mp. | 2873 |
| 17 | enterococcus faecium/ | 6557 |
| 18 | (enterococc: adj4 faecium:).mp. | 7865 |
| 19 | Escherichia coli/ | 322085 |
| 20 | (escherichia: adj4 coli:).mp. | 383403 |
| 21 | 5 or 6 or 7 or 8 or 9 or 10 or 11 or 12 or 13 or 14 or 15 or 16 or 17 or 18 or 19 or 20 | 441998 |
| 22 | prebiotic agent/ | 5460 |
| 23 | prebio:.mp. | 8457 |
| 24 | inulin/ | 8232 |
| 25 | inulin:.mp. | 13930 |
| 26 | fructan/ | 1223 |
| 27 | fructan:.mp. | 1608 |
| 28 | oligofructose.mp. | 562 |
| 29 | fructooligosaccharide:.mp. | 1006 |
| 30 | fructo-oligosaccharide:.mp. | 639 |
| 31 | galactooligosaccharide:.mp. | 462 |
| 32 | galacto-oligosaccharide:.mp. | 541 |
| 33 | transgalactooligosaccharide:.mp. | 14 |
| 34 | trans-galactooligosaccharide:.mp. | 9 |
| 35 | lactulose/ | 7343 |
| 36 | lactulos:.mp. | 8224 |
| 37 | 22 or 23 or 24 or 25 or 26 or 27 or 28 or 29 or 30 or 31 or 32 or 33 or 34 or 35 or 36 | 31255 |
| 38 | synbiotic agent/ | 968 |
| 39 | synbio:.mp. | 1351 |
| 40 | 38 or 39 | 1351 |
| 41 | 21 or 37 or 40 | 467456 |
| 42 | randomized controlled trial/ or (trial* or random* or RCT*).mp. | 2398912 |
| 43 | (double: adj4 blind:).mp. | 213345 |
| 44 | (triple: adj4 blind:).mp. | 797 |
| 45 | 42 or 43 or 44 | 2419827 |
| **46** | **4 and 41 and 45** | **857** |

# WoS

| # 34 | [**472**](http://apps.webofknowledge.com/summary.do?product=WOS&doc=1&qid=34&SID=4C58vIhBILL3xUZlivy&search_mode=CombineSearches&update_back2search_link_param=yes) | **#33 AND #26 AND #1** |
| --- | --- | --- |
| # 33 | [2,149,657](http://apps.webofknowledge.com/summary.do?product=WOS&doc=1&qid=33&SID=4C58vIhBILL3xUZlivy&search_mode=CombineSearches&update_back2search_link_param=yes) | #32 OR #31 OR #30 OR #29 OR #28 OR #27 |
| # 32 | [489](http://apps.webofknowledge.com/summary.do?product=WOS&doc=1&qid=32&SID=4C58vIhBILL3xUZlivy&search_mode=AdvancedSearch&update_back2search_link_param=yes) | TS= (triple* NEAR/4 blind*) |
| # 31 | [200,024](http://apps.webofknowledge.com/summary.do?product=WOS&doc=1&qid=31&SID=4C58vIhBILL3xUZlivy&search_mode=AdvancedSearch&update_back2search_link_param=yes) | TS= (double* NEAR/4 blind*) |
| # 30 | [25,350](http://apps.webofknowledge.com/summary.do?product=WOS&doc=1&qid=30&SID=4C58vIhBILL3xUZlivy&search_mode=AdvancedSearch&update_back2search_link_param=yes) | TS= RCT* |
| # 29 | [1,390,464](http://apps.webofknowledge.com/summary.do?product=WOS&doc=1&qid=29&SID=4C58vIhBILL3xUZlivy&search_mode=AdvancedSearch&update_back2search_link_param=yes) | TS= random* |
| # 28 | [1,236,556](http://apps.webofknowledge.com/summary.do?product=WOS&doc=1&qid=28&SID=4C58vIhBILL3xUZlivy&search_mode=AdvancedSearch&update_back2search_link_param=yes) | TS= trial* |
| # 27 | [321,844](http://apps.webofknowledge.com/summary.do?product=WOS&doc=1&qid=27&SID=4C58vIhBILL3xUZlivy&search_mode=AdvancedSearch&update_back2search_link_param=yes) | TS= randomized controlled trial* |
| # 26 | [455,866](http://apps.webofknowledge.com/summary.do?product=WOS&doc=1&qid=26&SID=4C58vIhBILL3xUZlivy&search_mode=CombineSearches&update_back2search_link_param=yes) | #25 OR #24 OR #12 |
| # 25 | [1,131](http://apps.webofknowledge.com/summary.do?product=WOS&doc=1&qid=25&SID=4C58vIhBILL3xUZlivy&search_mode=AdvancedSearch&update_back2search_link_param=yes) | TS= synbio* |
| # 24 | [21,896](http://apps.webofknowledge.com/summary.do?product=WOS&doc=1&qid=24&SID=4C58vIhBILL3xUZlivy&search_mode=CombineSearches&update_back2search_link_param=yes) | #23 OR #22 OR #21 OR #20 OR #19 OR #18 OR #17 OR #16 OR #15 OR #14 OR #13 |
| # 23 | [3,445](http://apps.webofknowledge.com/summary.do?product=WOS&doc=1&qid=23&SID=4C58vIhBILL3xUZlivy&search_mode=AdvancedSearch&update_back2search_link_param=yes) | TS= lactulos* |
| # 22 | [15](http://apps.webofknowledge.com/summary.do?product=WOS&doc=1&qid=22&SID=4C58vIhBILL3xUZlivy&search_mode=AdvancedSearch&update_back2search_link_param=yes) | TS= trans-galactooligosaccharide* |
| # 21 | [17](http://apps.webofknowledge.com/summary.do?product=WOS&doc=1&qid=21&SID=4C58vIhBILL3xUZlivy&search_mode=AdvancedSearch&update_back2search_link_param=yes) | TS= transgalactooligosaccharide* |
| # 20 | [751](http://apps.webofknowledge.com/summary.do?product=WOS&doc=1&qid=20&SID=4C58vIhBILL3xUZlivy&search_mode=AdvancedSearch&update_back2search_link_param=yes) | TS= galacto-oligosaccharide* |
| # 19 | [621](http://apps.webofknowledge.com/summary.do?product=WOS&doc=1&qid=19&SID=4C58vIhBILL3xUZlivy&search_mode=AdvancedSearch&update_back2search_link_param=yes) | TS= galactooligosaccharide* |
| # 18 | [1,190](http://apps.webofknowledge.com/summary.do?product=WOS&doc=1&qid=18&SID=4C58vIhBILL3xUZlivy&search_mode=AdvancedSearch&update_back2search_link_param=yes) | TS= fructo-oligosaccharide* |
| # 17 | [1,722](http://apps.webofknowledge.com/summary.do?product=WOS&doc=1&qid=17&SID=4C58vIhBILL3xUZlivy&search_mode=AdvancedSearch&update_back2search_link_param=yes) | TS= fructooligosaccharide* |
| # 16 | [1,392](http://apps.webofknowledge.com/summary.do?product=WOS&doc=1&qid=16&SID=4C58vIhBILL3xUZlivy&search_mode=AdvancedSearch&update_back2search_link_param=yes) | TS= oligofructose* |
| # 15 | [2,467](http://apps.webofknowledge.com/summary.do?product=WOS&doc=1&qid=15&SID=4C58vIhBILL3xUZlivy&search_mode=AdvancedSearch&update_back2search_link_param=yes) | TS= fructan* |
| # 14 | [7,704](http://apps.webofknowledge.com/summary.do?product=WOS&doc=1&qid=14&SID=4C58vIhBILL3xUZlivy&search_mode=AdvancedSearch&update_back2search_link_param=yes) | TS= inulin* |
| # 13 | [9,124](http://apps.webofknowledge.com/summary.do?product=WOS&doc=1&qid=13&SID=4C58vIhBILL3xUZlivy&search_mode=AdvancedSearch&update_back2search_link_param=yes) | TS= prebio* |
| # 12 | [438,995](http://apps.webofknowledge.com/summary.do?product=WOS&doc=1&qid=12&SID=4C58vIhBILL3xUZlivy&search_mode=CombineSearches&update_back2search_link_param=yes) | #11 OR #10 OR #9 OR #8 OR #7 OR #6 OR #5 OR #4 OR #3 OR #2 |
| # 11 | [6,165](http://apps.webofknowledge.com/summary.do?product=WOS&doc=1&qid=11&SID=4C58vIhBILL3xUZlivy&search_mode=AdvancedSearch&update_back2search_link_param=yes) | TS= (enterococc* NEAR/4 faecium*) |
| # 10 | [387,641](http://apps.webofknowledge.com/summary.do?product=WOS&doc=1&qid=10&SID=4C58vIhBILL3xUZlivy&search_mode=AdvancedSearch&update_back2search_link_param=yes) | TS= (escherichia* NEAR/4 coli*) |
| # 9 | [2,079](http://apps.webofknowledge.com/summary.do?product=WOS&doc=1&qid=9&SID=4C58vIhBILL3xUZlivy&search_mode=AdvancedSearch&update_back2search_link_param=yes) | TS= salivarius* |
| # 8 | [644](http://apps.webofknowledge.com/summary.do?product=WOS&doc=1&qid=8&SID=4C58vIhBILL3xUZlivy&search_mode=AdvancedSearch&update_back2search_link_param=yes) | TS= freudenreichii* |
| # 7 | [1,303](http://apps.webofknowledge.com/summary.do?product=WOS&doc=1&qid=7&SID=4C58vIhBILL3xUZlivy&search_mode=AdvancedSearch&update_back2search_link_param=yes) | TS= boulardii* |
| # 6 | [3,416](http://apps.webofknowledge.com/summary.do?product=WOS&doc=1&qid=6&SID=4C58vIhBILL3xUZlivy&search_mode=AdvancedSearch&update_back2search_link_param=yes) | TS= (streptococc* NEAR/4 thermophil*) |
| # 5 | [178](http://apps.webofknowledge.com/summary.do?product=WOS&doc=1&qid=5&SID=4C58vIhBILL3xUZlivy&search_mode=AdvancedSearch&update_back2search_link_param=yes) | TS= clausii* |
| # 4 | [10,270](http://apps.webofknowledge.com/summary.do?product=WOS&doc=1&qid=4&SID=4C58vIhBILL3xUZlivy&search_mode=AdvancedSearch&update_back2search_link_param=yes) | TS= bifidobacteri* |
| # 3 | [38,442](http://apps.webofknowledge.com/summary.do?product=WOS&doc=1&qid=3&SID=4C58vIhBILL3xUZlivy&search_mode=AdvancedSearch&update_back2search_link_param=yes) | TS= lactobacill* |
| # 2 | [22,036](http://apps.webofknowledge.com/summary.do?product=WOS&doc=1&qid=2&SID=4C58vIhBILL3xUZlivy&search_mode=AdvancedSearch&update_back2search_link_param=yes) | TS= probio* |
| # 1 | [374,347](http://apps.webofknowledge.com/summary.do?product=WOS&doc=1&qid=1&SID=4C58vIhBILL3xUZlivy&search_mode=AdvancedSearch&update_back2search_link_param=yes) | TS= pregnan* |

# BIOSIS Previews

| **# 34** | [**192**](http://apps.webofknowledge.com/summary.do?product=BIOSIS&doc=1&qid=68&SID=4C58vIhBILL3xUZlivy&search_mode=AdvancedSearch&update_back2search_link_param=yes) | **#33 AND #26 AND #1** |
| --- | --- | --- |
| # 33 | [1,050,566](http://apps.webofknowledge.com/summary.do?product=BIOSIS&doc=1&qid=67&SID=4C58vIhBILL3xUZlivy&search_mode=AdvancedSearch&update_back2search_link_param=yes) | #32 OR #31 OR #30 OR #29 OR #28 OR #27 |
| # 32 | [318](http://apps.webofknowledge.com/summary.do?product=BIOSIS&doc=1&qid=66&SID=4C58vIhBILL3xUZlivy&search_mode=AdvancedSearch&update_back2search_link_param=yes) | TS= (triple* NEAR/4 blind*) |
| # 31 | [94,802](http://apps.webofknowledge.com/summary.do?product=BIOSIS&doc=1&qid=65&SID=4C58vIhBILL3xUZlivy&search_mode=AdvancedSearch&update_back2search_link_param=yes) | TS= (double* NEAR/4 blind*) |
| # 30 | [9,879](http://apps.webofknowledge.com/summary.do?product=BIOSIS&doc=1&qid=64&SID=4C58vIhBILL3xUZlivy&search_mode=AdvancedSearch&update_back2search_link_param=yes) | TS= RCT* |
| # 29 | [600,483](http://apps.webofknowledge.com/summary.do?product=BIOSIS&doc=1&qid=63&SID=4C58vIhBILL3xUZlivy&search_mode=AdvancedSearch&update_back2search_link_param=yes) | TS= random* |
| # 28 | [618,003](http://apps.webofknowledge.com/summary.do?product=BIOSIS&doc=1&qid=62&SID=4C58vIhBILL3xUZlivy&search_mode=AdvancedSearch&update_back2search_link_param=yes) | TS= trial* |
| # 27 | [103,976](http://apps.webofknowledge.com/summary.do?product=BIOSIS&doc=1&qid=61&SID=4C58vIhBILL3xUZlivy&search_mode=AdvancedSearch&update_back2search_link_param=yes) | TS= randomized controlled trial* |
| # 26 | [524,198](http://apps.webofknowledge.com/summary.do?product=BIOSIS&doc=1&qid=60&SID=4C58vIhBILL3xUZlivy&search_mode=AdvancedSearch&update_back2search_link_param=yes) | #25 OR #24 OR #12 |
| # 25 | [797](http://apps.webofknowledge.com/summary.do?product=BIOSIS&doc=1&qid=59&SID=4C58vIhBILL3xUZlivy&search_mode=AdvancedSearch&update_back2search_link_param=yes) | TS= synbio* |
| # 24 | [24,653](http://apps.webofknowledge.com/summary.do?product=BIOSIS&doc=1&qid=58&SID=4C58vIhBILL3xUZlivy&search_mode=AdvancedSearch&update_back2search_link_param=yes) | #23 OR #22 OR #21 OR #20 OR #19 OR #18 OR #17 OR #16 OR #15 OR #14 OR #13 |
| # 23 | [3,621](http://apps.webofknowledge.com/summary.do?product=BIOSIS&doc=1&qid=57&SID=4C58vIhBILL3xUZlivy&search_mode=AdvancedSearch&update_back2search_link_param=yes) | TS= lactulos* |
| # 22 | [14](http://apps.webofknowledge.com/summary.do?product=BIOSIS&doc=1&qid=56&SID=4C58vIhBILL3xUZlivy&search_mode=AdvancedSearch&update_back2search_link_param=yes) | TS= trans-galactooligosaccharide* |
| # 21 | [20](http://apps.webofknowledge.com/summary.do?product=BIOSIS&doc=1&qid=55&SID=4C58vIhBILL3xUZlivy&search_mode=AdvancedSearch&update_back2search_link_param=yes) | TS= transgalactooligosaccharide* |
| # 20 | [562](http://apps.webofknowledge.com/summary.do?product=BIOSIS&doc=1&qid=54&SID=4C58vIhBILL3xUZlivy&search_mode=AdvancedSearch&update_back2search_link_param=yes) | TS= galacto-oligosaccharide* |
| # 19 | [552](http://apps.webofknowledge.com/summary.do?product=BIOSIS&doc=1&qid=53&SID=4C58vIhBILL3xUZlivy&search_mode=AdvancedSearch&update_back2search_link_param=yes) | TS= galactooligosaccharide* |
| # 18 | [720](http://apps.webofknowledge.com/summary.do?product=BIOSIS&doc=1&qid=52&SID=4C58vIhBILL3xUZlivy&search_mode=AdvancedSearch&update_back2search_link_param=yes) | TS= fructo-oligosaccharide* |
| # 17 | [1,293](http://apps.webofknowledge.com/summary.do?product=BIOSIS&doc=1&qid=51&SID=4C58vIhBILL3xUZlivy&search_mode=AdvancedSearch&update_back2search_link_param=yes) | TS= fructooligosaccharide* |
| # 16 | [581](http://apps.webofknowledge.com/summary.do?product=BIOSIS&doc=1&qid=50&SID=4C58vIhBILL3xUZlivy&search_mode=AdvancedSearch&update_back2search_link_param=yes) | TS= oligofructose* |
| # 15 | [2,207](http://apps.webofknowledge.com/summary.do?product=BIOSIS&doc=1&qid=49&SID=4C58vIhBILL3xUZlivy&search_mode=AdvancedSearch&update_back2search_link_param=yes) | TS= fructan* |
| # 14 | [12,090](http://apps.webofknowledge.com/summary.do?product=BIOSIS&doc=1&qid=48&SID=4C58vIhBILL3xUZlivy&search_mode=AdvancedSearch&update_back2search_link_param=yes) | TS= inulin* |
| # 13 | [6,785](http://apps.webofknowledge.com/summary.do?product=BIOSIS&doc=1&qid=47&SID=4C58vIhBILL3xUZlivy&search_mode=AdvancedSearch&update_back2search_link_param=yes) | TS= prebio* |
| # 12 | [503,483](http://apps.webofknowledge.com/summary.do?product=BIOSIS&doc=1&qid=46&SID=4C58vIhBILL3xUZlivy&search_mode=AdvancedSearch&update_back2search_link_param=yes) | #11 OR #10 OR #9 OR #8 OR #7 OR #6 OR #5 OR #4 OR #3 OR #2 |
| # 11 | [8,324](http://apps.webofknowledge.com/summary.do?product=BIOSIS&doc=1&qid=45&SID=4C58vIhBILL3xUZlivy&search_mode=AdvancedSearch&update_back2search_link_param=yes) | TS= (enterococc* NEAR/4 faecium*) |
| # 10 | [444,563](http://apps.webofknowledge.com/summary.do?product=BIOSIS&doc=1&qid=44&SID=4C58vIhBILL3xUZlivy&search_mode=AdvancedSearch&update_back2search_link_param=yes) | TS= (escherichia* NEAR/4 coli*) |
| # 9 | [3,321](http://apps.webofknowledge.com/summary.do?product=BIOSIS&doc=1&qid=43&SID=4C58vIhBILL3xUZlivy&search_mode=AdvancedSearch&update_back2search_link_param=yes) | TS= salivarius* |
| # 8 | [767](http://apps.webofknowledge.com/summary.do?product=BIOSIS&doc=1&qid=42&SID=4C58vIhBILL3xUZlivy&search_mode=AdvancedSearch&update_back2search_link_param=yes) | TS= freudenreichii* |
| # 7 | [760](http://apps.webofknowledge.com/summary.do?product=BIOSIS&doc=1&qid=41&SID=4C58vIhBILL3xUZlivy&search_mode=AdvancedSearch&update_back2search_link_param=yes) | TS= boulardii* |
| # 6 | [3,882](http://apps.webofknowledge.com/summary.do?product=BIOSIS&doc=1&qid=40&SID=4C58vIhBILL3xUZlivy&search_mode=AdvancedSearch&update_back2search_link_param=yes) | TS= (streptococc* NEAR/4 thermophil*) |
| # 5 | [254](http://apps.webofknowledge.com/summary.do?product=BIOSIS&doc=1&qid=39&SID=4C58vIhBILL3xUZlivy&search_mode=AdvancedSearch&update_back2search_link_param=yes) | TS= clausii* |
| # 4 | [10,858](http://apps.webofknowledge.com/summary.do?product=BIOSIS&doc=1&qid=38&SID=4C58vIhBILL3xUZlivy&search_mode=AdvancedSearch&update_back2search_link_param=yes) | TS= bifidobacteri* |
| # 3 | [44,137](http://apps.webofknowledge.com/summary.do?product=BIOSIS&doc=1&qid=37&SID=4C58vIhBILL3xUZlivy&search_mode=AdvancedSearch&update_back2search_link_param=yes) | TS= lactobacill* |
| # 2 | [17,658](http://apps.webofknowledge.com/summary.do?product=BIOSIS&doc=1&qid=36&SID=4C58vIhBILL3xUZlivy&search_mode=AdvancedSearch&update_back2search_link_param=yes) | TS= probio* |
| # 1 | [329,840](http://apps.webofknowledge.com/summary.do?product=BIOSIS&doc=1&qid=35&SID=4C58vIhBILL3xUZlivy&search_mode=AdvancedSearch&update_back2search_link_param=yes) | TS= pregnan* |

# CENTRAL

| #1 | MeSH descriptor: [Pregnancy] explode all trees | 6428 |
| --- | --- | --- |
| #2 | pregnan* | 34835 |
| #3 | MeSH descriptor: [Pregnancy Complications] explode all trees | 8800 |
| #4 | #1 or #2 or #3 | 35587 |
| #5 | MeSH descriptor: [Probiotics] explode all trees | 1497 |
| #6 | probio* | 2871 |
| #7 | MeSH descriptor: [Lactobacillus] explode all trees | 1231 |
| #8 | lactobacill* | 2503 |
| #9 | MeSH descriptor: [Bifidobacterium] explode all trees | 503 |
| #10 | bifidobacteri* | 1249 |
| #11 | clausii* | 13 |
| #12 | MeSH descriptor: [Streptococcus thermophilus] explode all trees | 44 |
| #13 | streptococc* near/4 thermophil* | 168 |
| #14 | boulardii* | 171 |
| #15 | freudenreichii* | 29 |
| #16 | salivarius* | 124 |
| #17 | MeSH descriptor: [Enterococcus faecium] explode all trees | 27 |
| #18 | enterococc* near/4 faecium* | 92 |
| #19 | MeSH descriptor: [Escherichia coli] explode all trees | 596 |
| #20 | escherichia* near/4 coli* | 2246 |
| #21 | #5 or #6 or #7 or #8 or #9 or #10 or #11 or #12 or #13 or #14 or #15 or #16 or #17 or #18 or #19 or #20 | 6233 |
| #22 | MeSH descriptor: [Prebiotics] explode all trees | 164 |
| #23 | prebio* | 651 |
| #24 | MeSH descriptor: [Inulin] explode all trees | 164 |
| #25 | inulin* | 596 |
| #26 | MeSH descriptor: [Fructans] explode all trees | 173 |
| #27 | fructan* | 51 |
| #28 | oligofructose* | 89 |
| #29 | fructooligosaccharide* | 101 |
| #30 | fructo-oligosaccharide* | 132 |
| #31 | galactooligosaccharide* | 54 |
| #32 | galacto-oligosaccharide* | 92 |
| #33 | transgalactooligosaccharide* | 2 |
| #34 | trans-galactooligosaccharide* | 4 |
| #35 | MeSH descriptor: [Lactulose] explode all trees | 325 |
| #36 | lactulos* | 980 |
| #37 | #22 or #23 or #24 or #25 or #26 or #27 or #28 or #29 or #30 or #31 or #32 or #33 or #34 or #35 or #36 | 2247 |
| #38 | MeSH descriptor: [Synbiotics] explode all trees | 70 |
| #39 | synbio* | 262 |
| #40 | #38 or #39 | 262 |
| #41 | #21 or #37 or #40 | 7884 |
| #42 | MeSH descriptor: [Randomized Controlled Trial] explode all trees | 157 |
| #43 | trial* | 1011846 |
| #44 | random* | 660256 |
| #45 | RCT* | 243894 |
| #46 | double* near/4 blind* | 202599 |
| #47 | triple* near/4 blind* | 1578 |
| #48 | #42 or #43 or #44 or #45 or #46 or #47 | 1015496 |
| **#49** | **#4 and #41 and #48** | **437** |

# CINAHL

| **S43** | **S4 AND S35 AND S42** | **72** |
| --- | --- | --- |
| S42 | S36 OR S37 OR S38 OR S39 OR S40 OR S41 | 264,019 |
| S41 | triple* n4 blind* | 128 |
| S40 | double* n4 blind* | 26,809 |
| S39 | rct or randomised control trial | 6,915 |
| S38 | random* | 169,730 |
| S37 | trial* | 186,952 |
| S36 | (MH "Randomized Controlled Trials") | 28,011 |
| S35 | S20 OR S33 OR S34 | 7,988 |
| S34 | synbio* | 84 |
| S33 | S21 OR S22 OR S23 OR S24 OR S25 OR S26 OR S27 OR S28 OR S29 OR S30 OR S31 OR S32 | 1,137 |
| S32 | lactulos* | 219 |
| S31 | trans-galactooligosaccharide* | 1 |
| S30 | transgalactooligosaccharide* | 1 |
| S29 | galacto-oligosaccharide* | 18 |
| S28 | galactooligosaccharide* | 18 |
| S27 | fructo-oligosaccharide* | 63 |
| S26 | fructooligosaccharide* | 54 |
| S25 | oligofructose* | 38 |
| S24 | fructan* | 35 |
| S23 | inulin* | 209 |
| S22 | prebio* | 684 |
| S21 | (MH "Prebiotics") | 467 |
| S20 | S5 OR S6 OR S7 OR S8 OR S9 OR S10 OR S11 OR S12 OR S13 OR S14 OR S15 OR S16 OR S17 OR S18 OR S19 | 7,332 |
| S19 | enterococc* n4 faecium* | 288 |
| S18 | (MH "Enterococcus Faecium") | 133 |
| S17 | salivarius* | 65 |
| S16 | escherichia* n4 coli* | 3,332 |
| S15 | (MH "Escherichia Coli") | 1,546 |
| S14 | freudenreichii* | 9 |
| S13 | boulardii* | 53 |
| S12 | streptococc* N4 thermophil* | 30 |
| S11 | clausii* | 1 |
| S10 | bifidobacteri* | 488 |
| S9 | (MH "Bifidobacterium") | 289 |
| S8 | lactobacill* | 1,298 |
| S7 | (MH "Lactobacillus") | 882 |
| S6 | probio* | 3,094 |
| S5 | (MH "Probiotics") | 2,769 |
| S4 | S1 OR S2 OR S3 | 114,633 |
| S3 | (MH "Pregnancy Complications") | 8,091 |
| S2 | pregnan* | 114,633 |
| S1 | (MH "Pregnancy") | 102,664 |
