# Supplementary material for: Pregnancy outcomes in women taking probiotics or prebiotics: a systematic review and meta-analysis
Source: BMC Pregnancy Childbirth. 2018 Jan 8;18:14. doi: 10.1186/s12884-017-1629-5 (PMC5759212; doi:10.1186/s12884-017-1629-5)
Supplement: Supplementary file 2 — Tables with additional information and details. (DOCX 43 kb) [file 12884_2017_1629_MOESM2_ESM.docx]

**Table S1: Publications with the same study sample**

| **Reference** | **Other publications using the same study sample** |
| --- | --- |
| Kalliomaki 2001 | Rinne M, Kalliomaki M, Arvilommi H, et al. Effect of probiotics and breastfeeding on the bifidobacterium and lactobacillus/enterococcus microbiota and humoral immune responses. The Journal of pediatrics. 2005;147(2):186-91.  Rautava S, Kalliomäki M, Isolauri E. Probiotics during pregnancy and breast-feeding might confer immunomodulatory protection against atopic disease in the infant. Journal of Allergy and Clinical Immunology. 2002;109(1):119-21.  Luoto R, Kalliomäki M, Laitinen K, et al. The impact of perinatal probiotic intervention on the development of overweight and obesity: follow-up study from birth to 10 years. International journal of obesity. 2010;34(10):1531-7. |
| Abrahamsson 2007 | Böttcher MF, Abrahamsson TR, Fredriksson M, et al. Low breast milk TGF‐β2 is induced by Lactobacillus reuteri supplementation and associates with reduced risk of sensitization during infancy. Pediatric allergy and immunology. 2008;19(6):497-504. |
| Kukkonen 2007 | Kukkonen K, Savilahti E, Haahtela T, et al. Long-term safety and impact on infection rates of postnatal probiotic and prebiotic (synbiotic) treatment: randomized, double-blind, placebo-controlled trial. Pediatrics. 2008;122(1):8-12.  Kuitunen M, Kukkonen K, Juntunen-Backman K, et al. Probiotics prevent IgE-associated allergy until age 5 years in cesarean-delivered children but not in the total cohort. Journal of Allergy and Clinical Immunology. 2009;123(2):335-41.  Kukkonen K, Nieminen T, Poussa T, et al. Effect of probiotics on vaccine antibody responses in infancy–a randomized placebo‐controlled double‐blind trial. Pediatric allergy and immunology. 2006;17(6):416-21. |
| Kopp 2008 | Kopp M, Goldstein M, Dietschek A, et al. Lactobacillus GG has in vitro effects on enhanced interleukin‐10 and interferon‐γ release of mononuclear cells but no in vivo effects in supplemented mothers and their neonates. Clinical & Experimental Allergy. 2008;38(4):602-10. |
| Laitinen 2009 | Luoto R, Laitinen K, Nermes M, et al. Impact of maternal probiotic-supplemented dietary counseling during pregnancy on colostrum adiponectin concentration: a prospective, randomized, placebo-controlled study. Early human development. 2012;88(6):339-44.  Hoppu U, Isolauri E, Koskinen P, et al. Maternal dietary counseling reduces total and LDL cholesterol postpartum. Nutrition. 2014;30(2):159-64.  Aaltonen J, Ojala T, Laitinen K, et al. Impact of maternal diet during pregnancy and breastfeeding on infant metabolic programming: a prospective randomized controlled study. European journal of clinical nutrition. 2011;65(1):10-9.  Ilmonen J, Isolauri E, Poussa T, et al. Impact of dietary counselling and probiotic intervention on maternal anthropometric measurements during and after pregnancy: a randomized placebo-controlled trial. Clinical Nutrition. 2011;30(2):156-64.  Luoto R, Laitinen K, Nermes M, et al. Impact of maternal probiotic-supplemented dietary counselling on pregnancy outcome and prenatal and postnatal growth: a double-blind, placebo-controlled study. British journal of nutrition. 2010;103(12):1792-9.  Aaltonen J, Ojala T, Laitinen K, et al. Evidence of infant blood pressure programming by maternal nutrition during pregnancy: a prospective randomized controlled intervention study. The Journal of pediatrics. 2008;152(1):79-84. e2.  Huurre A, Laitinen K, Rautava S, et al. Impact of maternal atopy and probiotic supplementation during pregnancy on infant sensitization: a double‐blind placebo‐controlled study. Clinical & Experimental Allergy. 2008;38(8):1342-8.  Kaplas N, Isolauri E, Lampi A-M, et al. Dietary counseling and probiotic supplementation during pregnancy modify placental phospholipid fatty acids. Lipids. 2007;42(9):865-70. |
| Kim 2010 | Kim JY, Choi YO, Kwon JH, et al. Clinical effects of probiotics are associated with increased transforming growth factor-β responses in infants with high-risk allergy. Journal of the Korean Society for Applied Biological Chemistry. 2011;54(6):944-8. |
| Dotterud 2010 | Simpson MR, Dotterud CK, Storrø O, et al. Perinatal probiotic supplementation in the prevention of allergy related disease: 6 year follow up of a randomised controlled trial. BMC dermatology. 2015;15(1):13.  Dotterud CK, Avershina E, Sekelja M, et al. Does maternal perinatal probiotic supplementation alter the intestinal microbiota of mother and child? Journal of pediatric gastroenterology and nutrition. 2015;61(2):200-7. |
| Asemi 2011 | Asemi Z, Jazayeri S, Najafi M, et al. Effect of daily consumption of probiotic yogurt on oxidative stress in pregnant women: a randomized controlled clinical trial. Annals of Nutrition and Metabolism. 2012;60(1):62-8.  Asemi Z, Samimi M, Tabasi Z, et al. Effect of daily consumption of probiotic yoghurt on lipid profiles in pregnant women: a randomized controlled clinical trial. The Journal of Maternal-Fetal & Neonatal Medicine. 2012;25(9):1552-6.  Asemi Z, Samimi M, Tabassi Z, et al. Effect of daily consumption of probiotic yoghurt on insulin resistance in pregnant women: a randomized controlled trial. European journal of clinical nutrition. 2013;67(1):71-4. |

**Table S2: Other secondary outcomes**

| **Outcome** | **Intervention** | **Studies** | **N** | **I^2^** | **RR/MD (95% CI)** |
| --- | --- | --- | --- | --- | --- |
| Macrosomia (>4000 g) | Probiotics | 3 | 343 | 0% | RR 1.08 (0.72 to 1.63) |
|  | Prebiotics | 0 | - | - | - |
| Birth length (*cm*) | Probiotics | 4 | 528 | 20% | MD 0.22 cm (-0.23 to 0.68) |
|  | Prebiotics | 1 | 116 | - | MD -0.64 cm (-1.72 to 0.44) |
| Ponderal index | Probiotics | 1 | 136 | - | MD 0.50 (-0.66 to 1.66) |
|  | Prebiotics | 0 | - | - | - |
| Head circumference (*cm*) | Probiotics | 3 | 348 | 0% | MD 0.02 cm (-0.33 to 0.36) |
|  | Prebiotics | 1 | 129 | - | MD 0.23 cm (-0.55 to 1.01) |
| PTB <35 weeks | Probiotics | 1 | 82 | - | RR 2.00 (0.19 to 21.21) |
|  | Prebiotics | 0 | - | - | - |
| NICU | Probiotics | 3 | 377 | 0% | RR 1.03 (0.62 to 1.72) |
|  | Prebiotics | 0 | - | - | - |
| Low Apgar score at 5 min. | Probiotics | 1 | 138 | - | RR 3.46 (0.14 to 83.52) |
|  | Prebiotics | 0 | - | - | - |
| Umbilical cord pH ≤7.2 | Probiotics | 0 | - | - | - |
|  | Prebiotics | 1 | 116 | - | RR 0.92 (0.73 to 1.16) |
| Gestational weight gain (*kg*) | Probiotics | 3 | 324 | 68% | MD 0.13 kg (-1.98 to 2.23) |
|  | Prebiotics | 0 | - | - | - |
| Changes in BMI (*kg/m^2^*) | Probiotics | 1 | 60 | - | MD 0.00 kg/m^2^ (-0.29 to 0.29) |
|  | Prebiotics | 0 | - | - | - |
| Pregnancy induced hypertension | Probiotics | 1 | 136 | - | RR 1.99 (0.49 to 7.99) |
|  | Prebiotics | 0 | - | - | - |
| Impaired glucose test | Probiotics | 1 | 136 | - | RR 1.04 (0.40 to 2.72) |
|  | Prebiotics | 0 | - | - | - |
| Caesarian section | Probiotics | 9 | 1208 | 9% | RR 0.83 (0.67 to 1.04) |
|  | Prebiotics | 0 | - | - | - |
| Fasting Plasma Glucose (*mg/dL*) | Probiotics | 6 | 507 | 95% | MD -2.85 mg/dL (-7.97 to 2.27) |
|  | Prebiotics | 0 | - | - | - |
| HOMA-IR | Probiotics | 6 | 545 | 79% | **MD -0.49 (-0.91 to -0.07)** |
|  | Prebiotics | 0 | - | - | - |
| Insulin (*𝜇IU/mL*) | Probiotics | 6 | 496 | 83% | **MD -2.22** 𝜇IU/mL **(-4.26 to -0.18)** |
|  | Prebiotics | 0 | - | - | - |
| HOMA-BC | Probiotics | 1 | 60 | - | **MD -16.90 (-32.51 to -1.29)** |
|  | Prebiotics | 0 | - | - | - |
| QUICKI | Probiotics | 2 | 116 | 86% | MD 0.01 (0.00 to 0.01) |
|  | Prebiotics | 0 | - | - | - |

No study reported information on neonatal death, bacterial vaginosis or urinary tract infections. Bolded results are statistically significant. PTB: preterm birth; NICU: neonatal intensive care unit; BMI: Body Mass Index; HOMA-IR: Homeostatic model assessment insulin resistance; HOMA-BC: Homeostatic model assessment β-cell function; QUICKI: quantitative insulin sensitivity check index

**Table S3: Subgroup analyses by reported conflicts of interest:**

| **Outcome** | **Subgroups** | **Studies** | **N** | **I^2^** | **RR/MD (95% CI)** | **Subgroup differences** |
| --- | --- | --- | --- | --- | --- | --- |
| **PTB <34** | Probiotics |  |  |  |  |  |
|  | No conflicts of interest reported | 1 | 82 | - | 1.00 (0.06 to 15.45) | *p* = 0.76 |
|  | Conflicts of interest unclear/not reported | 2 | 640 | 7% | 0.68 (0.10 to 4.61) |  |
|  | Potential conflicts of interest | 2 | 295 | 0% | 2.05 (0.22 to 19.40) |  |
| **PTB <37** | Probiotics |  |  |  |  |  |
|  | No conflicts of interest reported | 1 | 82 | - | 2.00 (0.19 to 21.21) | *p* = 0.80 |
|  | Conflicts of interest unclear/not reported | 4 | 1078 | 27% | 1.19 (0.53 to 2.63) |  |
|  | Potential conflicts of interest | 6 | 1324 | 0% | 0.98 (0.55 to 1.72) |  |
| Gestational age *(weeks)* | Probiotics * |  |  |  |  |  |
|  | No conflicts of interest reported | 2 | 165 | 35% | 0.17 (-0.22 to 0.56) | *p* = 0.58 |
|  | Conflicts of interest unclear/not reported | 3 | 363 | 0% | -0.05 (-0.34 to 0.23) |  |
|  | Potential conflicts of interest | 3 | 605 | 1% | 0.11 (-0.14 to 0.35) |  |
| Birth weight *(grams)* | Probiotics * |  |  |  |  |  |
|  | No conflicts of interest reported | 3 | 225 | 0% | -104.28 (-214.10 to 5.54) | *p* = 0.08 |
|  | Conflicts of interest unclear/not reported | 3 | 363 | 0% | 36.36 (-59.19 to 131.91) |  |
|  | Potential conflicts of interest | 4 | 1020 | 0% | 35.55 (-25.33 to 96.43) |  |
| SGA | Probiotics |  |  |  |  |  |
|  | No conflicts of interest reported | 1 | 66 | - | 11.00 (0.63 to 191.27) | *p* = 0.13 |
|  | Conflicts of interest unclear/not reported | 1 | 138 | - | 0.54 (0.20 to 1.47) |  |
|  | Potential conflicts of interest | 1 | 114 | - | 1.04 (0.39 to 2.76) |  |
| LGA | Probiotics |  |  |  |  |  |
|  | No conflicts of interest reported | 1 | 66 | - | 0.67 (0.12 to 3.73) | *p* = 0.90 |
|  | Conflicts of interest unclear/not reported | 1 | 136 | - | 1.02 (0.36 to 2.89) |  |
|  | Potential conflicts of interest | 1 | 114 | - | 1.04 (0.32 to 3.38) |  |
| GDM | Probiotics |  |  |  |  |  |
|  | No conflicts of interest reported | 0 | - | - | - | *p* = 0.95 |
|  | Conflicts of interest unclear/not reported | 1 | 136 | - | 1.19 (0.25 to 5.70) |  |
|  | Potential conflicts of interest | 1 | 219 | - | 1.26 (0.56 to 2.83) |  |
| PPROM | Probiotics |  |  |  |  |  |
|  | No conflicts of interest reported | 1 | 66 | - | 1.00 (0.32 to 3.13) | *p* = 0.46 |
|  | Conflicts of interest unclear/not reported | 1 | 300 | - | 1.80 (0.62 to 5.25) |  |
|  | Potential conflicts of interest | 0 | - | - | - |  |

PTB: preterm birth; SGA: Small for gestational age; LGA: large for gestational age; GDM: gestational diabetes mellitus; PPROM: Preterm premature rupture of the membranes

*Two studies (Kalliomaki 2001 and Kim 2010) did not report any information regarding potential conflicts of interest and were not included in either group.

**Table S4: Subgroup analyses by intervention lasting up to the end of pregnancy or not:**

| **Outcome** | **Subgroups** | **Studies** | **N** | **I^2^** | **RR/MD (95% CI)** | **Subgroup differences** |
| --- | --- | --- | --- | --- | --- | --- |
| **PTB <34** | Probiotics |  |  |  |  |  |
|  | Intervention until end of pregnancy | 1 | 231 | - | RR 1.40 (0.06 to 33.87) | *p = 0.84* |
|  | Intervention not until end of pregnancy | 4 | 786 | 0% | RR 0.98 (0.25 to 3.85) |  |
| **PTB <37** | Probiotics |  |  |  |  |  |
|  | Intervention until end of pregnancy | 5 | 1260 | 0% | RR 0.94 (0.53 to 1.68) | *p = 0.51* |
|  | Intervention not until end of pregnancy | 6 | 1224 | 0% | RR 1.24 (0.69 to 2.25) |  |
| Gestational age (*weeks)* | Probiotics |  |  |  |  |  |
|  | Intervention until end of pregnancy | 7 | 997 | 0% | MD 0.11 weeks (-0.06 to 0.28) | *p = 0.13* |
|  | Intervention not until end of pregnancy | 1 | 136 | - | MD -0.30 weeks (-0.81 to 0.21) |  |
| Birth weight *(grams)* | Probiotics |  |  |  |  |  |
|  | Intervention until end of pregnancy | 8 | 1412 | 0% | MD 16.99 grams (-32 to 66) | *p = 0.47* |
|  | Intervention not until end of pregnancy | 2 | 196 | 11% | MD -40.03 grams (-186 to 106) |  |
| SGA | Probiotics |  |  |  |  |  |
|  | Intervention until end of pregnancy | 2 | 180 | 57% | RR 2.27 (0.26 to 20.12) | *p = 0.24* |
|  | Intervention not until end of pregnancy | 1 | 138 | - | RR 0.54 (0.20 to 1.47) |  |
| LGA | Probiotics |  |  |  |  |  |
|  | Intervention until end of pregnancy | 2 | 180 | 0% | RR 0.90 (0.34 to 2.39) | *p = 0.86* |
|  | Intervention not until end of pregnancy | 1 | 136 | - | RR 1.02 (0.36 to 2.89) |  |
| GDM | Probiotics |  |  |  |  |  |
|  | Intervention until end of pregnancy | 1 | 219 | - | RR 1.26 (0.56 to 2.83) | *p = 0.95* |
|  | Intervention not until end of pregnancy | 1 | 136 | - | RR 1.19 (0.25 to 5.70) |  |
| PPROM | Probiotics |  |  |  |  |  |
|  | Intervention until end of pregnancy | 1 | 66 | - | RR 1.00 (0.32 to 3.13) | *p* = 0.46 |
|  | Intervention not until end of pregnancy | 1 | 300 | - | RR 1.80 (0.62 to 5.25) |  |

PTB: preterm birth; SGA: Small for gestational age; LGA: large for gestational age; GDM: gestational diabetes mellitus; PPROM: Preterm premature rupture of the membranes

**Table S5: Sensitivity analyses**

| **Outcome** | **Analysis** | **Studies** | **N** | **I^2^** | **RR/MD (95% CI)** |
| --- | --- | --- | --- | --- | --- |
| **PTB <34** | Probiotics (all studies) | 5 | 1017 | 0% | RR 1.03 (0.29 to 3.64) |
|  | (1) Singletons confirmed | 5 | 1017 | 0% | RR 1.03 (0.29 to 3.64) |
|  | (2) Combining groups | 4 | 1017 | 0% | RR 1.03 (0.28 to 3.72) |
|  | (3) Low risk of bias | 4 | 935 | 0% | RR 1.04 (0.25 to 4.31) |
| **PTB <37** | Probiotics (all studies) | 11 | 2484 | 0% | RR 1.08 (0.71 to 1.63) |
|  | (1) Singletons confirmed | 10 | 2069 | 0% | RR 0.98 (0.59 to 1.63) |
|  | (2) Combining groups | 10 | 2484 | 0% | RR 1.08 (0.70 to 1.66) |
|  | (3) Low risk of bias | 9 | 2246 | 0% | RR 1.08 (0.70 to 1.66) |
| Gestational age *(weeks)* | Probiotics (all studies) | 8 | 1133 | 0% | MD 0.07 weeks (-0.09 to 0.23) |
|  | (1) Singletons confirmed | 7 | 1065 | 2% | MD 0.06 weeks (-0.10 to 0.23) |
|  | (2) Combining groups | 8 | 1133 | 0% | MD 0.07 weeks (-0.09 to 0.23) |
|  | (3) Low risk of bias | 7 | 1065 | 2% | MD 0.06 weeks (-0.10 to 0.23) |
| Birth weight *(grams)* | Probiotics (all studies) | 10 | 1608 | 0% | MD 10.66 grams (-35.85 to 57.18) |
|  | (1) Singletons confirmed | 8 | 1125 | 0% | MD -16.22 grams (-72.97 to 40.54) |
|  | (2) Combining groups | 10 | 1608 | 0% | MD 10.66 grams (-35.85 to 57.18) |
|  | (3) Low risk of bias | 9 | 1540 | 0% | MD 5.74 grams (-42.39 to 53.88) |
| SGA | Probiotics (all studies) | 3 | 318 | 50% | RR 1.03 (0.35 to 3.06) |
|  | (1) Singletons confirmed | 3 | 318 | 50% | RR 1.03 (0.35 to 3.06) |
|  | (2) Combining groups | 3 | 318 | 50% | RR 1.03 (0.35 to 3.06) |
|  | (3) Low risk of bias | 3 | 318 | 50% | RR 1.03 (0.35 to 3.06) |
| LGA | Probiotics (all studies) | 3 | 316 | 0% | RR 0.96 (0.47 to 1.94) |
|  | (1) Singletons confirmed | 3 | 316 | 0% | RR 0.96 (0.47 to 1.94) |
|  | (2) Combining groups | 3 | 316 | 0% | RR 0.96 (0.47 to 1.94) |
|  | (3) Low risk of bias | 3 | 316 | 0% | RR 0.96 (0.47 to 1.94) |
| GDM | Probiotics (all studies) | 2 | 355 | 0% | RR 1.25 (0.61 to 2.56) |
|  | (1) Singletons confirmed | 2 | 355 | 0% | RR 1.25 (0.61 to 2.56) |
|  | (2) Combining groups | 2 | 355 | 0% | RR 1.25 (0.61 to 2.56) |
|  | (3) Low risk of bias | 2 | 355 | 0% | RR 1.25 (0.61 to 2.56) |
| PPROM | Probiotics (all studies) | 2 | 366 | 0% | RR 1.37 (0.63 to 2.99) |
|  | (1) Singletons confirmed | 2 | 366 | 0% | RR 1.37 (0.63 to 2.99) |
|  | (2) Combining groups | 2 | 366 | 0% | RR 1.37 (0.63 to 2.99) |
|  | (3) Low risk of bias | 2 | 366 | 0% | RR 1.37 (0.63 to 2.99) |
| FPG (*mg/dL*) | Probiotics (all studies) | 6 | 507 | 95% | MD -2.85 mg/dL (-7.97 to 2.27) |
|  | (4) Control without probiotics | 5 | 437 | 96% | MD -2.62 mg/dL (-8.07 to 2.84) |
| HOMA-IR | Probiotics (all studies) | 6 | 545 | 79% | **MD -0.49 (-0.91 to -0.07)** |
|  | (4) Control without probiotics | 5 | 475 | 81% | MD -0.42 (-0.90 to 0.05) |
| Insulin (*𝜇IU/mL*) | Probiotics (all studies) | 6 | 496 | 83% | **MD -2.22** 𝜇IU/mL **(-4.26 to -0.18)** |
|  | (4) Control without probiotics | 5 | 426 | 85% | MD -1.97 𝜇IU/mL (-4.26 to 0.33) |

Gray font indicates analyses that are identical to the original meta-analysis including all studies (because no study was excluded). Bolded results are statistically significant. PTB: preterm birth; SGA: Small for gestational age; LGA: large for gestational age; GDM: gestational diabetes mellitus; PPROM: Preterm premature rupture of the membranes; FPG: Fasting Plasma Glucose; HOMA-IR: Homeostatic model assessment insulin resistance

(1) Analyses excluding studies without confirmation of singletons only,

(2) Combining multiple independent comparisons in one study using fixed-effects meta-analysis before pooling with rest of studies

(3) Analyses excluding studies with unclear or high risk of bias

(4) Analyses excluding study with conventional yogurt (with starter cells of probiotics) as control group

**Table S6: Fasting Plasma Glucose: Inputing data using correlation values of different studies**

| **Outcome** | **Studies** | **N** | **I^2^** | **RR/MD (95% CI)** |
| --- | --- | --- | --- | --- |
| FPG (using correlation in Jafernejad 2016) | 6 | 507 | 95% | MD -2.85 mg/dL (-7.97 to 2.27) |
| FPG (using correlation in Asemi 2011) | 6 | 507 | 95% | MD -2.90 mg/dL (-8.18 to 2.39) |
| FPG (using correlation in Doloktah 2011) | 6 | 507 | 95% | MD -2.85 mg/dL (-7.98 to 2.27) |
| FPG (using correlation in Karamali 2016) | 6 | 507 | 95% | MD -2.91 mg/dL (-8.25 to 2.42) |

FPG: Fasting Plasma Glucose

**Table S7: HOMA-IR: Inputing data using correlation values of different studies**

| **Outcome** | **Studies** | **N** | **I^2^** | **RR/MD (95% CI)** |
| --- | --- | --- | --- | --- |
| HOMA-IR (using correlation in Jafernejad 2016) | 6 | 545 | 79% | MD -0.49 (-0.91 to -0.07) |
| HOMA-IR (using correlation in Asemi 2011) | 6 | 545 | 80% | MD -0.48 (-0.90 to -0.06) |
| HOMA-IR (using correlation in Doloktah 2011) | 6 | 545 | 74% | MD -0.52 (-0.95 to -0.09) |
| HOMA-IR (using correlation in Karamali 2016) | 6 | 275 | 79% | MD -0.49 (-0.91 to -0.07) |

HOMA-IR: Homeostatic model assessment insulin resistance

**Table S8: Insulin: Inputing data using correlation values of different studies**

| **Outcome** | **Studies** | **N** | **I^2^** | **RR/MD (95% CI)** |
| --- | --- | --- | --- | --- |
| Insulin (using correlation in Jafernejad 2016) | 6 | 496 | 83% | MD -2.22 𝜇IU/mL (-4.26 to -0.18) |
| Insulin (using correlation in Asemi 2011) | 6 | 496 | 83% | MD -2.22 𝜇IU/mL (-4.26 to -0.18) |
| Insulin (using correlation in Doloktah 2011) | 6 | 496 | 81% | MD -2.30 𝜇IU/mL (-4.38 to -0.21) |
| Insulin (using correlation in Karamali 2016) | 6 | 496 | 84% | MD -2.16 𝜇IU/mL (-4.15 to -0.16) |
