# Supplementary material for: Pregnancy outcomes in women taking probiotics or prebiotics: a systematic review and meta-analysis
Source: BMC Pregnancy Childbirth. 2018 Jan 8;18:14. doi: 10.1186/s12884-017-1629-5 (PMC5759212; doi:10.1186/s12884-017-1629-5)

**Figure S1: Duration and Timing of Probiotics during Pregnancy**

|  | Weeks in which not all women in the sample received the intervention. |
| --- | --- |
|  | Weeks in which all women in the sample received the intervention |

**Figure S2: Preterm birth <34 weeks by species and combinations of species of probiotics**


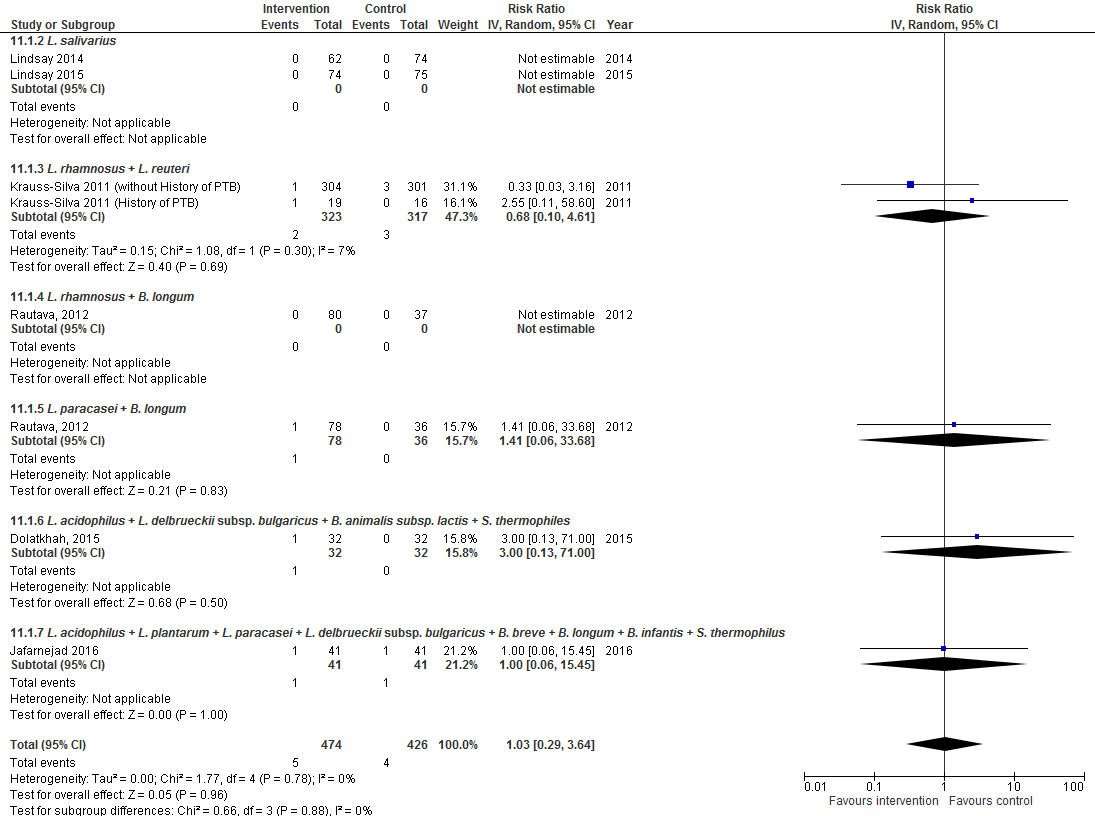


**Figure S3: Preterm birth <37 weeks by species and combinations of species of probiotics**


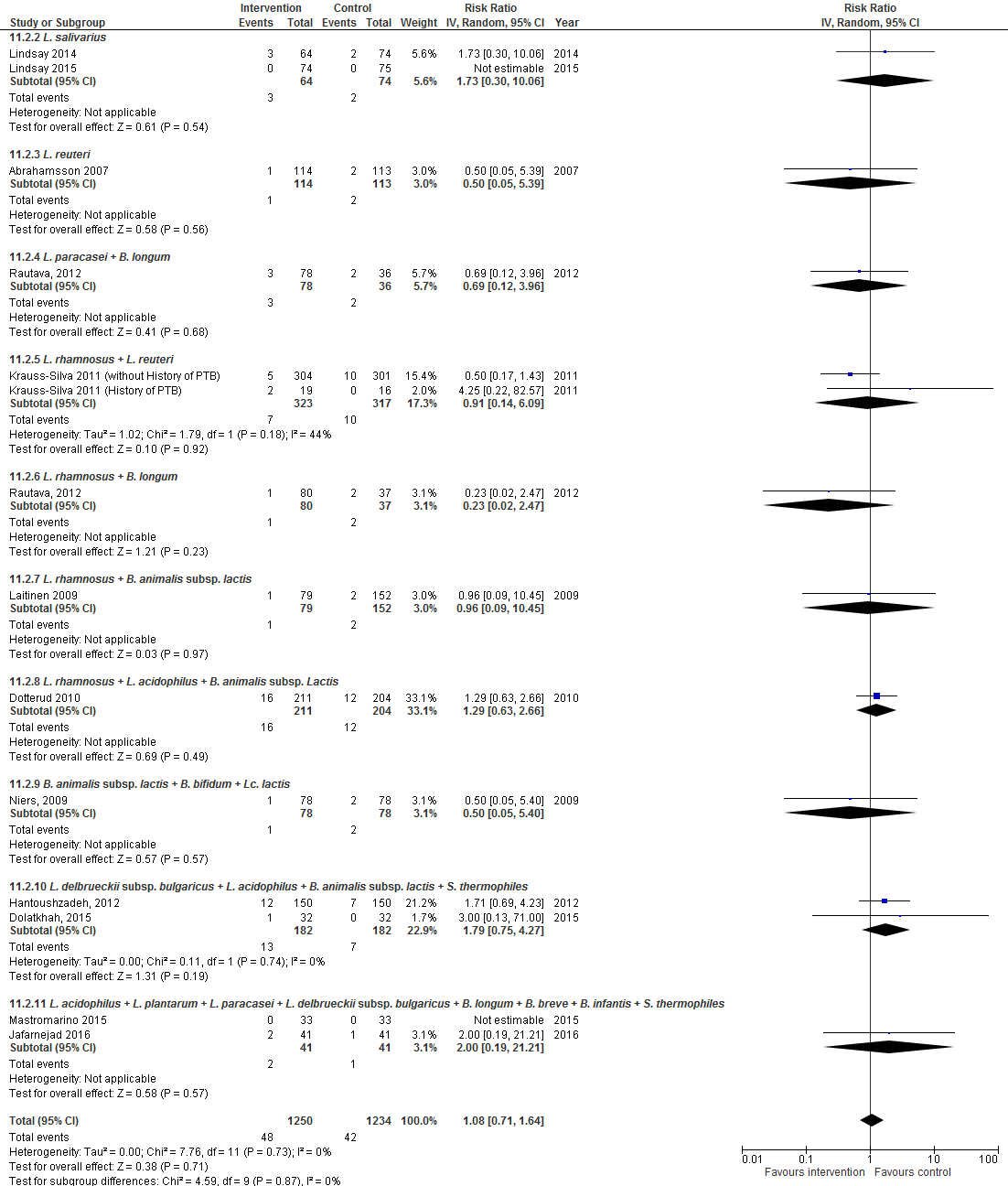

Supplement: Supplementary file 3 — Additional figures of interest. (DOCX 109 kb) [file 12884_2017_1629_MOESM3_ESM.docx]
