# Supplementary material for: Pregnancy outcomes in women taking probiotics or prebiotics: a systematic review and meta-analysis
Source: BMC Pregnancy Childbirth. 2018 Jan 8;18:14. doi: 10.1186/s12884-017-1629-5 (PMC5759212; doi:10.1186/s12884-017-1629-5)
Supplement: Supplementary file 4 — Individual study data.docx; Individual study data; Individual study data for all outcomes, comparisons and analyses. (DOCX 1588 kb) [file 12884_2017_1629_MOESM4_ESM.docx]

# Additional file 4

# Appendix B: Individual study data

[Meta-analyses separating probiotics from prebiotics 5](#_Toc476133231)

[Overall analyses 5](#_Toc476133232)

[Preterm birth <34 weeks 5](#_Toc476133233)

[Preterm birth <37 weeks 6](#_Toc476133234)

[Gestational age (weeks) 7](#_Toc476133235)

[Birth weight (grams) 8](#_Toc476133236)

[Small for gestational age (<10th percentile for gestational age and sex) 9](#_Toc476133237)

[Large for gestational age (>90th percentile for gestational age and sex) 10](#_Toc476133238)

[Gestational diabetes mellitus (GDM) 11](#_Toc476133239)

[Preterm premature rupture of the membranes (PPROM) 12](#_Toc476133240)

[Preterm birth <35 weeks 13](#_Toc476133241)

[Macrosomia (>4000g) 14](#_Toc476133242)

[Birth length (cm) 15](#_Toc476133243)

[Ponderal index (kg/m^3^) 16](#_Toc476133244)

[Head circumference (cm) 17](#_Toc476133245)

[Neonatal Intensive Care Unit (NICU) admission 18](#_Toc476133246)

[Low Apgar score (<7) at 5 minutes 19](#_Toc476133247)

[Umbilical cord pH ≤ 7.2 20](#_Toc476133248)

[Gestational Weight Gain (GWG) (kg) 21](#_Toc476133249)

[Changes in Body Mass Index (kg/m^2^) 22](#_Toc476133250)

[Pregnancy induced hypertension 23](#_Toc476133251)

[Impaired glucose tolerance 24](#_Toc476133252)

[Caesarean section 25](#_Toc476133253)

[HOMA β-cell function 26](#_Toc476133254)

[Quantitative insulin sensitivity check index (QUICKI) 27](#_Toc476133255)

[Fasting plasma glucose (FPG) (mg/dL) (using correlation in Jafernejad 2016) 28](#_Toc476133256)

[HOMA insulin resistance (using correlation in Jafernejad 2016) 29](#_Toc476133257)

[Insulin (𝜇IU/mL) (using correlation in Jafernejad 2016) 30](#_Toc476133258)

[Meta-analyses by probiotic type (genus) 31](#_Toc476133259)

[*Lactobacillus* 31](#_Toc476133260)

[Preterm birth <34 weeks 31](#_Toc476133261)

[Preterm birth <37 weeks 32](#_Toc476133262)

[Gestational age (weeks) 33](#_Toc476133263)

[Birth weight (grams) 34](#_Toc476133264)

[Small for gestational age (<10th percentile for gestational age and sex) 35](#_Toc476133265)

[Large for gestational age (>90th percentile for gestational age and sex) 36](#_Toc476133266)

[Gestational diabetes mellitus (GDM) 37](#_Toc476133267)

[Preterm premature rupture of the membranes (PPROM) 38](#_Toc476133268)

[*Bifidobacterium* 39](#_Toc476133269)

[Preterm birth <34 weeks 39](#_Toc476133270)

[Preterm birth <37 weeks 40](#_Toc476133271)

[Gestational age (weeks) 41](#_Toc476133272)

[Birth weight (grams) 42](#_Toc476133273)

[Small for gestational age (<10th percentile for gestational age and sex) 43](#_Toc476133274)

[Large for gestational age (>90th percentile for gestational age and sex) 44](#_Toc476133275)

[Gestational diabetes mellitus (GDM) 45](#_Toc476133276)

[Preterm premature rupture of the membranes (PPROM) 46](#_Toc476133277)

[*Streptococcus* 47](#_Toc476133278)

[Preterm birth <34 weeks 47](#_Toc476133279)

[Preterm birth <37 weeks 48](#_Toc476133280)

[Gestational age (weeks) 49](#_Toc476133281)

[Birth weight (grams) 50](#_Toc476133282)

[Small for gestational age (<10th percentile for gestational age and sex) 51](#_Toc476133283)

[Large for gestational age (>90th percentile for gestational age and sex) 52](#_Toc476133284)

[Gestational diabetes mellitus (GDM) 53](#_Toc476133285)

[Preterm premature rupture of the membranes (PPROM) 54](#_Toc476133286)

[Meta-analyses by combination of probiotic species 55](#_Toc476133287)

[Preterm birth <34 weeks 55](#_Toc476133288)

[Preterm birth <37 weeks 56](#_Toc476133289)

[Subgroup analyses by reported conflicts of interest 58](#_Toc476133290)

[Preterm birth <34 weeks 58](#_Toc476133291)

[Preterm birth <37 weeks 59](#_Toc476133292)

[Gestational age (weeks) 60](#_Toc476133293)

[Birth weight (grams) 61](#_Toc476133294)

[Small for gestational age (<10th percentile for gestational age and sex) 62](#_Toc476133295)

[Large for gestational age (>90th percentile for gestational age and sex) 63](#_Toc476133296)

[Gestational diabetes mellitus (GDM) 64](#_Toc476133297)

[Preterm premature rupture of the membranes (PPROM) 65](#_Toc476133298)

[Subgroup analyses by intervention lasting until the end of pregnancy or not 66](#_Toc476133299)

[Preterm birth <34 weeks 66](#_Toc476133300)

[Preterm birth <37 weeks 67](#_Toc476133301)

[Gestational age (weeks) 68](#_Toc476133302)

[Birth weight (grams) 69](#_Toc476133303)

[Small for gestational age (<10th percentile for gestational age and sex) 70](#_Toc476133304)

[Large for gestational age (>90th percentile for gestational age and sex) 71](#_Toc476133305)

[Gestational diabetes mellitus (GDM) 72](#_Toc476133306)

[Preterm premature rupture of the membranes (PPROM) 73](#_Toc476133307)

[Sensitivity analyses 74](#_Toc476133308)

[Excluding studies without confirmation of singletons only 74](#_Toc476133309)

[Preterm birth <34 weeks 74](#_Toc476133310)

[Preterm birth <37 weeks 75](#_Toc476133311)

[Gestational age (weeks) 76](#_Toc476133312)

[Birth weight (grams) 77](#_Toc476133313)

[Small for gestational age (<10th percentile for gestational age and sex) 78](#_Toc476133314)

[Large for gestational age (>90th percentile for gestational age and sex) 79](#_Toc476133315)

[Gestational diabetes mellitus (GDM) 80](#_Toc476133316)

[Preterm premature rupture of the membranes (PPROM) 81](#_Toc476133317)

[Combining multiple independent comparisons in one study using fixed-effects meta-analysis before pooling with rest of studies 82](#_Toc476133318)

[Preterm birth <34 weeks 82](#_Toc476133319)

[Preterm birth <37 weeks 83](#_Toc476133320)

[Excluding studies with unclear or high risk of bias 84](#_Toc476133321)

[Preterm birth <34 weeks 84](#_Toc476133322)

[Preterm birth <37 weeks 85](#_Toc476133323)

[Gestational age (weeks) 86](#_Toc476133324)

[Birth weight (grams) 87](#_Toc476133325)

[Small for gestational age (<10th percentile for gestational age and sex) 88](#_Toc476133326)

[Large for gestational age (>90th percentile for gestational age and sex) 89](#_Toc476133327)

[Gestational diabetes mellitus (GDM) 90](#_Toc476133328)

[Preterm premature rupture of the membranes (PPROM) 91](#_Toc476133329)

[Excluding study with conventional yogurt (with starter cells of probiotics) as control group 92](#_Toc476133330)

[Fasting plasma glucose (FPG) (mg/dL) (using correlation in Jafernejad 2016) 92](#_Toc476133331)

[HOMA insulin resistance (using correlation in Jafernejad 2016) 93](#_Toc476133332)

[Insulin (𝜇IU/mL) (using correlation in Jafernejad 2016) 94](#_Toc476133333)

[Inputing data using correlation values of different studies 95](#_Toc476133334)

[Fasting plasma glucose (FPG) (mg/dL) (using correlation in Asemi 2011) 95](#_Toc476133335)

[Fasting plasma glucose (FPG) (mg/dL) (using correlation in Doloktah 2011) 96](#_Toc476133336)

[Fasting plasma glucose (FPG) (mg/dL) (using correlation in Karamali 2016) 97](#_Toc476133337)

[HOMA insulin resistance (using correlation in Asemi 2011) 98](#_Toc476133338)

[HOMA insulin resistance (using correlation in Doloktah 2011) 99](#_Toc476133339)

[HOMA insulin resistance (using correlation in Karamali 2016) 100](#_Toc476133340)

[Insulin (𝜇IU/mL) (using correlation in Asemi 2011) 101](#_Toc476133341)

[Insulin (𝜇IU/mL) (using correlation in Doloktah 2011) 102](#_Toc476133342)

[Insulin (𝜇IU/mL) (using correlation in Karamali 2016) 103](#_Toc476133343)

## Meta-analyses separating probiotics from prebiotics

### Overall analyses

#### Preterm birth <34 weeks


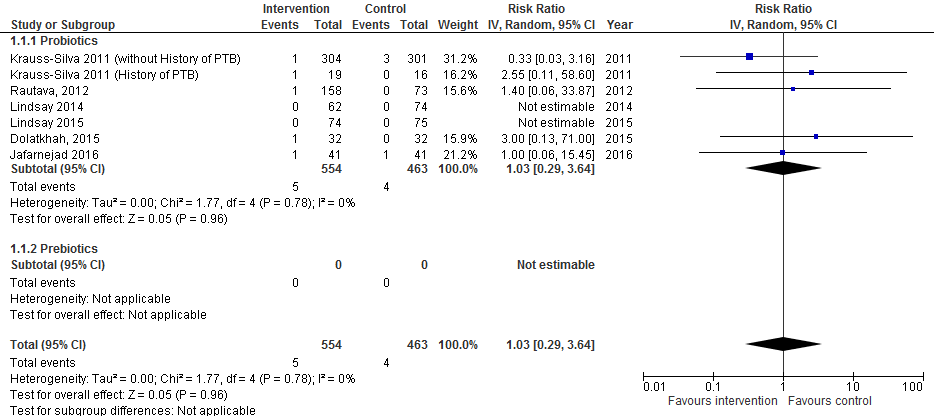


Doloktah 2010: Data provided by authors.

Jafarnejad 2016: Data provided by authors.

Rautava 2012: Data provided by authors. The two intervention groups were combined into one.

#### Preterm birth <37 weeks


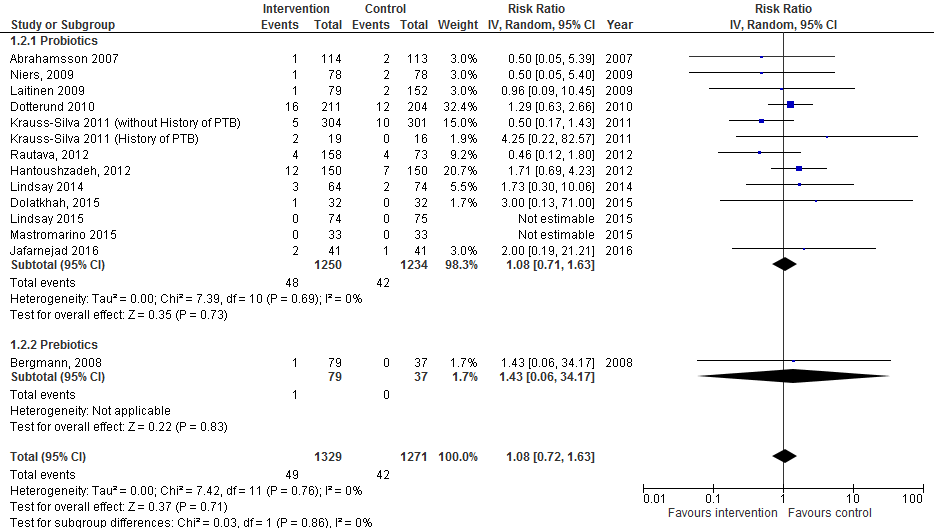


Abrahamsson 2007: Data provided by authors.

Bergmann 2008: The two intervention groups were combined into one.

Dolaktah 2015: Cases excluded from their study due to preterm birth.

Dotterund 2010: Absence of twins not confirmed.

Hantouszhadeh 2012: Absence of twins confirmed by authors.

Jafarnejad 2016: Data provided by authors.

Laitinen 2009: Data provided by authors. The two control groups were combined into one.

Niers 2009: Cases excluded from the study due to preterm birth.

Rautava 2012: Data provided by authors. The two intervention groups were combined into one.

#### Gestational age (weeks)


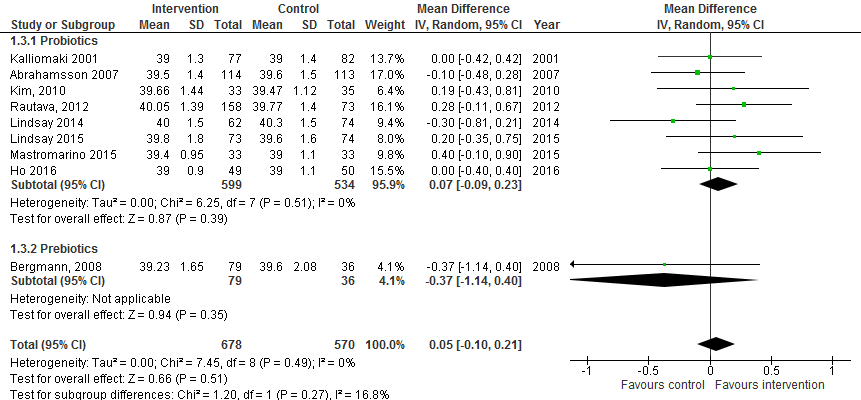


Abrahamsson 2007: Data provided by authors.

Bergmann 2008: The two intervention groups were combined into one.

Boyle 2005: Gestational age reported as median weeks (range) and could not be pooled with the other studies: 39.6 weeks (35.4-42) in the probiotics group vs 39.5 weeks (36-42.3) in the control group.

Kim 2010: Subjects were excluded if they had premature babies delivered at less than 36 weeks of gestation, but none of the participants was excluded for this reason. Unclear if singletons only.

Mastromarino 2015: Exclusion criteria included preterm delivery, but only one woman was excluded because she had no milk.

Ou 2012: Gestational age reported as median weeks (range) and could not be pooled with the other studies: 39 weeks (31-41) in the probiotics group vs 39 weeks (35-41) in the control group.

Rautava 2012: Data provided by authors. The two intervention groups were combined into one.

#### Birth weight (grams)


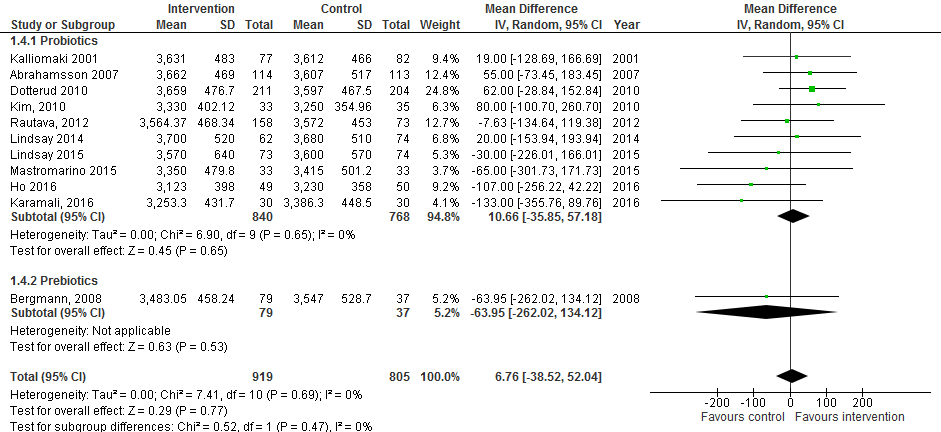


Abrahamsson 2007: Data provided by authors.

Allen 2010: Birth weight reported as median weeks (range) and could not be pooled with the other studies: 3.49 kg (2.1-4.9) in the probiotics group vs 3.55 kg (2-5.2) in the control group.

Bergmann 2008: The two intervention groups were combined into one.

Boyle 2005: Birth weight reported as median weeks (range) and could not be pooled with the other studies: 3560 grams (2324-4970) in the probiotics group vs 3615 grams (2105-5020) in the control group.

Dotterud 2010: Combining Complete cases and drop-outs. Unclear if singletons only.

Karamali 2016: Data (SD) provided by authors.

Kim 2010: Subjects were excluded if they had premature babies delivered at less than 36 weeks of gestation, but none of the participants was excluded for this reason. Unclear if singletons only.

Mastromarino 2015: Exclusion criteria included preterm delivery, but only one woman was excluded because she had no milk.

Rautava 2012: Data provided by authors. The two intervention groups were combined into one.

#### Small for gestational age (<10th percentile for gestational age and sex)


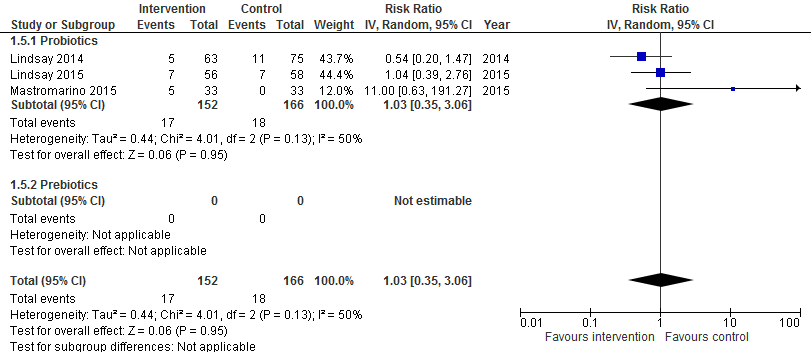


Lindsay 2014: Data provided by authors.

Mastromarino 2015: Data provided by authors.

#### Large for gestational age (>90th percentile for gestational age and sex)


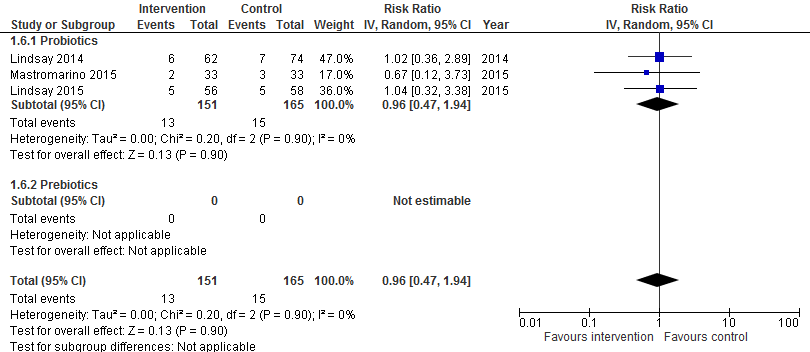


Mastromarino 2015: Data provided by authors.

#### Gestational diabetes mellitus (GDM)


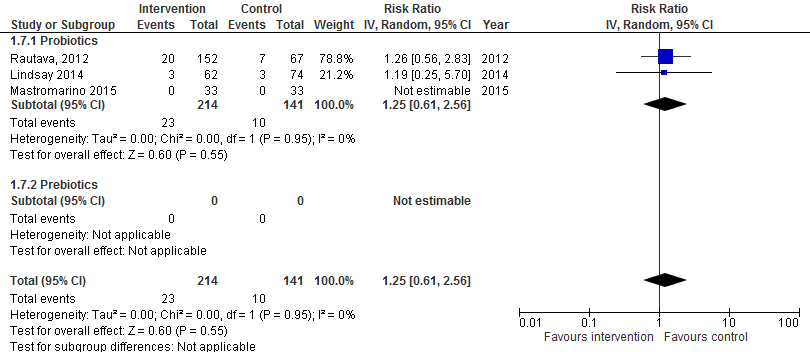


Rautava 2012: Data provided by authors. The two intervention groups were combined into one.

Mastromarino 2015: Data provided by authors.

#### Preterm premature rupture of the membranes (PPROM)


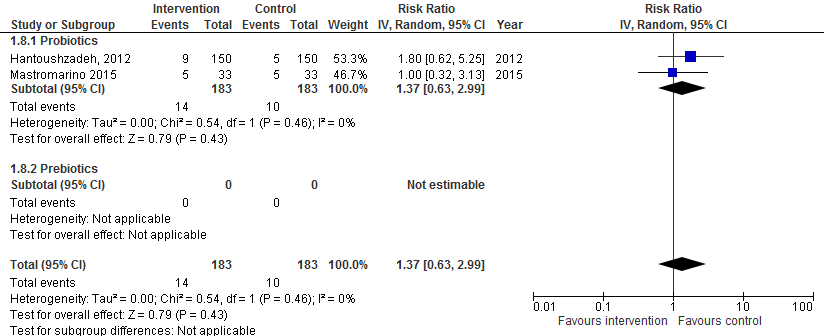


Mastromarino 2015: Data provided by authors.

#### Preterm birth <35 weeks


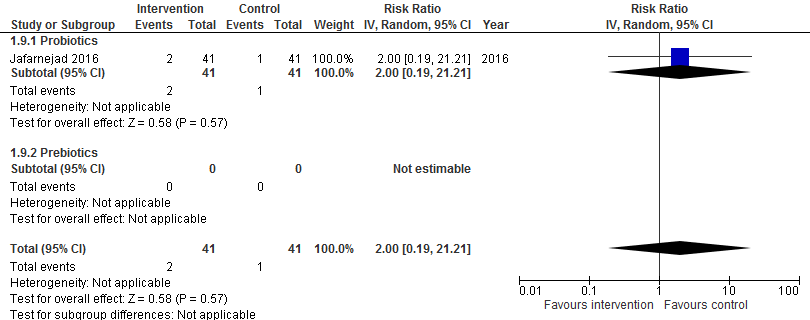


#### Macrosomia (>4000g)


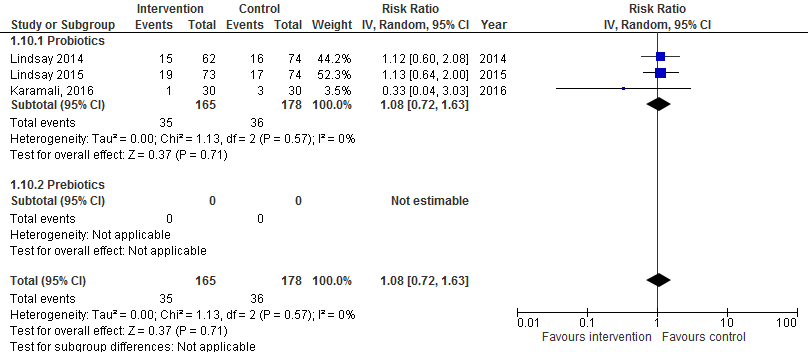


Karamali 2016: Reported as macrosomia, but cut-off value not specified.

#### Birth length (cm)


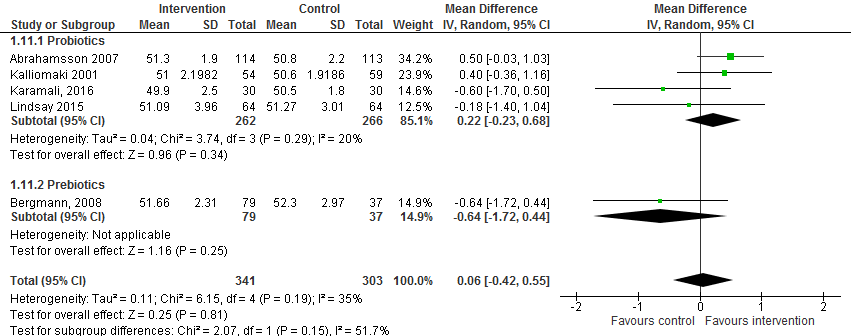


Abrahamsson 2007: Data provided by authors.

Bergmann 2008: The two intervention groups were combined into one.

Karamali 2016: Data provided by authors.

#### Ponderal index (kg/m^3^)


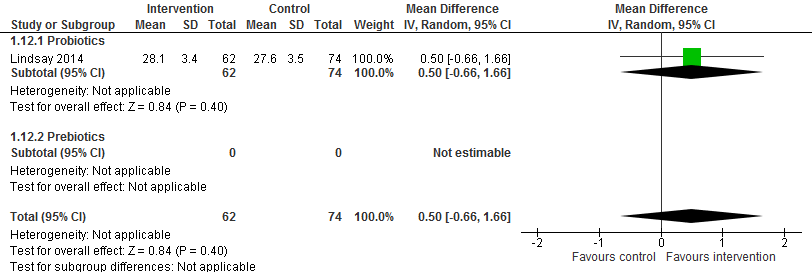


#### Head circumference (cm)


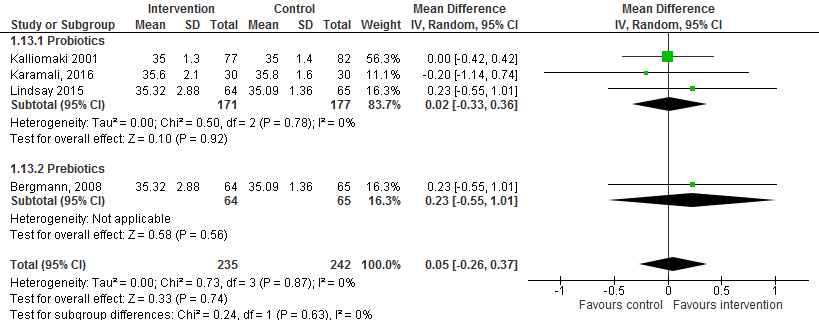


Bergmann 2008: The two intervention groups were combined into one.

Karamali 2016: Data (SD) provided by authors.

#### Neonatal Intensive Care Unit (NICU) admission


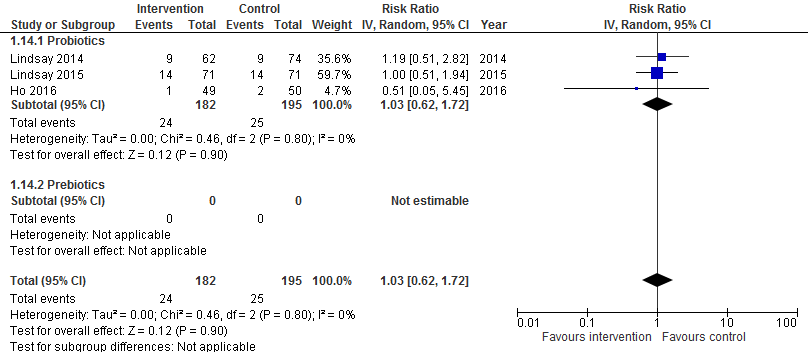


#### Low Apgar score (<7) at 5 minutes


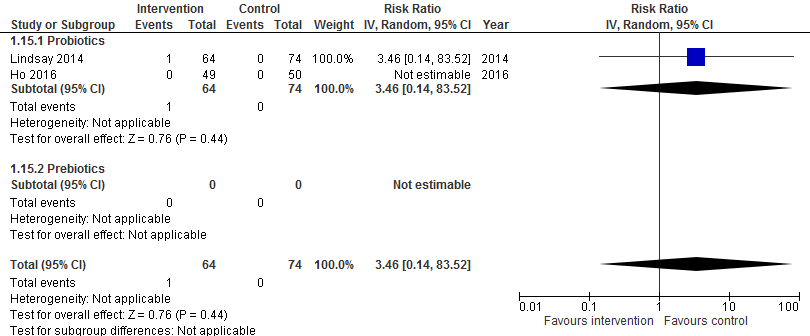


#### Umbilical cord pH ≤ 7.2


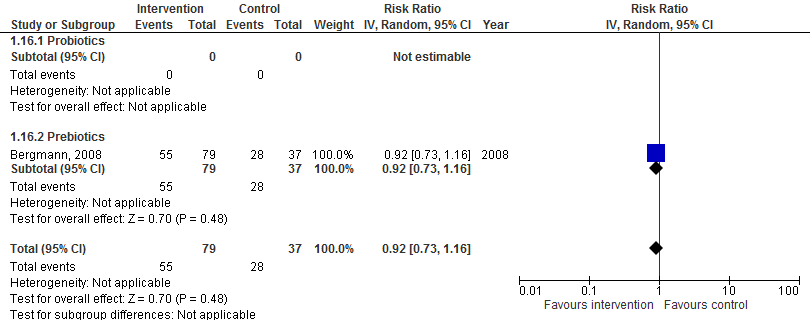


Bergmann 2008: The two intervention groups were combined into one.

#### Gestational Weight Gain (GWG) (kg)


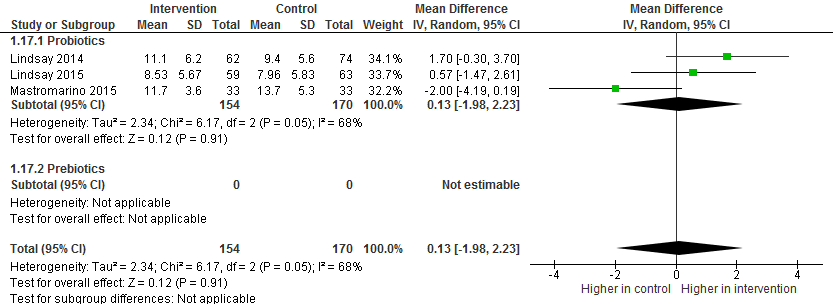


#### Changes in Body Mass Index (kg/m^2^)


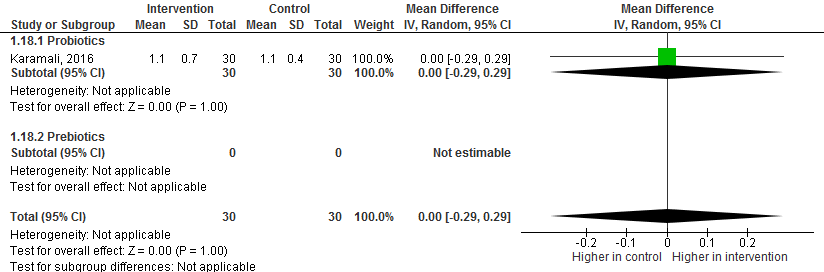


#### Pregnancy induced hypertension


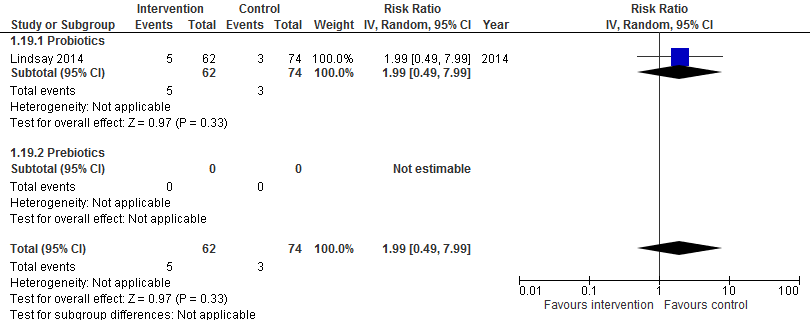


#### Impaired glucose tolerance


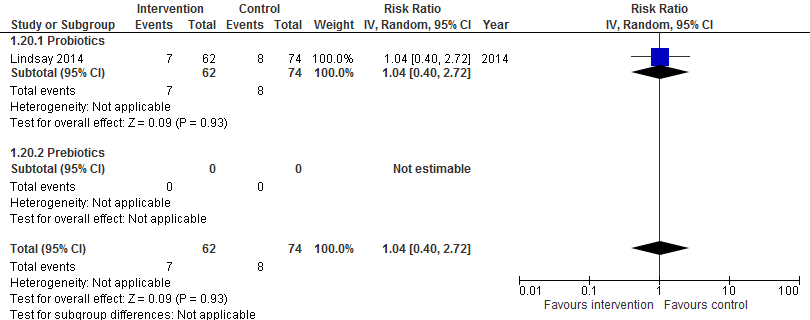


#### Caesarean section


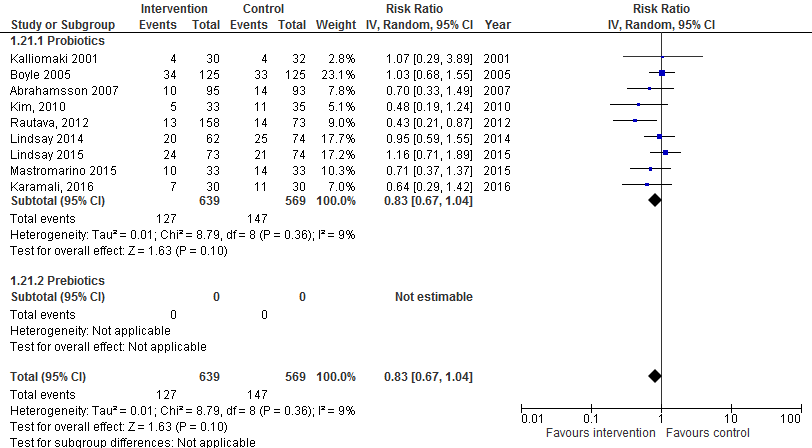


Kim 2010: Subjects were excluded if they had premature babies delivered at less than 36 weeks of gestation, but none of the participants was excluded for this reason. Unclear if singletons only.

Rautava 2012: The two intervention groups were combined into one.

Mastromarino 2015: Exclusion criteria included preterm delivery, but only one woman was excluded because she had no milk.

#### HOMA β-cell function


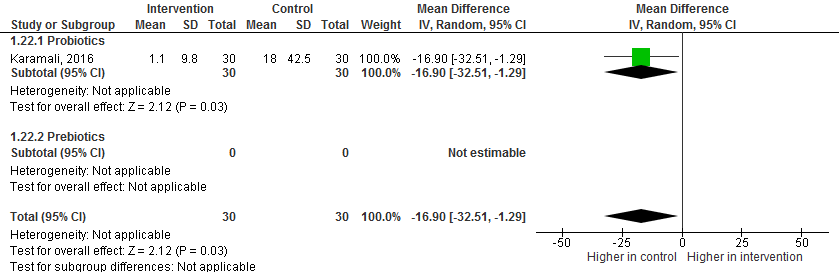


#### Quantitative insulin sensitivity check index (QUICKI)


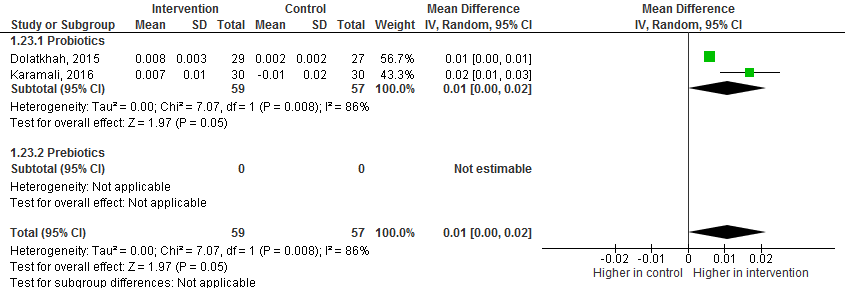


#### Fasting plasma glucose (FPG) (mg/dL) (using correlation in Jafernejad 2016)


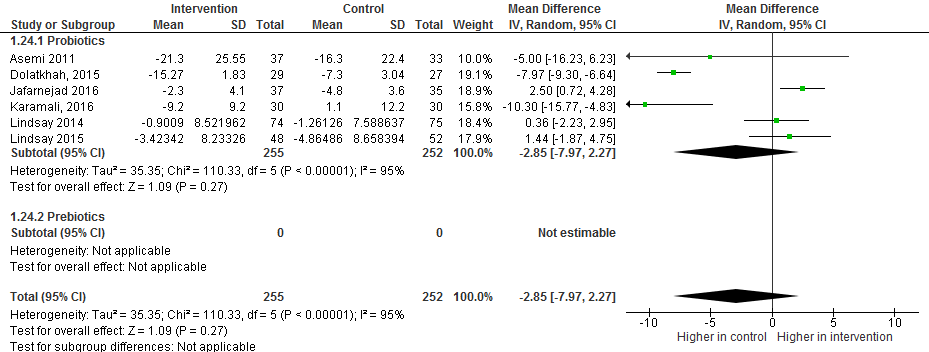


Lindsay 2014: SD of the measures of change were inputed.

Lindsay 2015: SD of the measures of change were inputed. Per protocol cohort.

#### HOMA insulin resistance (using correlation in Jafernejad 2016)


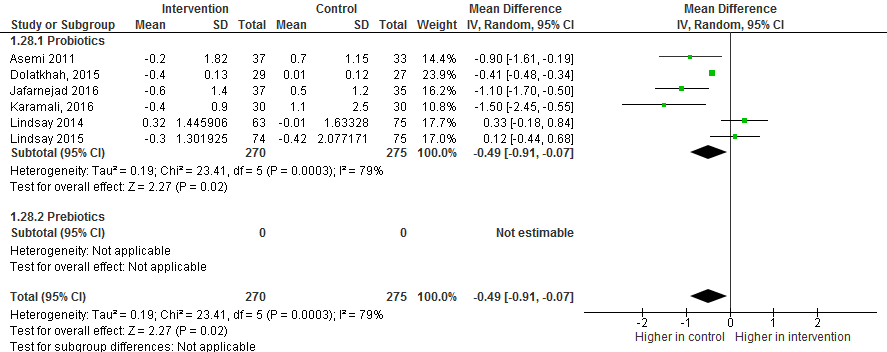


Lindsay 2014: SD of the measures of change were inputed.

Lindsay 2015: SD of the measures of change were inputed. Per protocol cohort.

#### Insulin (𝜇IU/mL) (using correlation in Jafernejad 2016)


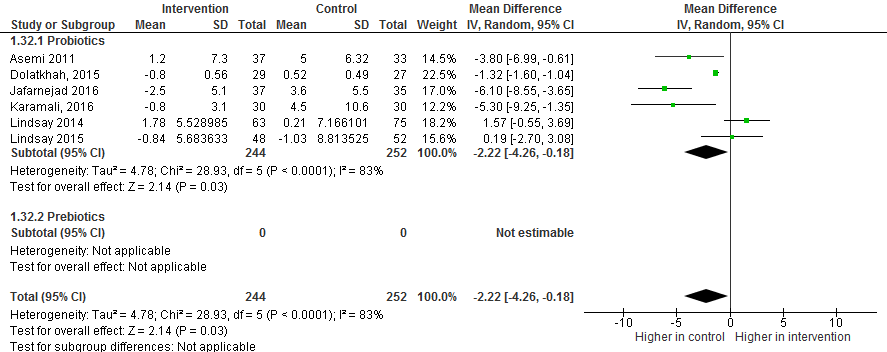


Lindsay 2014: SD of the measures of change were inputed.

Lindsay 2015: SD of the measures of change were inputed. Per protocol cohort.

## Meta-analyses by probiotic type (genus)

### *Lactobacillus*

#### Preterm birth <34 weeks


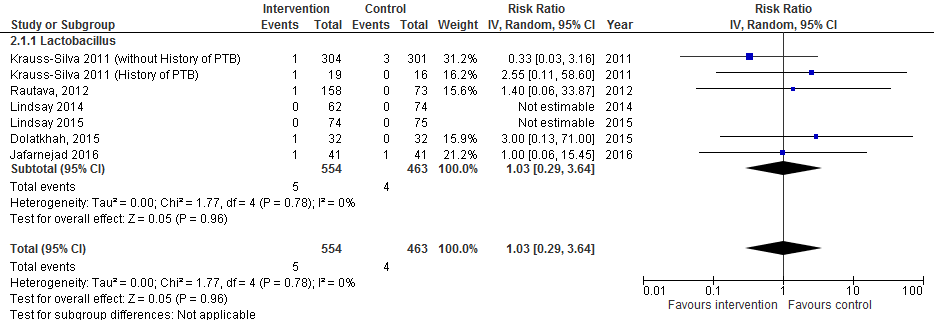


Doloktah 2010: Data provided by authors.

Jafarnejad 2016: Data provided by authors.

Rautava 2012: Data provided by authors. The two intervention groups were combined into one.

#### Preterm birth <37 weeks


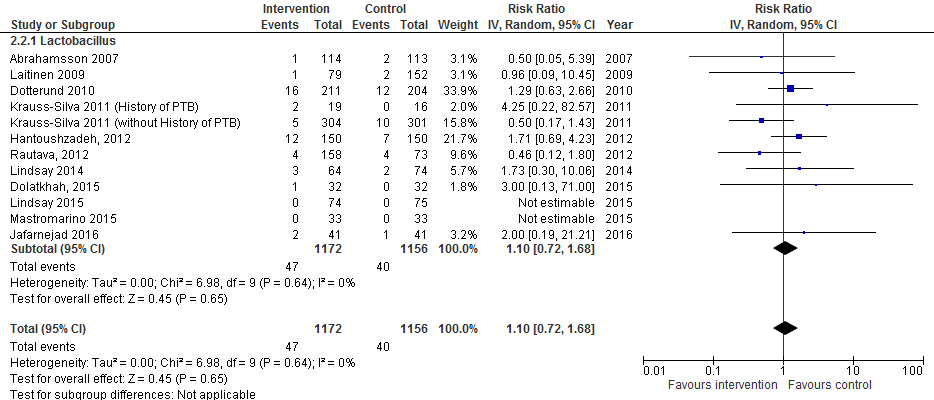


Abrahamsson 2007: Data provided by authors.

Dolaktah 2015: Cases excluded from their study due to preterm birth.

Dotterund 2010: Absence of twins not confirmed.

Hantouszhadeh 2012: Absence of twins confirmed by authors.

Jafarnejad 2016: Data provided by authors.

Laitinen 2009: Data provided by authors. The two control groups were combined into one.

Rautava 2012: Data provided by authors. The two intervention groups were combined into one.

#### Gestational age (weeks)


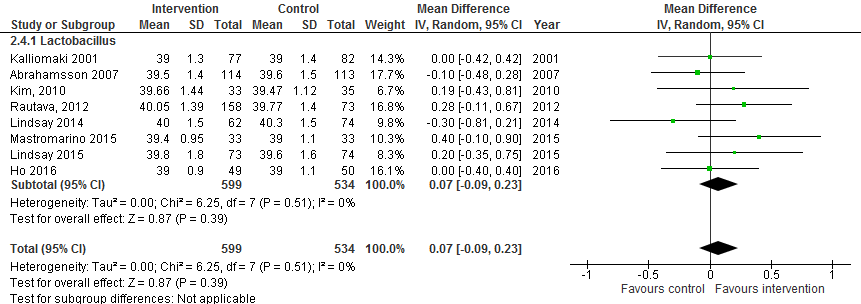


Abrahamsson 2007: Data provided by authors.

Kim 2010: Subjects were excluded if they had premature babies delivered at less than 36 weeks of gestation, but none of the participants was excluded for this reason. Unclear if singletons only.

Mastromarino 2015: Exclusion criteria included preterm delivery, but only one woman was excluded because she had no milk.

Rautava 2012: Data provided by authors. The two intervention groups were combined into one.

#### Birth weight (grams)


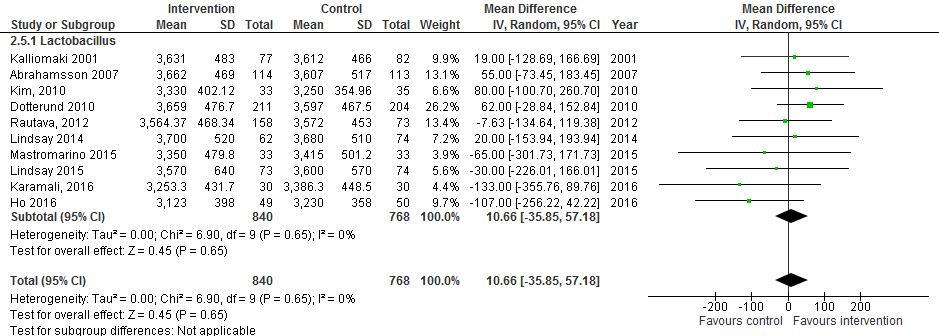


Abrahamsson 2007: Data provided by authors.

Dotterud 2010: Combining Complete cases and drop-outs. Unclear if singletons only.

Karamali 2016: Data (SD) provided by authors.

Kim 2010: Subjects were excluded if they had premature babies delivered at less than 36 weeks of gestation, but none of the participants was excluded for this reason. Unclear if singletons only.

Mastromarino 2015: Exclusion criteria included preterm delivery, but only one woman was excluded because she had no milk.

Rautava 2012: Data provided by authors. The two intervention groups were combined into one.

#### Small for gestational age (<10th percentile for gestational age and sex)


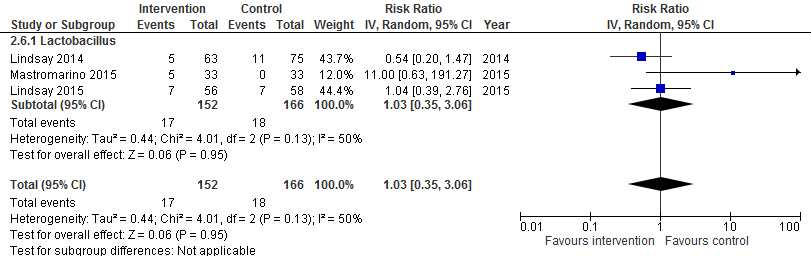


Lindsay 2014: Data provided by authors.

Mastromarino 2015: Data provided by authors.

#### Large for gestational age (>90th percentile for gestational age and sex)


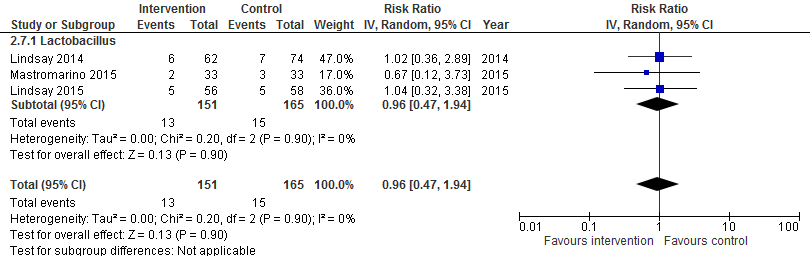


Mastromarino 2015: Data provided by authors.

#### Gestational diabetes mellitus (GDM)


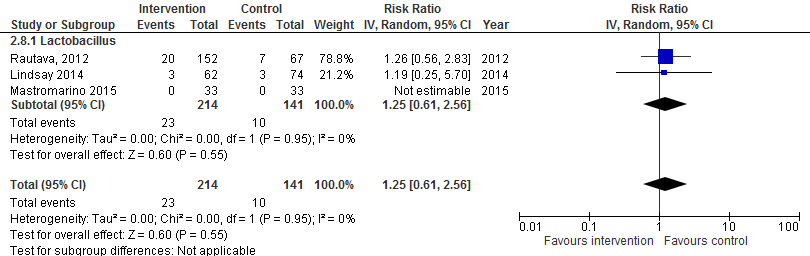


Rautava 2012: Data provided by authors. The two intervention groups were combined into one.

Mastromarino 2015: Data provided by authors.

#### Preterm premature rupture of the membranes (PPROM)


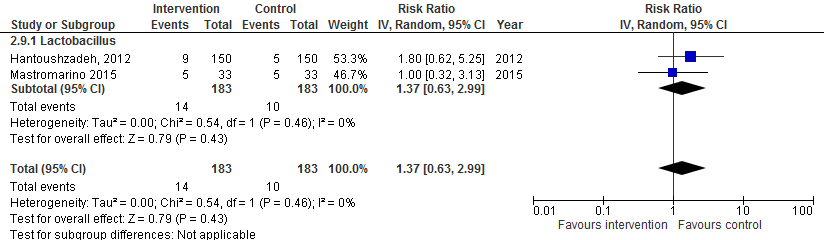


Mastromarino 2015: Data provided by authors.

### *Bifidobacterium*

#### Preterm birth <34 weeks


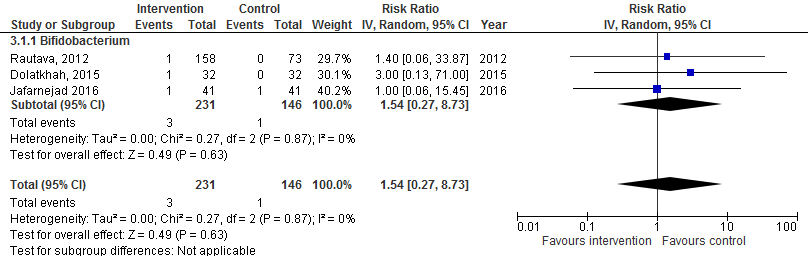


Doloktah 2010: Data provided by authors.

Jafarnejad 2016: Data provided by authors.

Rautava 2012: Data provided by authors. The two intervention groups were combined into one.

#### Preterm birth <37 weeks


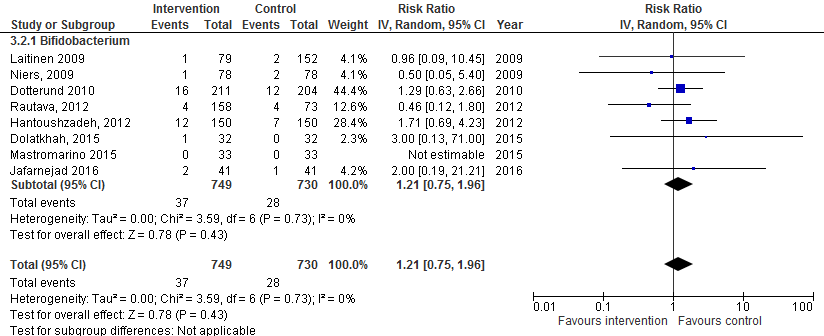


Dolaktah 2015: Cases excluded from their study due to preterm birth.

Dotterund 2010: Absence of twins not confirmed.

Hantouszhadeh 2012: Absence of twins confirmed by authors.

Jafarnejad 2016: Data provided by authors.

Laitinen 2009: Data provided by authors. The two control groups were combined into one.

Niers 2009: Cases excluded from the study due to preterm birth.

Rautava 2012: Data provided by authors. The two intervention groups were combined into one.

#### Gestational age (weeks)


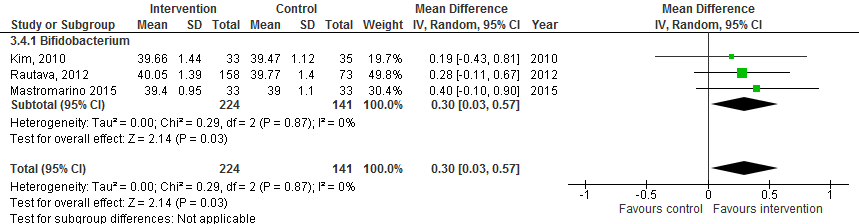


Kim 2010: Subjects were excluded if they had premature babies delivered at less than 36 weeks of gestation, but none of the participants was excluded for this reason. Unclear if singletons only.

Mastromarino 2015: Exclusion criteria included preterm delivery, but only one woman was excluded because she had no milk.

Rautava 2012: Data provided by authors. The two intervention groups were combined into one.

#### Birth weight (grams)


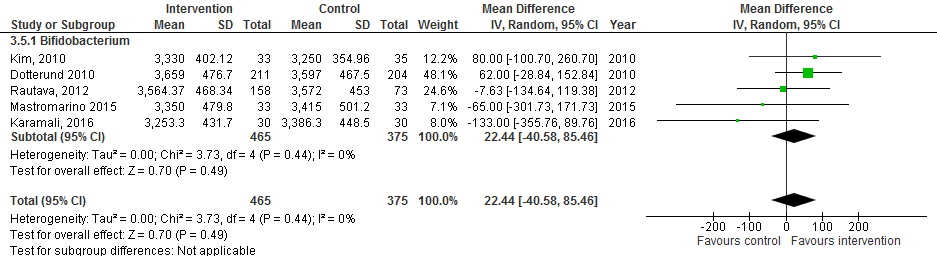


Dotterud 2010: Combining Complete cases and drop-outs. Unclear if singletons only.

Karamali 2016: Data (SD) provided by authors.

Kim 2010: Subjects were excluded if they had premature babies delivered at less than 36 weeks of gestation, but none of the participants was excluded for this reason. Unclear if singletons only.

Mastromarino 2015: Exclusion criteria included preterm delivery, but only one woman was excluded because she had no milk.

Rautava 2012: Data provided by authors. The two intervention groups were combined into one.

#### Small for gestational age (<10th percentile for gestational age and sex)


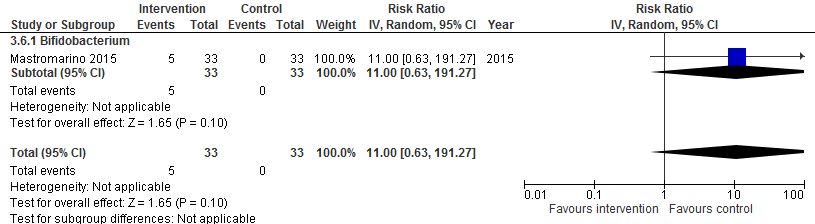


Mastromarino 2015: Data provided by authors.

#### Large for gestational age (>90th percentile for gestational age and sex)


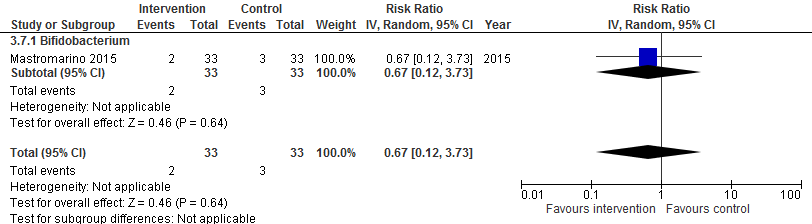


Mastromarino 2015: Data provided by authors.

#### Gestational diabetes mellitus (GDM)


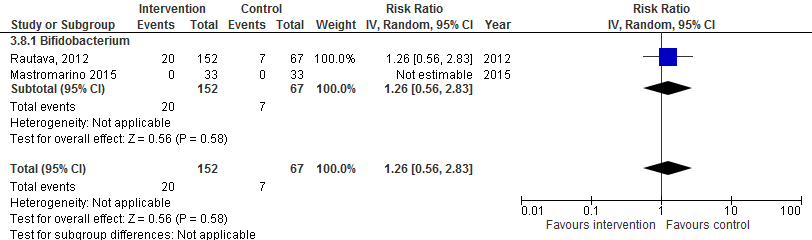


Rautava 2012: Data provided by authors. The two intervention groups were combined into one.

Mastromarino 2015: Data provided by authors.

#### Preterm premature rupture of the membranes (PPROM)


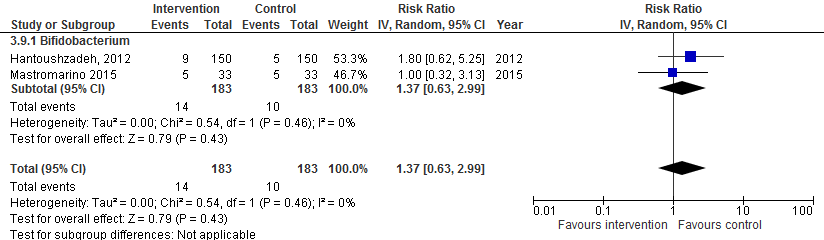


Mastromarino 2015: Data provided by authors.

### *Streptococcus*

#### Preterm birth <34 weeks


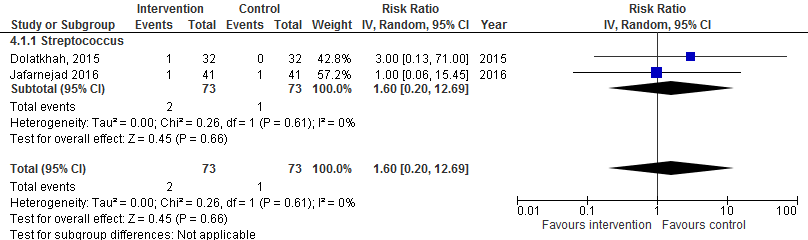


Doloktah 2010: Data provided by authors.

Jafarnejad 2016: Data provided by authors.

#### Preterm birth <37 weeks


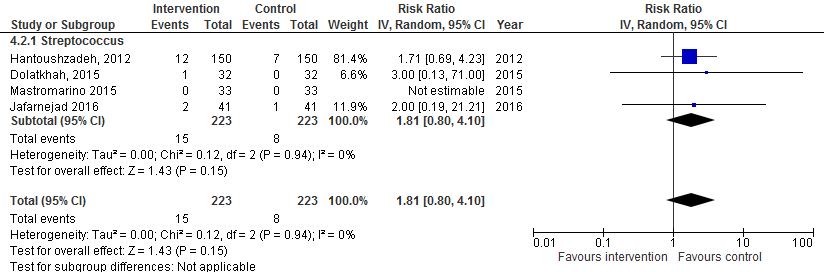


Dolaktah 2015: Cases excluded from their study due to preterm birth.

Hantouszhadeh 2012: Absence of twins confirmed by authors.

Jafarnejad 2016: Data provided by authors.

#### Gestational age (weeks)


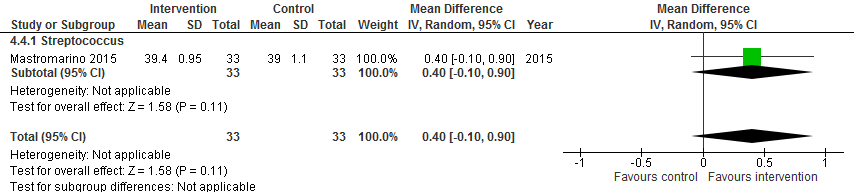


Mastromarino 2015: Exclusion criteria included preterm delivery, but only one woman was excluded because she had no milk.

#### Birth weight (grams)


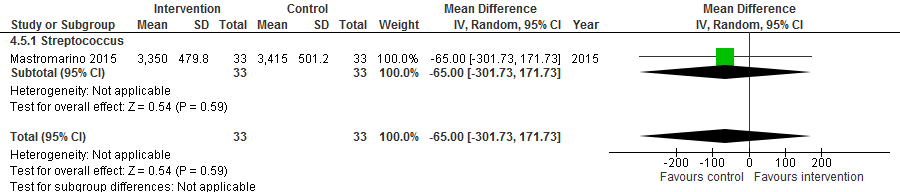


Mastromarino 2015: Exclusion criteria included preterm delivery, but only one woman was excluded because she had no milk.

#### Small for gestational age (<10th percentile for gestational age and sex)


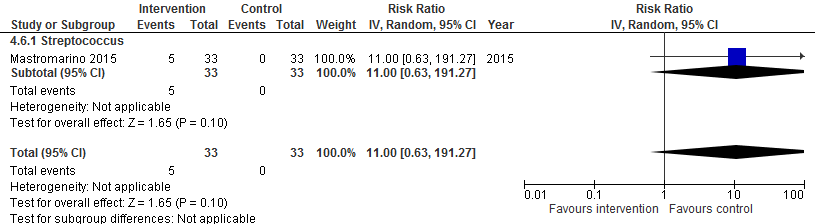


Mastromarino 2015: Data provided by authors.

#### Large for gestational age (>90th percentile for gestational age and sex)


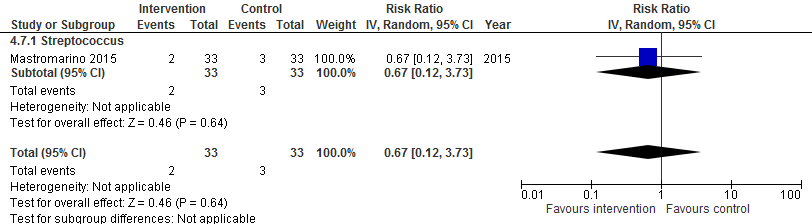


Mastromarino 2015: Data provided by authors.

#### Gestational diabetes mellitus (GDM)


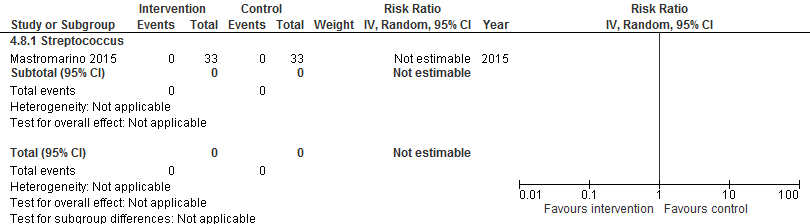


Mastromarino 2015: Data provided by authors.

#### Preterm premature rupture of the membranes (PPROM)


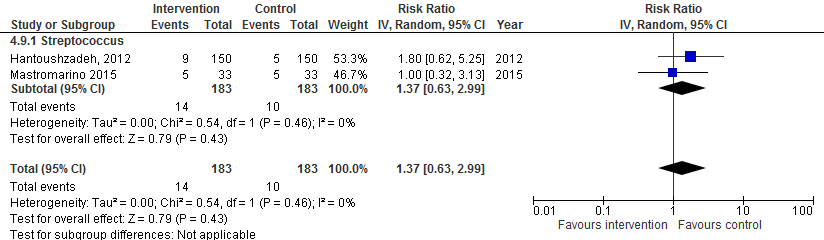


Mastromarino 2015: Data provided by authors.

## Meta-analyses by combination of probiotic species

#### Preterm birth <34 weeks


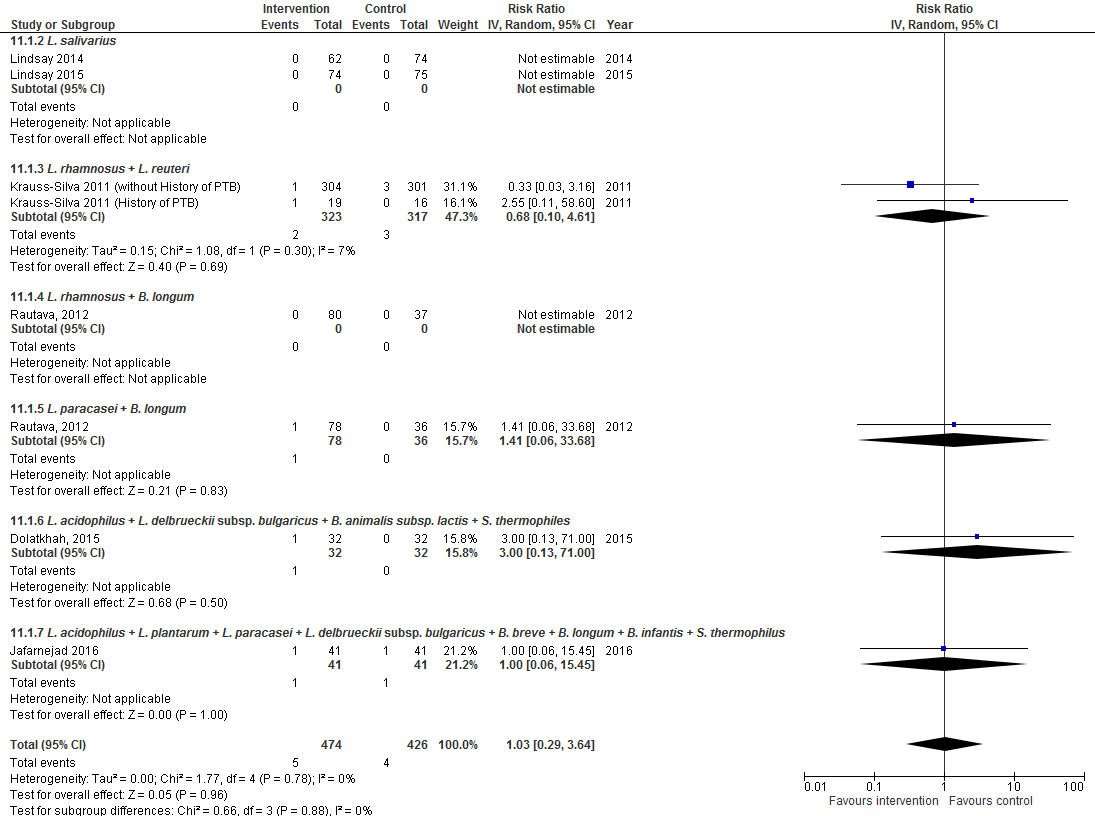


Doloktah 2010: Data provided by authors.

Jafarnejad 2016: Data provided by authors.

Rautava 2012: Data provided by authors. The two intervention groups were combined into one.

#### Preterm birth <37 weeks


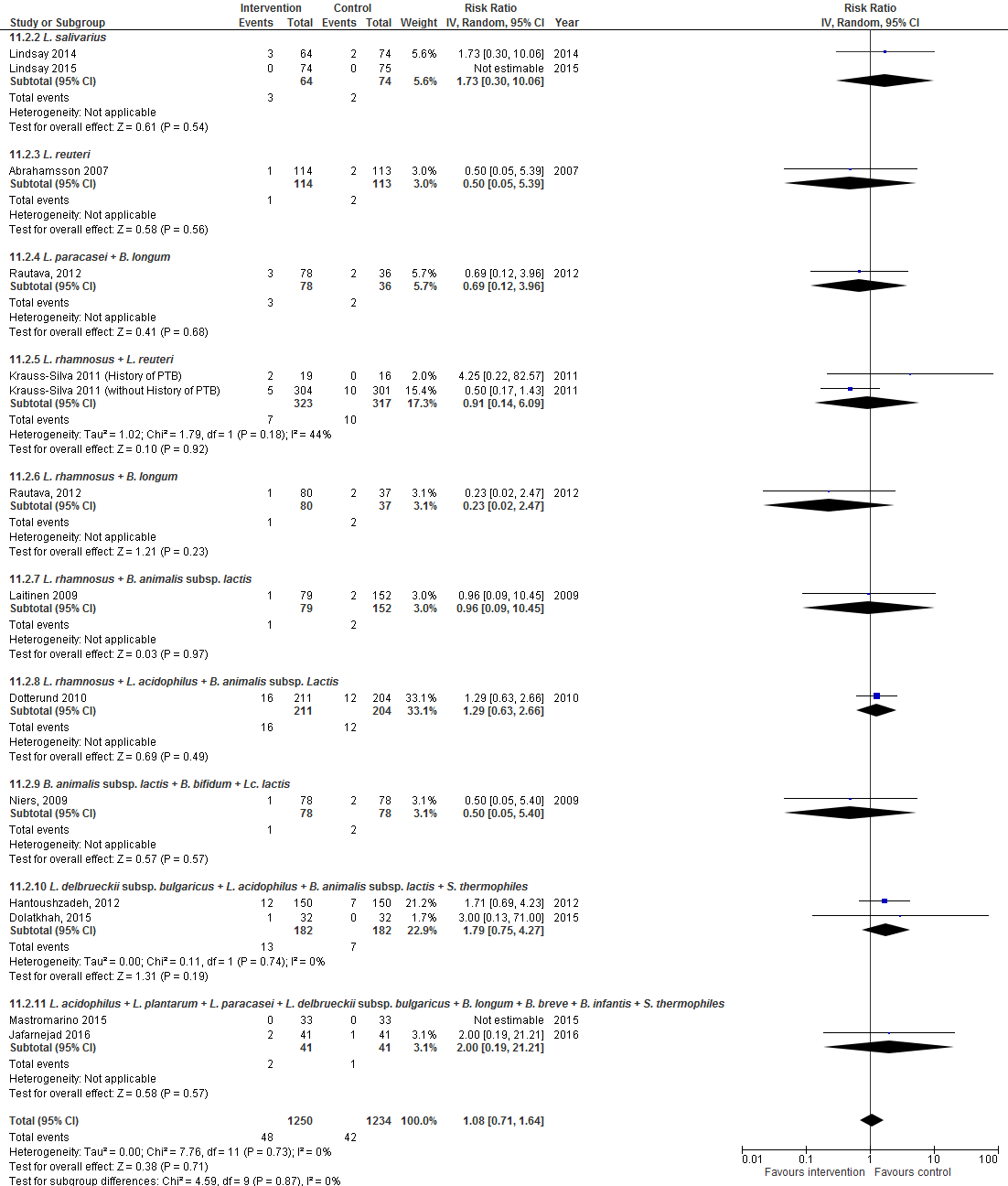


Abrahamsson 2007: Data provided by authors.

Bergmann 2008: The two intervention groups were combined into one.

Dolaktah 2015: Cases excluded from their study due to preterm birth.

Dotterund 2010: Absence of twins not confirmed.

Hantouszhadeh 2012: Absence of twins confirmed by authors.

Jafarnejad 2016: Data provided by authors.

Laitinen 2009: Data provided by authors. The two control groups were combined into one.

Niers 2009: Cases excluded from the study due to preterm birth.

Rautava 2012: Data provided by authors. The two intervention groups were combined into one.

## Subgroup analyses by reported conflicts of interest

#### Preterm birth <34 weeks


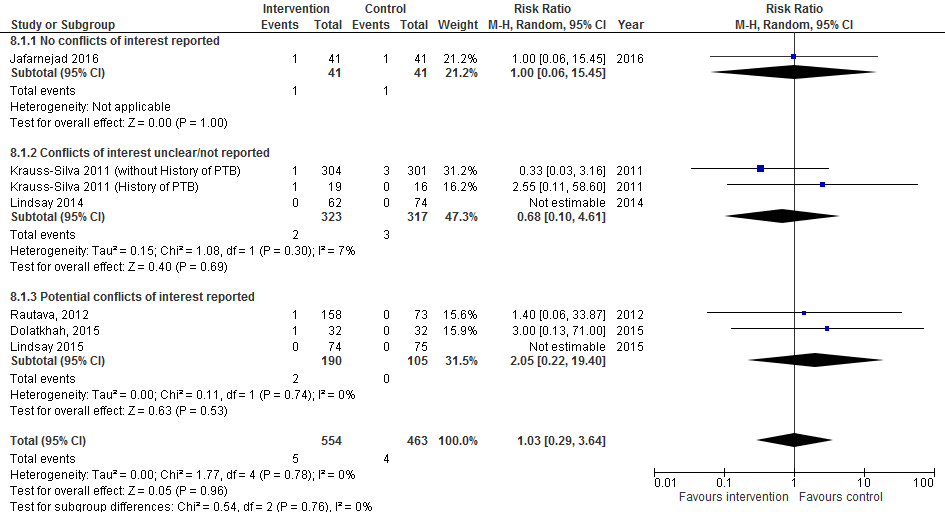


Doloktah 2010: Data provided by authors.

Jafarnejad 2016: Data provided by authors.

Rautava 2012: Data provided by authors. The two intervention groups were combined into one.

#### Preterm birth <37 weeks


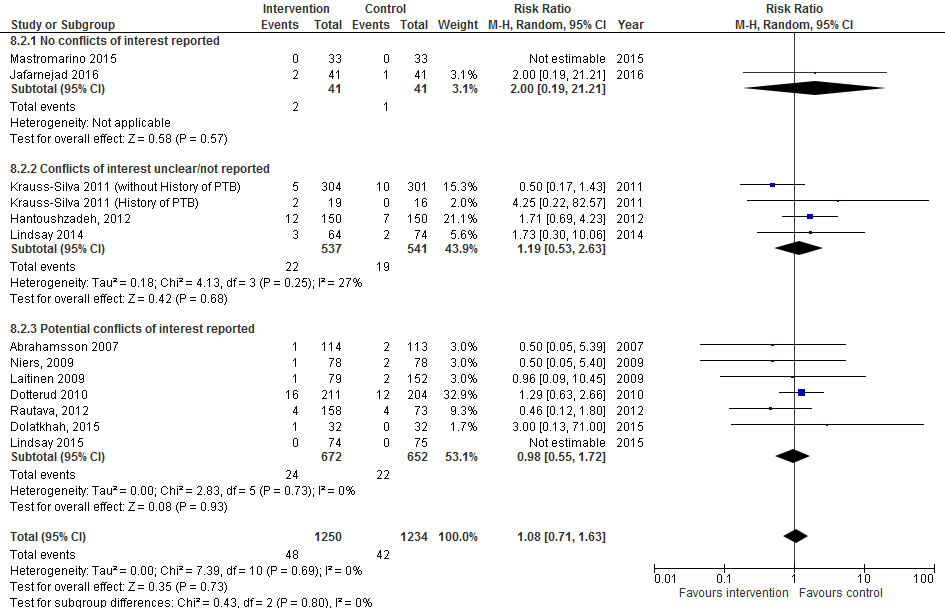


Abrahamsson 2007: Data provided by authors.

Bergmann 2008: The two intervention groups were combined into one.

Dolaktah 2015: Cases excluded from their study due to preterm birth.

Dotterund 2010: Absence of twins not confirmed.

Hantouszhadeh 2012: Absence of twins confirmed by authors.

Jafarnejad 2016: Data provided by authors.

Laitinen 2009: Data provided by authors. The two control groups were combined into one.

Niers 2009: Cases excluded from the study due to preterm birth.

Rautava 2012: Data provided by authors. The two intervention groups were combined into one.

#### Gestational age (weeks)

*
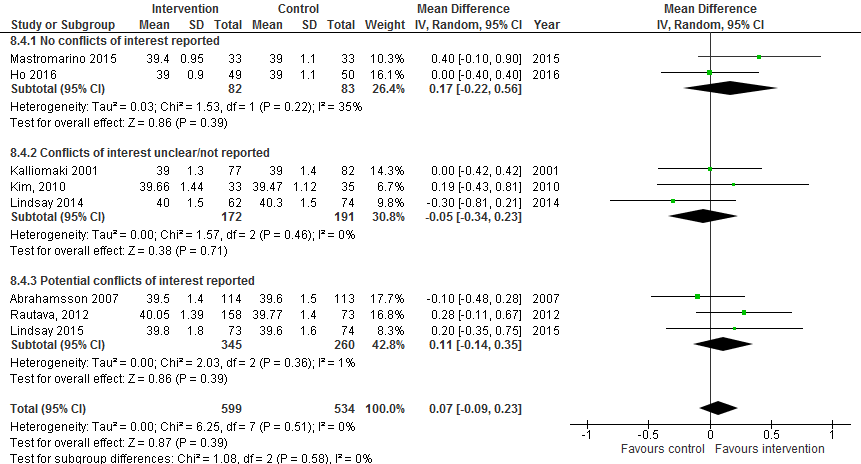
*

Abrahamsson 2007: Data provided by authors.

Bergmann 2008: The two intervention groups were combined into one.

Boyle 2005: Gestational age reported as median weeks (range) and could not be pooled with the other studies: 39.6 weeks (35.4-42) in the probiotics group vs 39.5 weeks (36-42.3) in the control group.

Kim 2010: Subjects were excluded if they had premature babies delivered at less than 36 weeks of gestation, but none of the participants was excluded for this reason. Unclear if singletons only.

Mastromarino 2015: Exclusion criteria included preterm delivery, but only one woman was excluded because she had no milk.

Ou 2012: Gestational age reported as median weeks (range) and could not be pooled with the other studies: 39 weeks (31-41) in the probiotics group vs 39 weeks (35-41) in the control group.

Rautava 2012: Data provided by authors. The two intervention groups were combined into one.

#### Birth weight (grams)


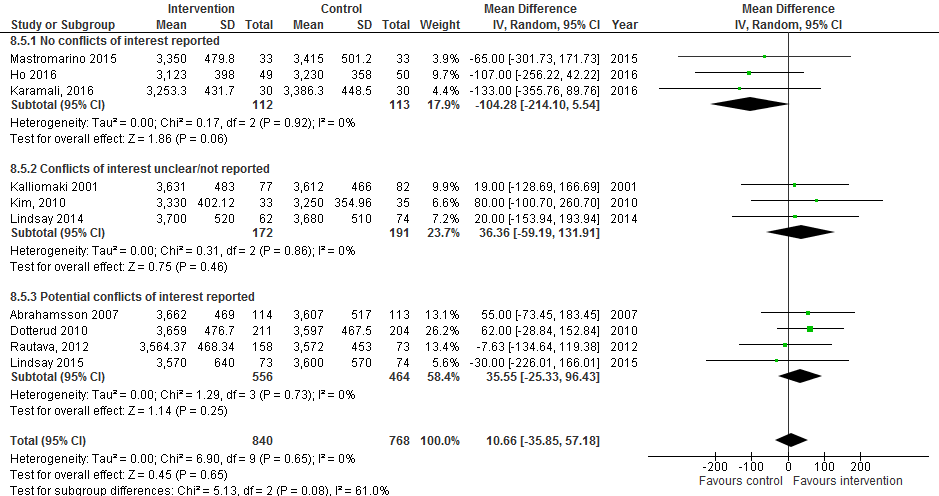


Abrahamsson 2007: Data provided by authors.

Allen 2010: Birth weight reported as median weeks (range) and could not be pooled with the other studies: 3.49 kg (2.1-4.9) in the probiotics group vs 3.55 kg (2-5.2) in the control group.

Bergmann 2008: The two intervention groups were combined into one.

Boyle 2005: Birth weight reported as median weeks (range) and could not be pooled with the other studies: 3560 grams (2324-4970) in the probiotics group vs 3615 grams (2105-5020) in the control group.

Dotterud 2010: Combining Complete cases and drop-outs. Unclear if singletons only.

Karamali 2016: Data (SD) provided by authors.

Kim 2010: Subjects were excluded if they had premature babies delivered at less than 36 weeks of gestation, but none of the participants was excluded for this reason. Unclear if singletons only.

Mastromarino 2015: Exclusion criteria included preterm delivery, but only one woman was excluded because she had no milk.

Rautava 2012: Data provided by authors. The two intervention groups were combined into one.

#### Small for gestational age (<10th percentile for gestational age and sex)


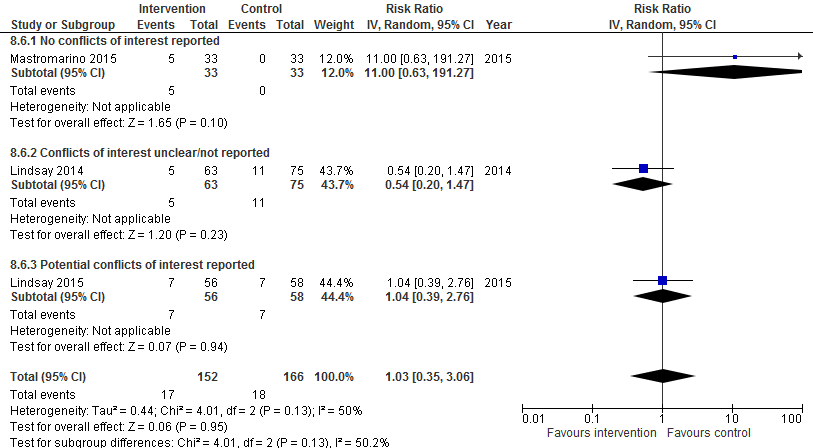


Lindsay 2014: Data provided by authors.

Mastromarino 2015: Data provided by authors.

#### Large for gestational age (>90th percentile for gestational age and sex)


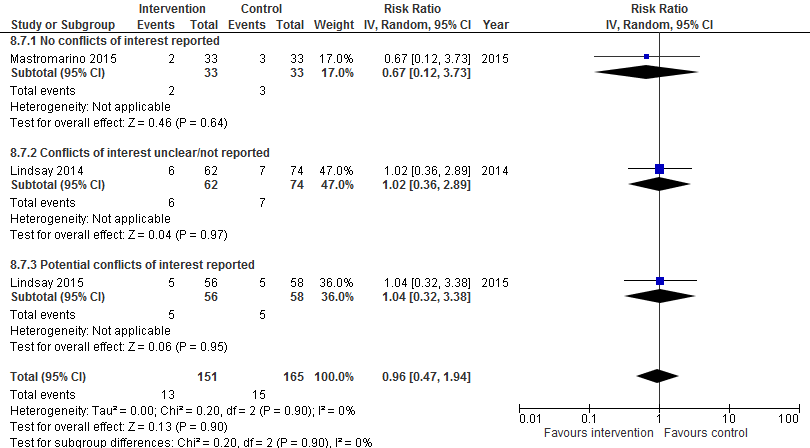


Mastromarino 2015: Data provided by authors.

#### Gestational diabetes mellitus (GDM)


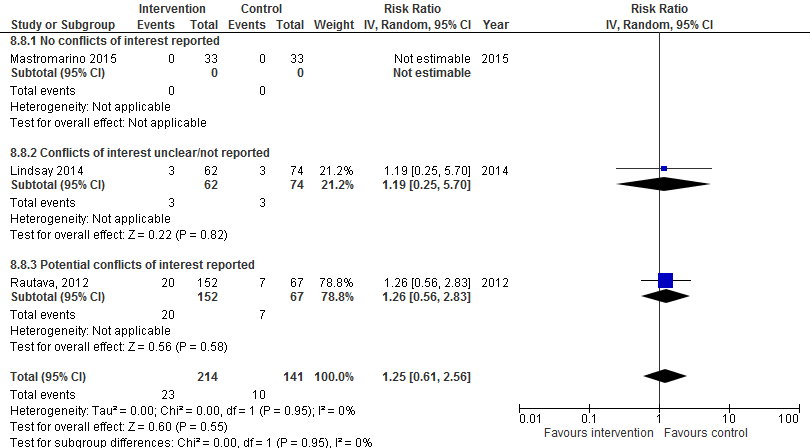


Rautava 2012: Data provided by authors. The two intervention groups were combined into one.

Mastromarino 2015: Data provided by authors.

#### Preterm premature rupture of the membranes (PPROM)


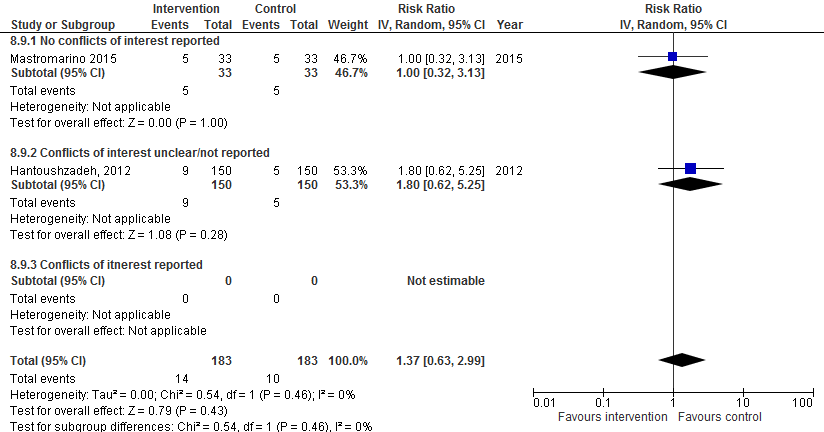


Mastromarino 2015: Data provided by authors.

## Subgroup analyses by intervention lasting up to the end of pregnancy or not

#### Preterm birth <34 weeks


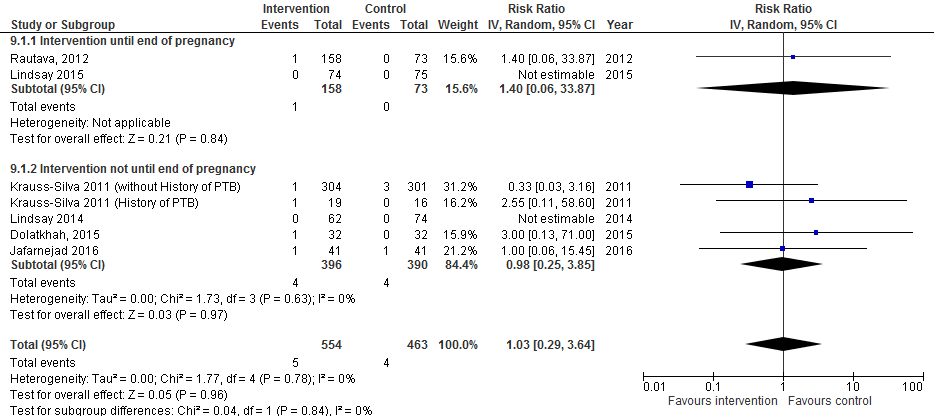


Doloktah 2010: Data provided by authors.

Jafarnejad 2016: Data provided by authors.

Rautava 2012: Data provided by authors. The two intervention groups were combined into one.

#### Preterm birth <37 weeks


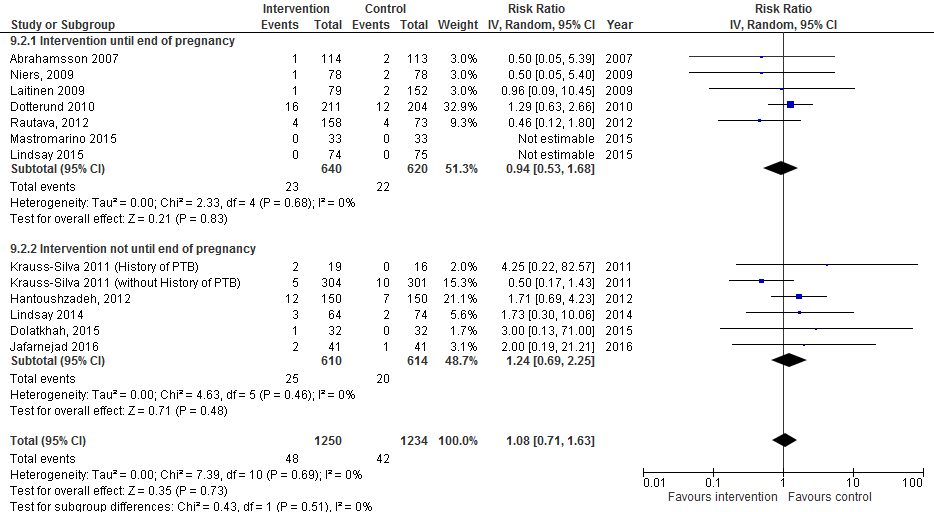


Abrahamsson 2007: Data provided by authors.

Bergmann 2008: The two intervention groups were combined into one.

Dolaktah 2015: Cases excluded from their study due to preterm birth.

Dotterund 2010: Absence of twins not confirmed.

Hantouszhadeh 2012: Absence of twins confirmed by authors.

Jafarnejad 2016: Data provided by authors.

Laitinen 2009: Data provided by authors. The two control groups were combined into one.

Niers 2009: Cases excluded from the study due to preterm birth.

Rautava 2012: Data provided by authors. The two intervention groups were combined into one.

#### Gestational age (weeks)


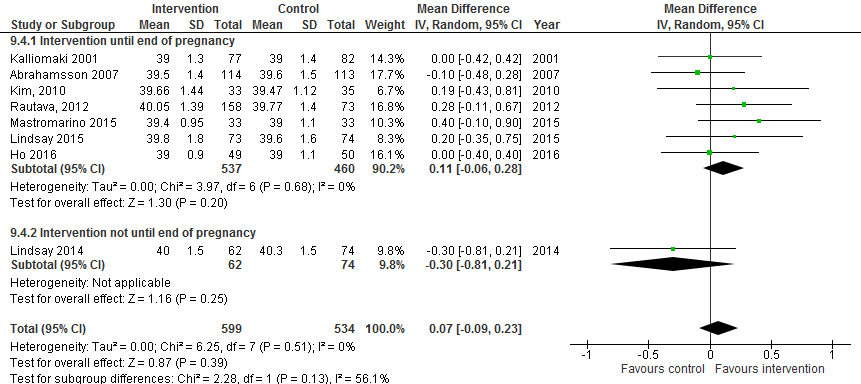


Abrahamsson 2007: Data provided by authors.

Bergmann 2008: The two intervention groups were combined into one.

Boyle 2005: Gestational age reported as median weeks (range) and could not be pooled with the other studies: 39.6 weeks (35.4-42) in the probiotics group vs 39.5 weeks (36-42.3) in the control group.

Kim 2010: Subjects were excluded if they had premature babies delivered at less than 36 weeks of gestation, but none of the participants was excluded for this reason. Unclear if singletons only.

Mastromarino 2015: Exclusion criteria included preterm delivery, but only one woman was excluded because she had no milk.

Ou 2012: Gestational age reported as median weeks (range) and could not be pooled with the other studies: 39 weeks (31-41) in the probiotics group vs 39 weeks (35-41) in the control group.

Rautava 2012: Data provided by authors. The two intervention groups were combined into one.

#### Birth weight (grams)


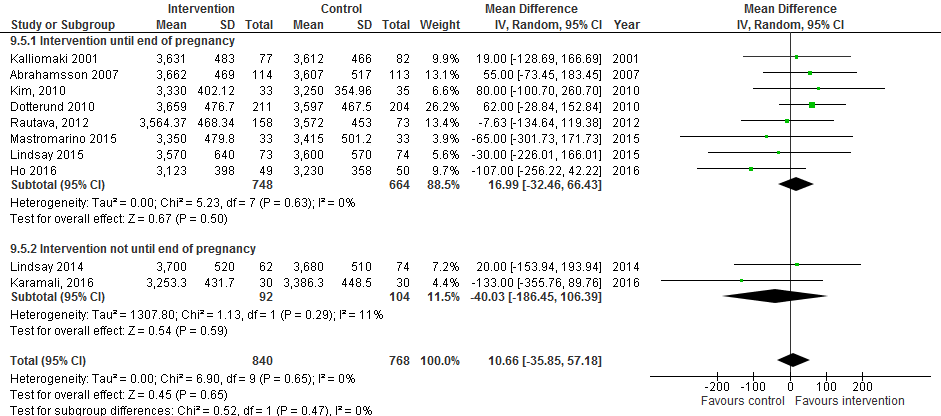


Abrahamsson 2007: Data provided by authors.

Allen 2010: Birth weight reported as median weeks (range) and could not be pooled with the other studies: 3.49 kg (2.1-4.9) in the probiotics group vs 3.55 kg (2-5.2) in the control group.

Bergmann 2008: The two intervention groups were combined into one.

Boyle 2005: Birth weight reported as median weeks (range) and could not be pooled with the other studies: 3560 grams (2324-4970) in the probiotics group vs 3615 grams (2105-5020) in the control group.

Dotterud 2010: Combining Complete cases and drop-outs. Unclear if singletons only.

Karamali 2016: Data (SD) provided by authors.

Kim 2010: Subjects were excluded if they had premature babies delivered at less than 36 weeks of gestation, but none of the participants was excluded for this reason. Unclear if singletons only.

Mastromarino 2015: Exclusion criteria included preterm delivery, but only one woman was excluded because she had no milk.

Rautava 2012: Data provided by authors. The two intervention groups were combined into one.

#### Small for gestational age (<10th percentile for gestational age and sex)


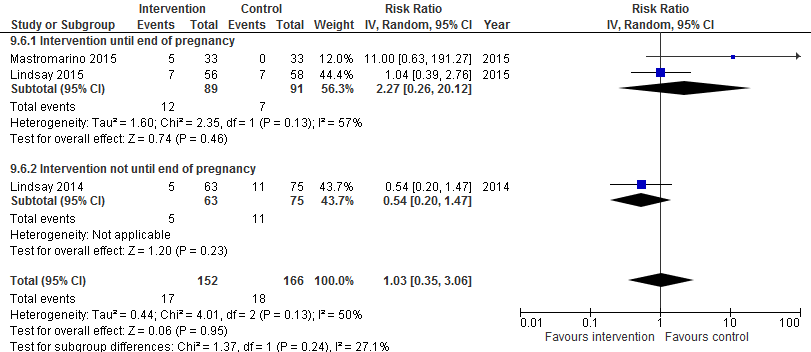


Lindsay 2014: Data provided by authors.

Mastromarino 2015: Data provided by authors.

#### Large for gestational age (>90th percentile for gestational age and sex)


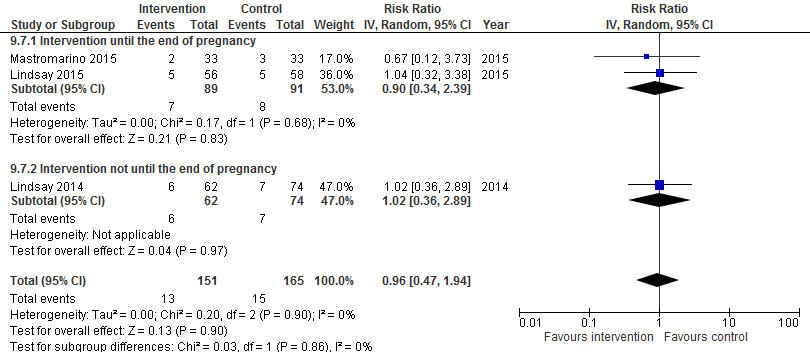


Mastromarino 2015: Data provided by authors.

#### Gestational diabetes mellitus (GDM)


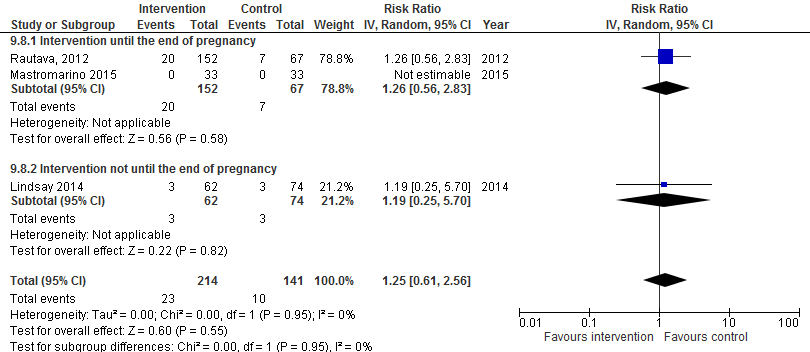


Rautava 2012: Data provided by authors. The two intervention groups were combined into one.

Mastromarino 2015: Data provided by authors.

#### Preterm premature rupture of the membranes (PPROM)


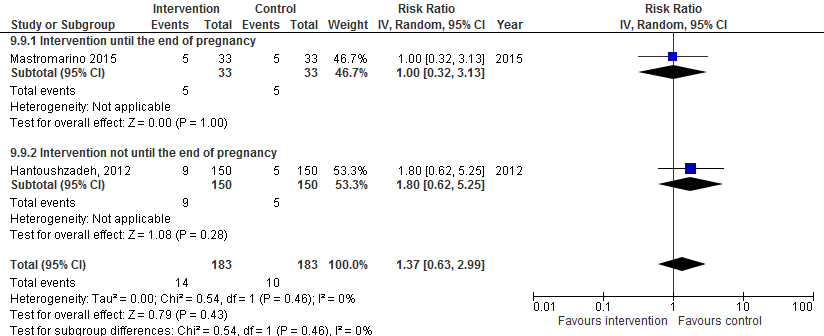


Mastromarino 2015: Data provided by authors.

## Sensitivity analyses

### Excluding studies without confirmation of singletons only

#### Preterm birth <34 weeks


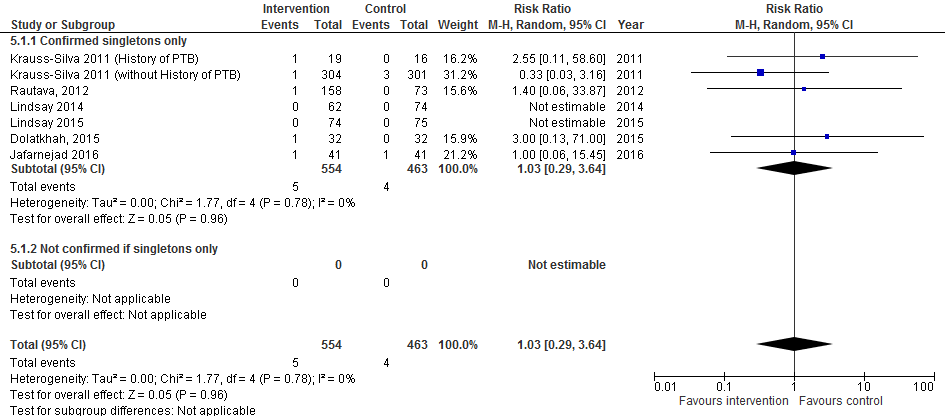


Doloktah 2010: Data provided by authors.

Jafarnejad 2016: Data provided by authors.

Rautava 2012: Data provided by authors. The two intervention groups were combined into one.

#### Preterm birth <37 weeks


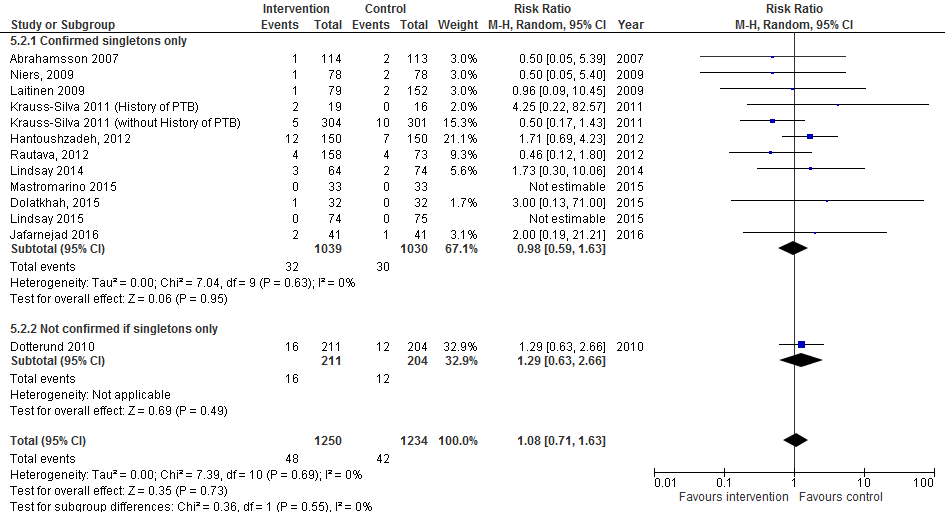


Abrahamsson 2007: Data provided by authors.

Dolaktah 2015: Cases excluded from their study due to preterm birth.

Dotterund 2010: Absence of twins not confirmed.

Hantouszhadeh 2012: Absence of twins confirmed by authors.

Jafarnejad 2016: Data provided by authors.

Laitinen 2009: Data provided by authors. The two control groups were combined into one.

Niers 2009: Cases excluded from the study due to preterm birth.

Rautava 2012: Data provided by authors. The two intervention groups were combined into one.

#### Gestational age (weeks)


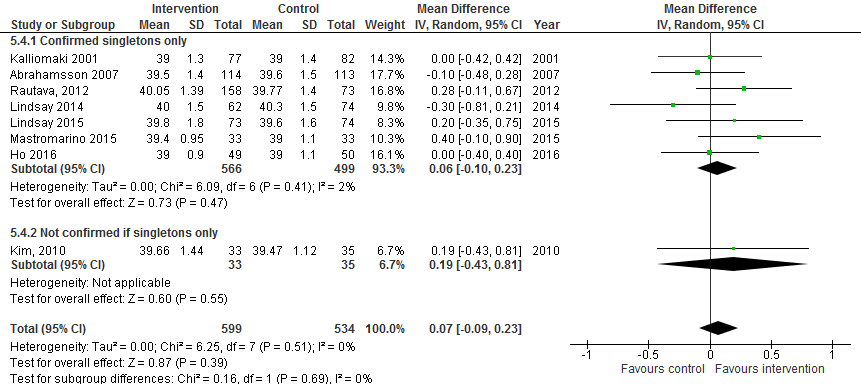


Abrahamsson 2007: Data provided by authors.

Boyle 2005: Gestational age reported as median weeks (range) and could not be pooled with the other studies: 39.6 weeks (35.4-42) in the probiotics group vs 39.5 weeks (36-42.3) in the control group.

Kim 2010: Subjects were excluded if they had premature babies delivered at less than 36 weeks of gestation, but none of the participants was excluded for this reason. Unclear if singletons only.

Mastromarino 2015: Exclusion criteria included preterm delivery, but only one woman was excluded because she had no milk.

Ou 2012: Gestational age reported as median weeks (range) and could not be pooled with the other studies: 39 weeks (31-41) in the probiotics group vs 39 weeks (35-41) in the control group.

Rautava 2012: Data provided by authors. The two intervention groups were combined into one.

#### Birth weight (grams)


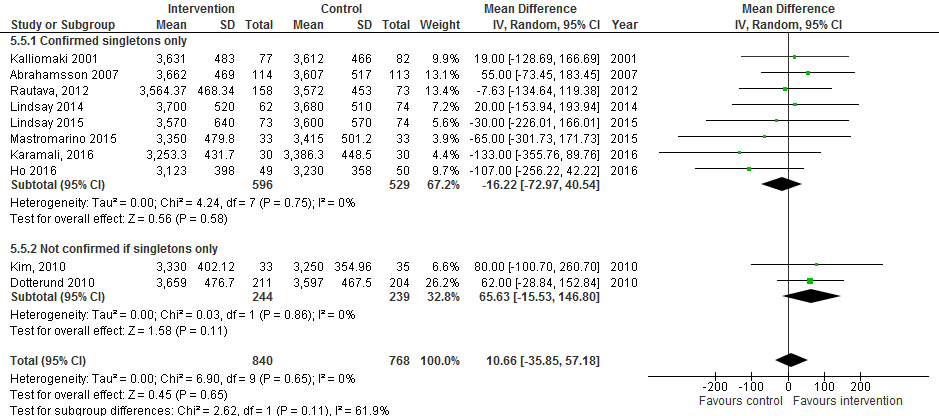


Abrahamsson 2007: Data provided by authors.

Allen 2010: Birth weight reported as median weeks (range) and could not be pooled with the other studies: 3.49 kg (2.1-4.9) in the probiotics group vs 3.55 kg (2-5.2) in the control group.

Boyle 2005: Birth weight reported as median weeks (range) and could not be pooled with the other studies: 3560 grams (2324-4970) in the probiotics group vs 3615 grams (2105-5020) in the control group.

Dotterud 2010: Combining Complete cases and drop-outs. Unclear if singletons only.

Karamali 2016: Data (SD) provided by authors.

Kim 2010: Subjects were excluded if they had premature babies delivered at less than 36 weeks of gestation, but none of the participants was excluded for this reason. Unclear if singletons only.

Mastromarino 2015: Exclusion criteria included preterm delivery, but only one woman was excluded because she had no milk.

Rautava 2012: Data provided by authors. The two intervention groups were combined into one.

#### Small for gestational age (<10th percentile for gestational age and sex)


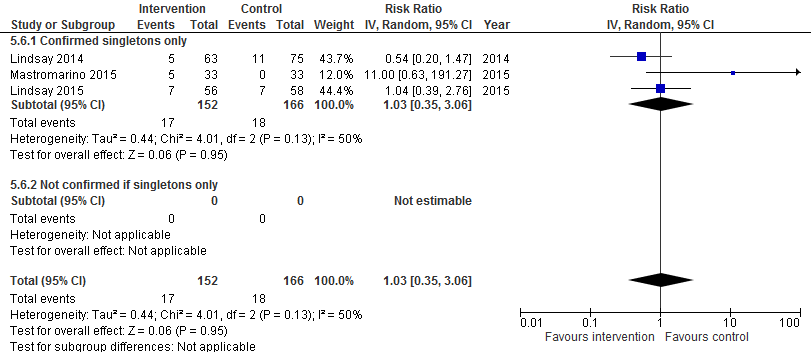


Lindsay 2014: Data provided by authors.

Mastromarino 2015: Data provided by authors.

#### Large for gestational age (>90th percentile for gestational age and sex)


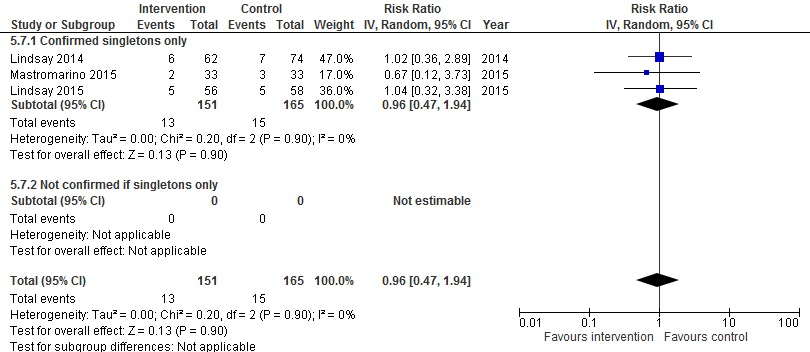


Mastromarino 2015: Data provided by authors.

#### Gestational diabetes mellitus (GDM)


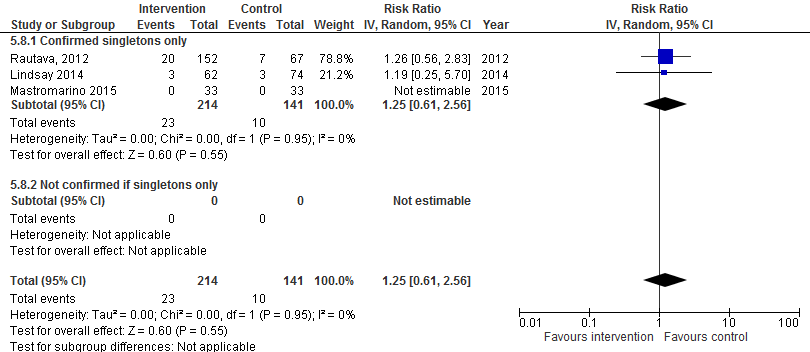


Rautava 2012: Data provided by authors. The two intervention groups were combined into one.

Mastromarino 2015: Data provided by authors.

#### Preterm premature rupture of the membranes (PPROM)


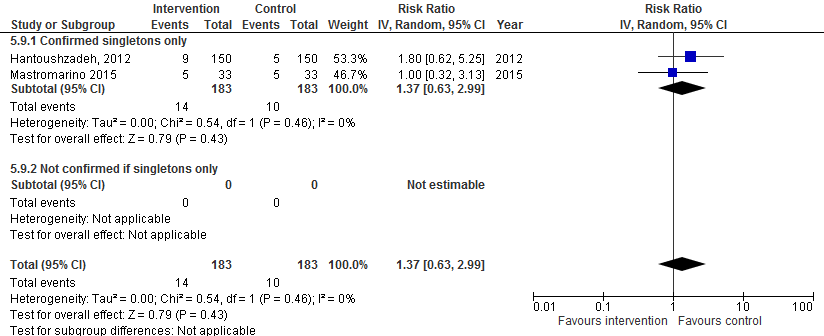


Mastromarino 2015: Data provided by authors.

### Combining multiple independent comparisons in one study using fixed-effects meta-analysis before pooling with rest of studies

#### Preterm birth <34 weeks


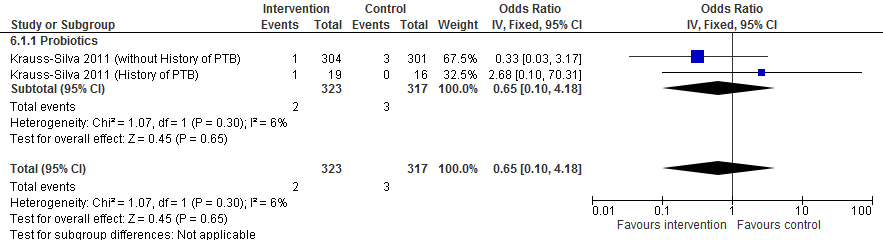


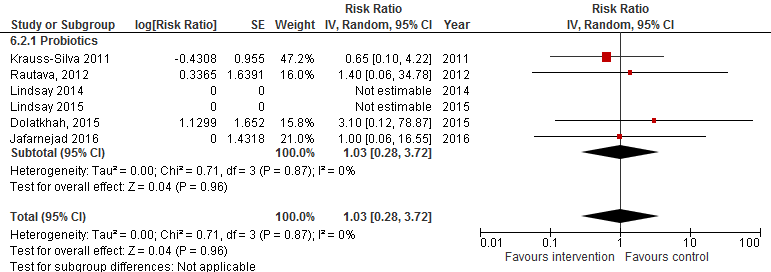


Doloktah 2010: Data provided by authors.

Jafarnejad 2016: Data provided by authors.

Rautava 2012: Data provided by authors. The two intervention groups were combined into one.

#### Preterm birth <37 weeks


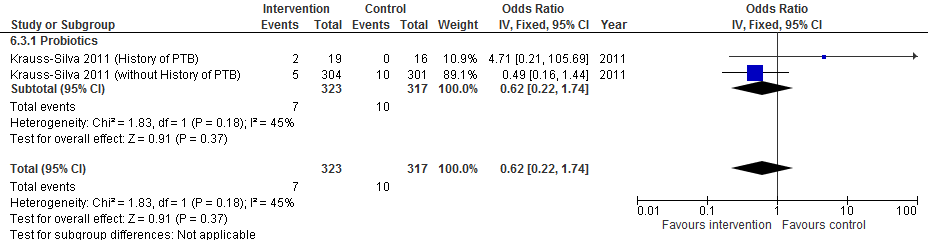


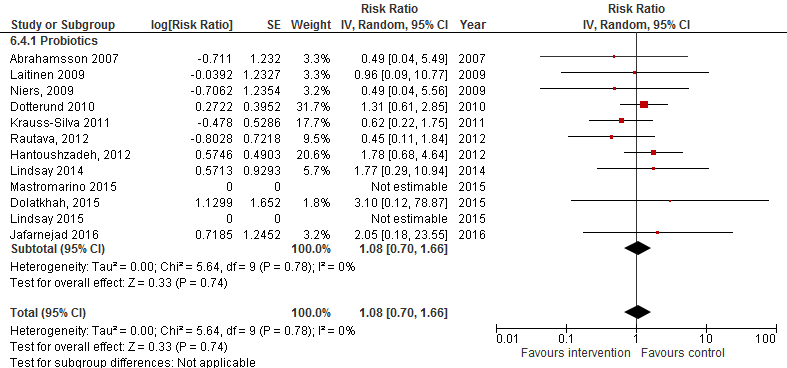


Abrahamsson 2007: Data provided by authors.

Dolaktah 2015: Cases excluded from their study due to preterm birth.

Dotterund 2010: Absence of twins not confirmed.

Hantouszhadeh 2012: Absence of twins confirmed by authors.

Jafarnejad 2016: Data provided by authors.

Laitinen 2009: Data provided by authors. The two control groups were combined into one.

Niers 2009: Cases excluded from the study due to preterm birth.

Rautava 2012: Data provided by authors. The two intervention groups were combined into one.

### Excluding studies with unclear or high risk of bias

#### Preterm birth <34 weeks


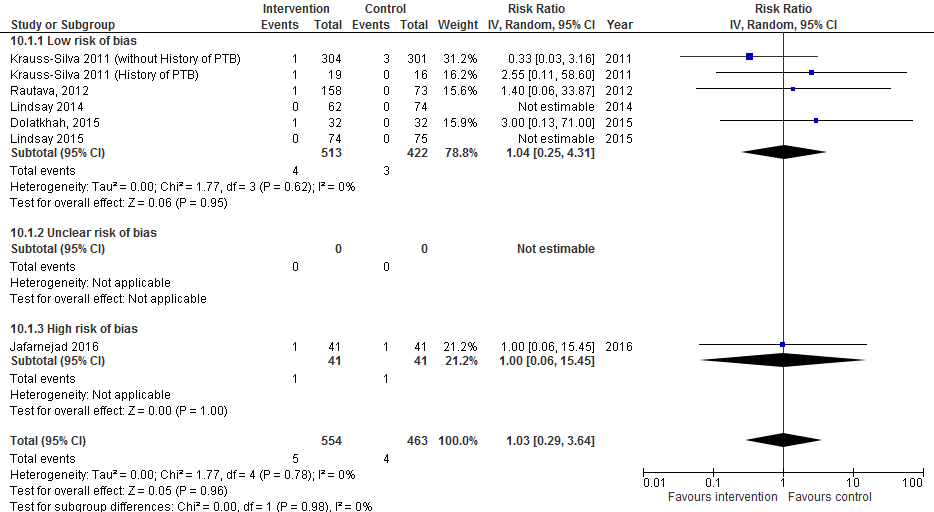


Doloktah 2010: Data provided by authors.

Jafarnejad 2016: Data provided by authors.

Rautava 2012: Data provided by authors. The two intervention groups were combined into one.

#### Preterm birth <37 weeks


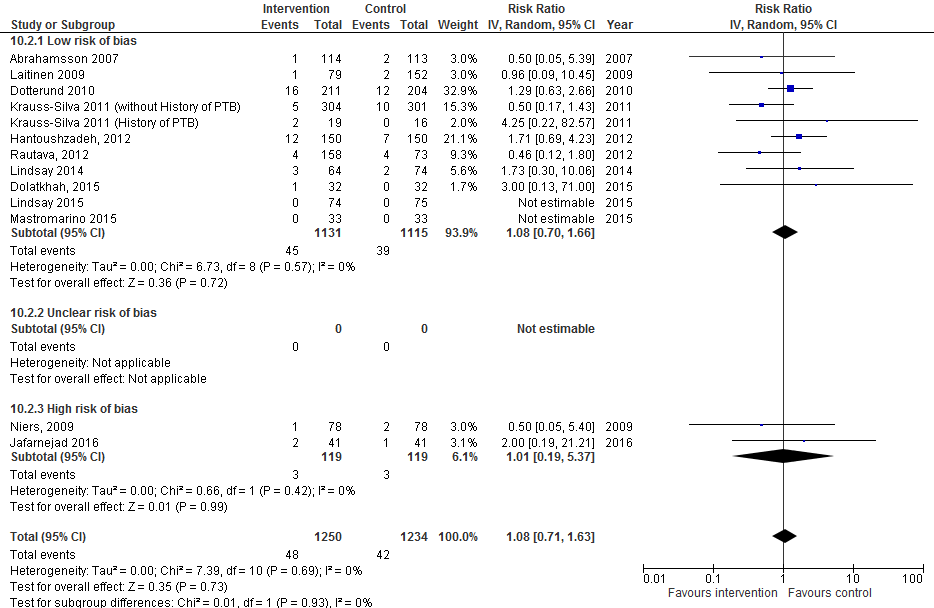


Abrahamsson 2007: Data provided by authors.

Dolaktah 2015: Cases excluded from their study due to preterm birth.

Dotterund 2010: Absence of twins not confirmed.

Hantouszhadeh 2012: Absence of twins confirmed by authors.

Jafarnejad 2016: Data provided by authors.

Laitinen 2009: Data provided by authors. The two control groups were combined into one.

Niers 2009: Cases excluded from the study due to preterm birth.

Rautava 2012: Data provided by authors. The two intervention groups were combined into one.

#### Gestational age (weeks)


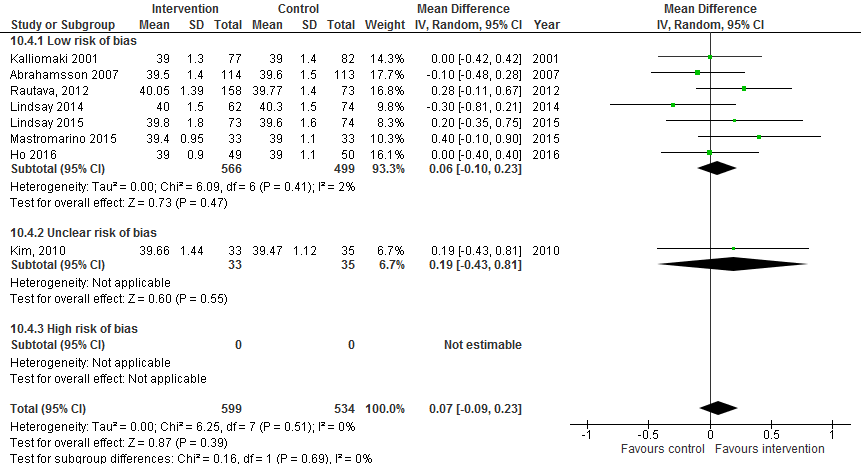


Abrahamsson 2007: Data provided by authors.

Boyle 2005: Gestational age reported as median weeks (range) and could not be pooled with the other studies: 39.6 weeks (35.4-42) in the probiotics group vs 39.5 weeks (36-42.3) in the control group.

Kim 2010: Subjects were excluded if they had premature babies delivered at less than 36 weeks of gestation, but none of the participants was excluded for this reason. Unclear if singletons only.

Mastromarino 2015: Exclusion criteria included preterm delivery, but only one woman was excluded because she had no milk.

Ou 2012: Gestational age reported as median weeks (range) and could not be pooled with the other studies: 39 weeks (31-41) in the probiotics group vs 39 weeks (35-41) in the control group.

Rautava 2012: Data provided by authors. The two intervention groups were combined into one.

#### Birth weight (grams)


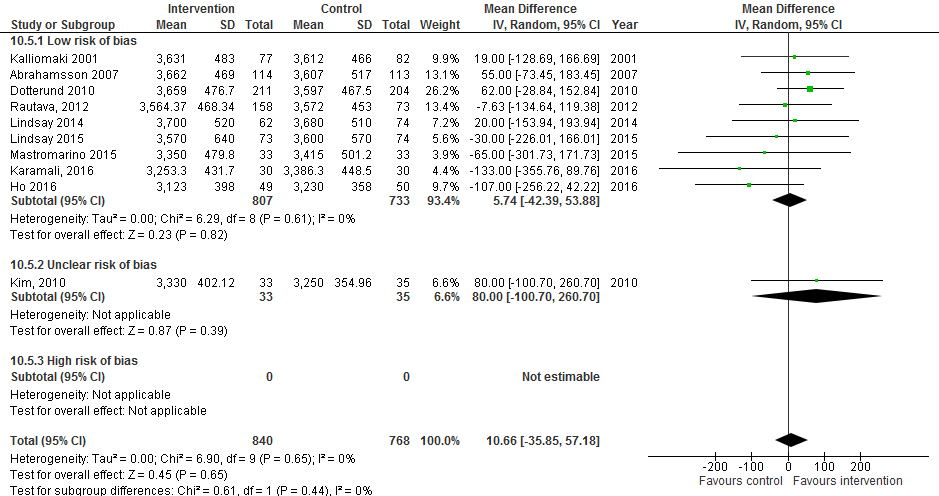


Abrahamsson 2007: Data provided by authors.

Allen 2010: Birth weight reported as median weeks (range) and could not be pooled with the other studies: 3.49 kg (2.1-4.9) in the probiotics group vs 3.55 kg (2-5.2) in the control group.

Boyle 2005: Birth weight reported as median weeks (range) and could not be pooled with the other studies: 3560 grams (2324-4970) in the probiotics group vs 3615 grams (2105-5020) in the control group.

Dotterud 2010: Combining Complete cases and drop-outs. Unclear if singletons only.

Karamali 2016: Data (SD) provided by authors.

Kim 2010: Subjects were excluded if they had premature babies delivered at less than 36 weeks of gestation, but none of the participants was excluded for this reason. Unclear if singletons only.

Mastromarino 2015: Exclusion criteria included preterm delivery, but only one woman was excluded because she had no milk.

Rautava 2012: Data provided by authors. The two intervention groups were combined into one.

#### Small for gestational age (<10th percentile for gestational age and sex)


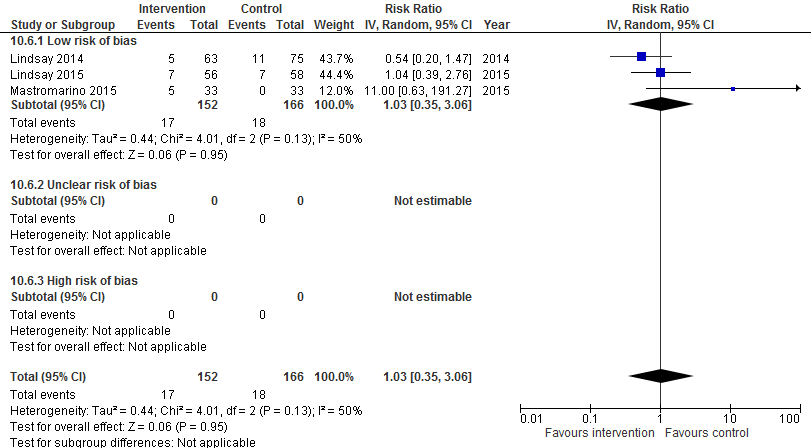


Lindsay 2014: Data provided by authors.

Mastromarino 2015: Data provided by authors.

#### Large for gestational age (>90th percentile for gestational age and sex)


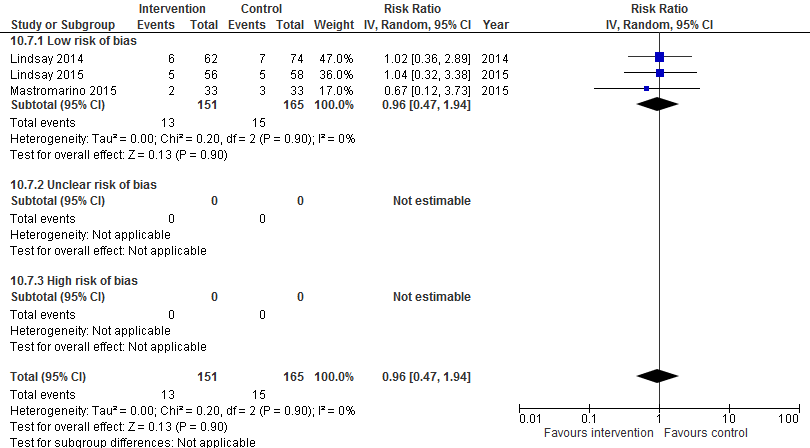


Mastromarino 2015: Data provided by authors.

#### Gestational diabetes mellitus (GDM)


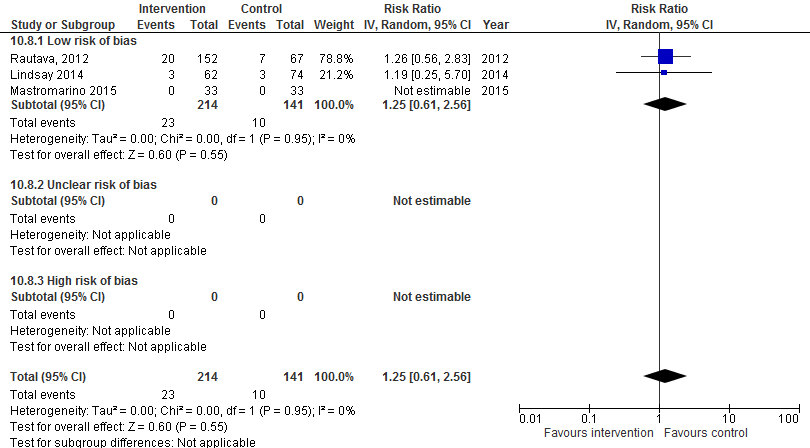


Rautava 2012: Data provided by authors. The two intervention groups were combined into one.

Mastromarino 2015: Data provided by authors.

#### Preterm premature rupture of the membranes (PPROM)


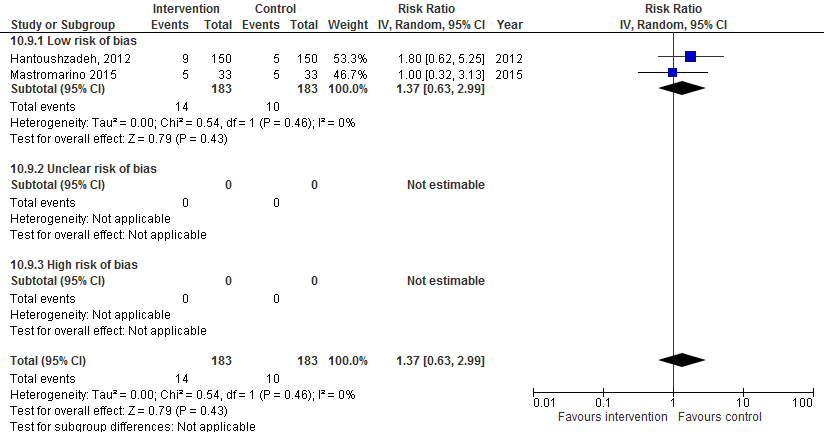


Mastromarino 2015: Data provided by authors.

### Excluding study with conventional yogurt (with starter cells of probiotics) as control group

#### Fasting plasma glucose (FPG) (mg/dL) (using correlation in Jafernejad 2016)


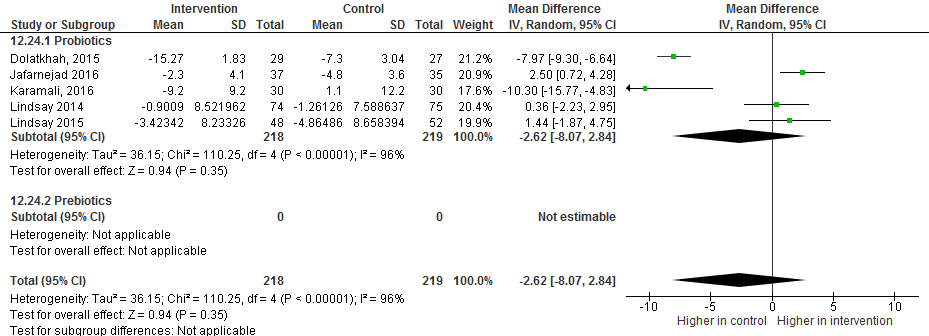


Lindsay 2014: SD of the measures of change were inputed.

Lindsay 2015: SD of the measures of change were inputed. Per protocol cohort.

#### HOMA insulin resistance (using correlation in Jafernejad 2016)


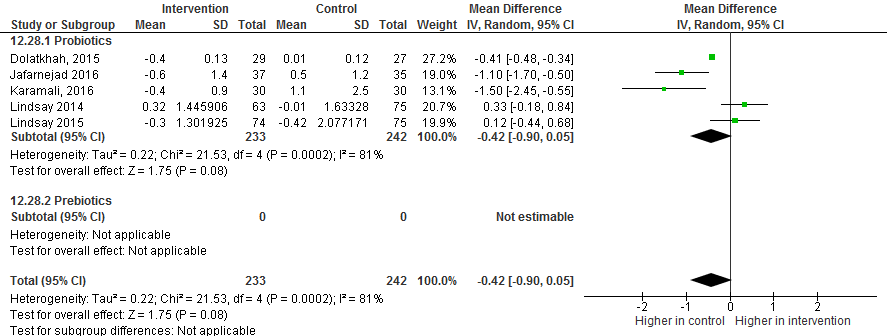


Lindsay 2014: SD of the measures of change were inputed.

Lindsay 2015: SD of the measures of change were inputed. Per protocol cohort.

#### Insulin (𝜇IU/mL) (using correlation in Jafernejad 2016)


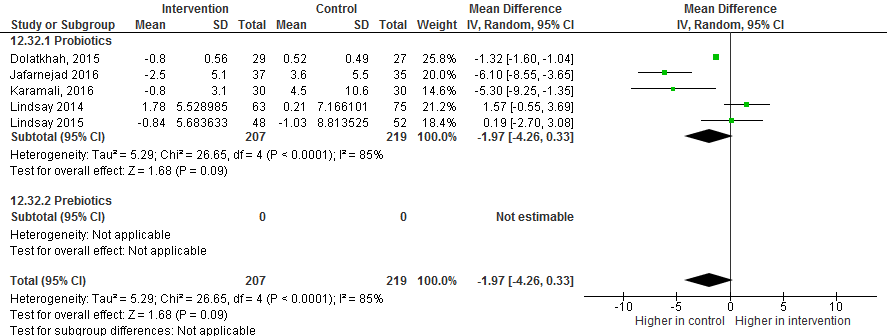


Lindsay 2014: SD of the measures of change were inputed.

Lindsay 2015: SD of the measures of change were inputed. Per protocol cohort.

### Inputing data using correlation values of different studies

#### Fasting plasma glucose (FPG) (mg/dL) (using correlation in Asemi 2011)


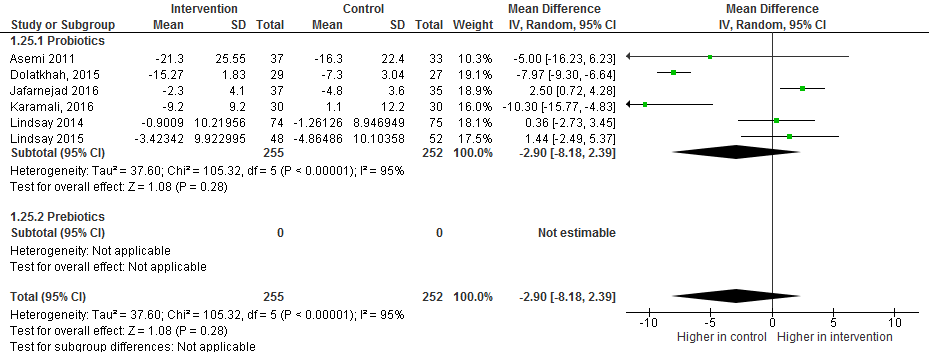


Lindsay 2014: SD of the measures of change were inputed.

Lindsay 2015: SD of the measures of change were inputed. Per protocol cohort.

#### Fasting plasma glucose (FPG) (mg/dL) (using correlation in Doloktah 2011)


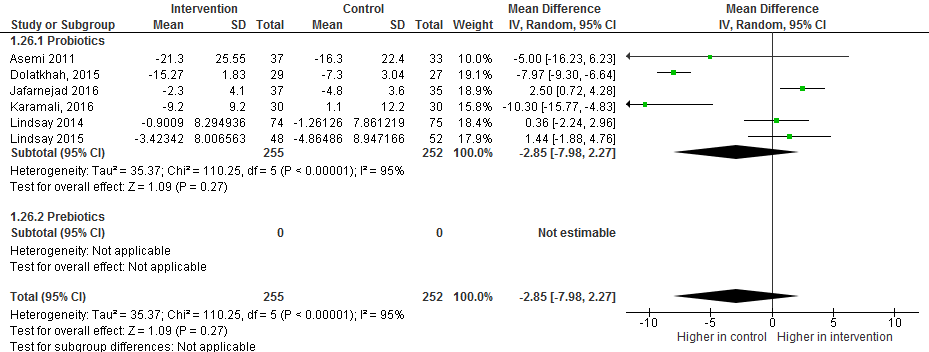


Lindsay 2014: SD of the measures of change were inputed.

Lindsay 2015: SD of the measures of change were inputed. Per protocol cohort.

#### Fasting plasma glucose (FPG) (mg/dL) (using correlation in Karamali 2016)


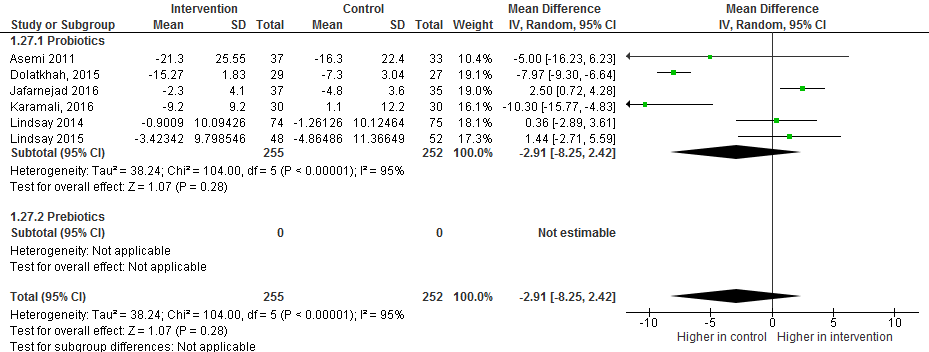


Lindsay 2014: SD of the measures of change were inputed.

Lindsay 2015: SD of the measures of change were inputed. Per protocol cohort.

#### HOMA insulin resistance (using correlation in Asemi 2011)


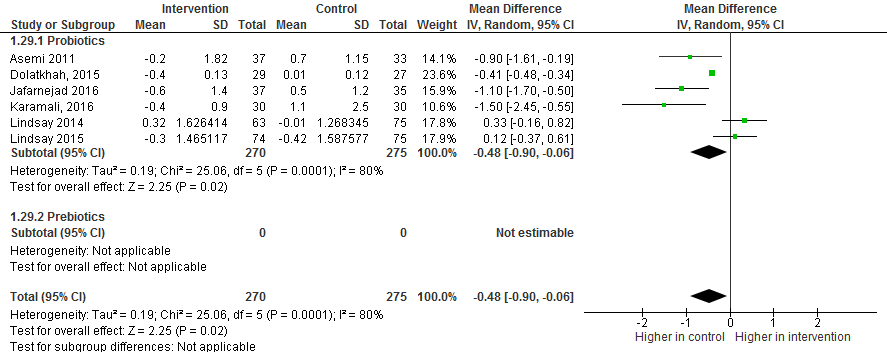


Lindsay 2014: SD of the measures of change were inputed.

Lindsay 2015: SD of the measures of change were inputed. Per protocol cohort.

#### HOMA insulin resistance (using correlation in Doloktah 2011)


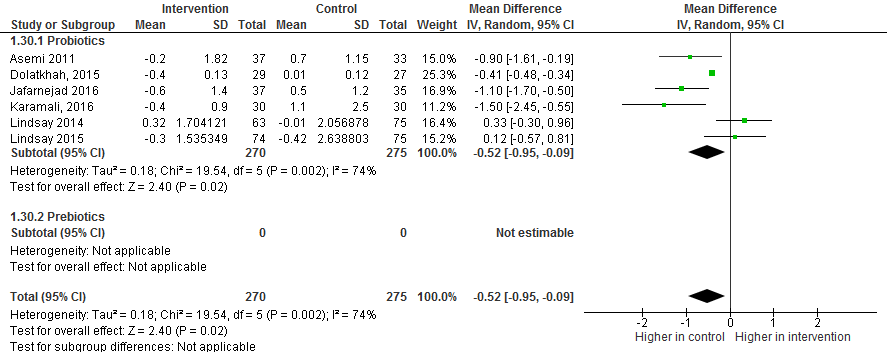


Lindsay 2014: SD of the measures of change were inputed.

Lindsay 2015: SD of the measures of change were inputed. Per protocol cohort.

#### HOMA insulin resistance (using correlation in Karamali 2016)


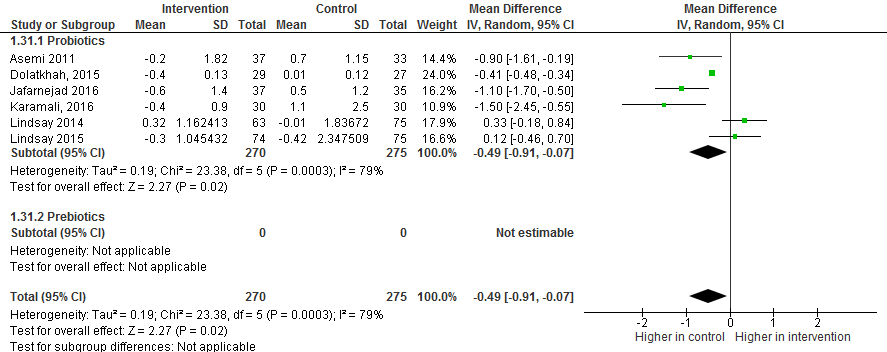


Lindsay 2014: SD of the measures of change were inputed.

Lindsay 2015: SD of the measures of change were inputed. Per protocol cohort.

#### Insulin (𝜇IU/mL) (using correlation in Asemi 2011)


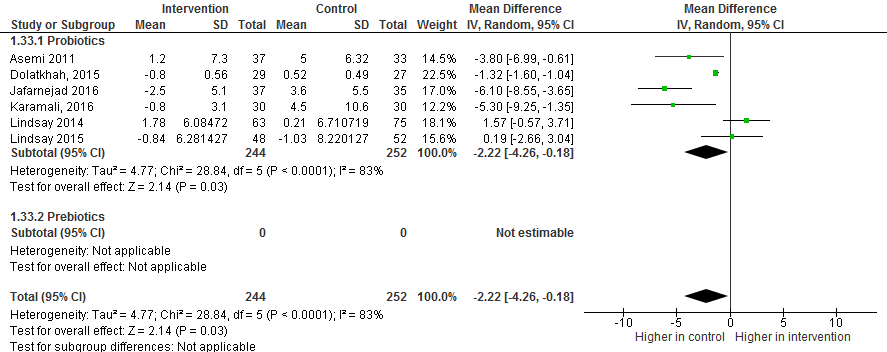


Lindsay 2014: SD of the measures of change were inputed.

Lindsay 2015: SD of the measures of change were inputed. Per protocol cohort.

#### Insulin (𝜇IU/mL) (using correlation in Doloktah 2011)


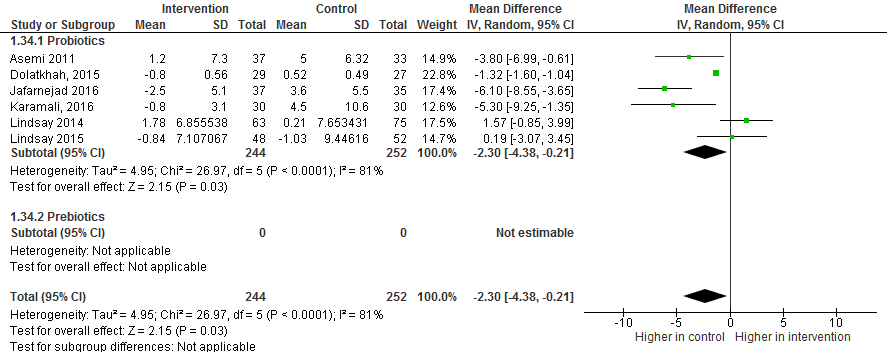


Lindsay 2014: SD of the measures of change were inputed.

Lindsay 2015: SD of the measures of change were inputed. Per protocol cohort.

#### Insulin (𝜇IU/mL) (using correlation in Karamali 2016)


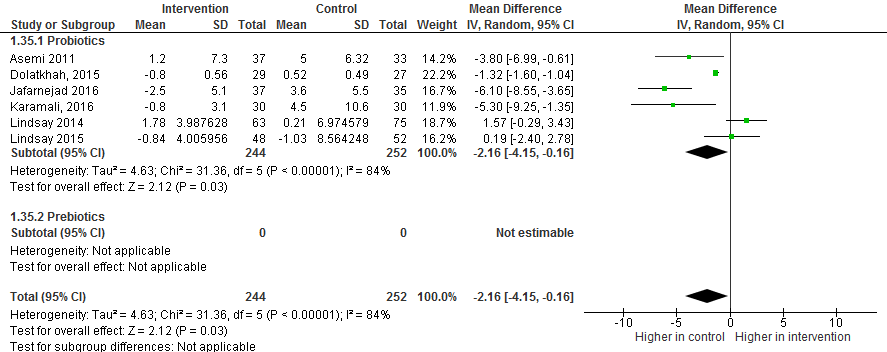


Lindsay 2014: SD of the measures of change were inputed.

Lindsay 2015: SD of the measures of change were inputed. Per protocol cohort.
